# Chemical design of monolayer altermagnets

**DOI:** 10.1093/nsr/nwaf528

**Published:** 2025-11-22

**Authors:** Runzhang Xu, Yifan Gao, Junwei Liu

**Affiliations:** Department of Physics, The Hong Kong University of Science and Technology, Hong Kong, China; Department of Physics, The Hong Kong University of Science and Technology, Hong Kong, China; Department of Physics, The Hong Kong University of Science and Technology, Hong Kong, China

**Keywords:** altermagnet, monolayer material, materials design, first-principles calculation

## Abstract

The crystal-symmetry-paired spin-momentum locking (CSML), arising from the intrinsic crystal symmetry that connects different magnetic sublattices in altermagnets, enables many exotic spintronics properties, such as unconventional piezomagnetism and non-collinear spin currents. However, the shortage of monolayer altermagnets restricts further exploration of dimensionally confined phenomena and applications of nanostructured devices. Here, we propose general chemical design principles inspired by sublattice symmetry of the layered altermagnet V$_2$(Se,Te)$_2$O through symmetry-preserving structural modification and valence-adaptive chemical substitutions. In total, we construct 2600 candidates across four structural frameworks, M$_2$A$_2$B$_{1,0}$ and their Janus derivatives. High-throughput calculations identify 612 potential altermagnets with Néel-ordered ground states, among which 79 exhibit CSML Dirac cones that enable spin-polarized ultra-fast transport. These materials also feature different ground-state magnetic orderings and demonstrate diverse electronic behaviors, ranging from semiconductors and metals to half-metals and Dirac semimetals. This work not only reveals abundant monolayer altermagnets, but also establishes a rational principle for their design, opening the gates to the exploration of confined magnetism and spintronics in atomically thin systems.

## Teaser

Symmetry-guided chemical design and high-throughput calculations predict a wide array of highly realizable monolayer altermagnets that host C-paired spin-momentum locking and Dirac-cone energy bands, advancing the development of spintronics.

## INTRODUCTION

Altermagnets simultaneously feature both spin-splitting band structures with crystal-symmetry-paired spin-momentum locking (CSML) in momentum space and antiferromagnetic (AFM) ordering in real space due to the intrinsic crystal symmetries that map magnetic sublattices of opposite spins to each other [1–11]. These features give rise to unique properties, including unconventional piezomagnetism [1,6] and non-collinear spin current [1,2,6], as well as exotic multiferroicity when coupled with ferroelectricity [12–14], which not only advances our understanding of unconventional magnetism but also promises many novel applications [15–17]. Several candidates for real materials have been confirmed in experiments [15,16], although most of them are bulk crystals or thin films, with the only layered example being V$_2$(Se,Te)$_2$O [6,18,19]. The lack of two-dimensional (2D), in particular monolayer, altermagnetic (AM) materials strongly hinders the further investigation of novel phenomena and exotic physics arising from reduced dimensionality and strong correlations, as well as their susceptibility to external modulations (e.g. strain, doping and/or electric gating), proximity effects from superconducting or topological phases and their facile integration with existing nanostructured devices [20,21].

A highly effective approach to discover new materials of novel properties relies on empirically validated chemical design principles, leveraging insights from existing materials to guide the exploration of new ones. Two prominent examples that illustrate this approach include the comprehensive exploration of MoS$_2$-type 2D transition-metal dichalcogenides (TMDCs) [22–26] and the discovery of the intrinsic quantum anomalous Hall (QAH) insulator MnBi$_2$Te$_4$ [27–31]. TMDCs exemplify the success of chemical design due to their vast configuration space arising from diverse chemical elements and structural phases. Their electronic properties are profoundly influenced by their structure: the 2H phase typically exhibits a semiconducting state with spin-valley locking [32], while the 1T phase often displays metallic behavior, regardless of its constituent elements. Manipulating the 1T phase through element substitution can lead to more intriguing outcomes. For instance, replacing certain elements may induce superconductivity through the melting of intrinsic charge density waves (e.g. TaS$_2$ [33,34] and TiSe$_2$ [35–37]) or cause electronic instability and Jahn–Teller distortion, leading to a transition to the 1T’ phase (or even Td phase) and hosting quantum spin Hall (QSH) effects (e.g. (Mo,W)Te$_2$ [24,38–41]). Furthermore, Janus structuring, which breaks inversion symmetry, introduces the Stark effect and Rashba spin splitting (e.g. MoSSe and WSSe [42,43]). The discovery of MnBi$_2$Te$_4$ highlights another example of chemical design. The pursuit of QAH insulators [44,45] initially involved extrinsically doping magnetic ions (Cr or V) into the QSH insulator (Sb,Bi)$_2$Se$_3$ [46,47]. Later advancements led to the intrinsic formation of MnBi$_2$Te$_4$ by depositing MnTe onto the topological insulator Bi$_2$Te$_3$ [27–31]. This chemical design approach also extends to many other material classes and novel properties, such as SnTe- and Sr$_3$PbO-type topological crystalline insulators [24,48–52], and WTe$_2$- and TaIrTe$_4$-type QSH insulators [39,53–56].

In this work, inspired by the V$_2$(Se,Te)$_2$O structure and its symmetry-preserving variants (both modified and Janus structured), along with the potential for valence-adaptable element substitution at all atomic sites, we propose systematic chemical design principles for monolayer altermagnets. In total, 2600 material candidates are rationally designed in four structural frameworks, including 880, 220, 1200 and 300 for M$_2$A$_2$B and M$_2$A$_2$B, and Janus-structured M$_2$AA$^{\prime }$B and M$_2$AA$^{\prime }$, respectively, where M is a transition metal, A/A$^{\prime }$ comes from either pnictogens, chalcogens or halogens and B is from chalcogens. The substitution elements are chosen with consideration of sustaining strong covalent bonding, compensating element valence and avoiding fractional valence. Our high-throughput first-principles calculations of all 2600 candidates cover a broad range of ground-state magnetic orderings, including AM, non-magnetic (NM), ferromagnetic (FM) and stripe-AFM, as well as electronic structures under ground-state orderings, namely semiconducting, metallic, half-metallic and Dirac-cone semi-metallic. In the calculations, plenty of altermagnets (131, 72, 308 and 101, respectively) are identified from candidates of all four structural frameworks. Surprisingly, some of them (10, 15, 33 and 21, respectively) can simultaneously host CSML Dirac cones near the Fermi surface, suggesting potential anisotropic and spin-selective carrier transport at ultra-high speed for future ultra-fast spintronics applications. Our designs and results provide guidance for future investigations of monolayer altermagnets and their distinct electronic structures and properties.

## RESULTS AND DISCUSSION

### Symmetry and design principles

**Figure 1.**
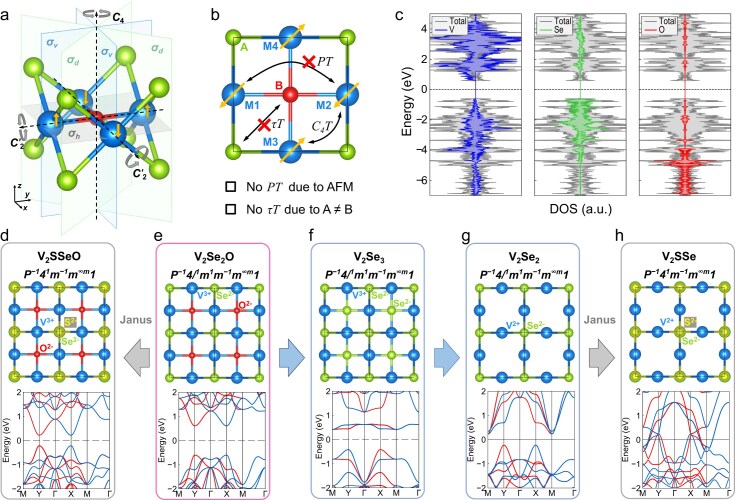
(a) Schematic crystal structure of V$_2$Se$_2$O monolayers. Blue, green and red spheres stand for V, Se and O. Its mirror and rotation symmetries are presented by transparent colored planes and combinations of black dashed and gray rotating arrows, with the respective notation shown beside. The Néel AFM-ordered spins on V are denoted by yellow arrows on blue spheres. (b) Structural framework M$_2$A$_2$B as the design basis. Atomic sites M, A and B resemble the in-plane locations of metal V, vertical-dimer Se-Se and single-ion O in V$_2$Se$_2$O, respectively; the symmetry requirements of altermagnetism are also marked. (c) Density of states (DOS) of V$_2$Se$_2$O (gray) and its projected ones onto V, Se and O ions (blue, green and red, respectively). (d–h) Top view of crystal structures (top panel) of Janus V$_2$SSeO, V$_2$Se$_2$O, V$_2$Se$_3$, V$_2$Se$_2$ and Janus V$_2$SSe, as well as their band structures (bottom panel) under Néel-AFM order. The in-between arrows denote the symmetry-preserving structural modification by site-B engineering (blue) and Janus structuring (gray) in our design principles. The valence of each constituent elements is explicitly marked in the crystal structures, and the top S and bottom Se ions in (c) and (g) are distinguished by small yellow and large green spheres. The spin space groups are also given above the respective schematic structures using the convention in [57].

The 2D crystal structure of layered V$_2$(Se,Te)$_2$O [6] belongs to point group $D_{4h}$ and space group $P4/mmm$, as depicted in Fig. 1a. Its square lattice is symmetric under rotations of four-fold principal $\mathcal {C}_4$ and two-fold non-principal $\mathcal {C}_2^{\prime }$, mirrors of horizontal $\sigma _h$, vertical $\sigma _v$ and dihedral $\sigma _d$, as well as multiple centers of inversion $\mathcal {P}$. The two spin-antiparallel magnetic sublattices (Fig. S1) can only be connected by the $\mathcal {C}_4$ rotation and $\sigma _d$ mirror symmetry, rather than the $\mathcal {P}$ or any half translations $\tau$, which leads to the spin-splitting band structures even without spin-orbit coupling and enforces the CSML. This is the direct consequence of identical spins at sites M1 and M2 (also, M3 and M4), and different non-metal chemical species at 2D Wykoff sites A and B (vertical-dimer Se and single-ion O), as shown in Fig. 1b. As long as the A $\ne$ B condition and checkboard-type AFM ordering withstand, the critical breaking of $\mathcal {P}\mathcal {T}$ and $\tau \mathcal {T}$ is preserved [58,59], and the atomic configurations of sites A and B can be changed by either modification or even site subtraction of site B, or Janus structure of two vertically stacked elements at site A (i.e. double A becoming Janus AA$^{\prime }$). It is worth mentioning that the vertical translations are absent in the 2D regime, and hence the spin-antiparallel magnetic sublattices can be connected only by $\mathcal {C}_4$ (or other even-fold operations) but not $\mathcal {C}_3$. Therefore, preserving the critical symmetry breaking and suitable magnetic structure is crucial to realize monolayer altermagnets.

Taking advantage of the critical symmetry and structural framework of synthesized V$_2$(Se,Te)$_2$O (M$_2$A$_2$B in Fig. 1b), we propose a systematic design approach for monolayer altermagnets through site modification and subtraction at the B site, and Janus structures at the A site. The effectiveness of this design approach is exemplified by monolayer V$_2$Se$_2$O and its derivatives, as shown in Fig. 1. Projected density of states (Fig. 1c) reveal that the majority of oxygen (O) electronic states at site B reside deep in the valence regime, with negligible influence on states near the Fermi level and thus minimal impact on electronic properties and magnetic orderings. Either replacing O with Se or removing it does not affect any symmetry and indeed maintains the semiconducting nature and AM order, as demonstrated in Fig. 1e–g. The in-gap flat bands (Fig. 1f) and the conduction-valley shift (Fig. 1g) can be attributed to enhanced correlation from the more active Se outer shells and reconstructed hybridization resulting from the removal of O $p_x/p_y$ orbitals. In addition, introducing a Janus structure, which still preserves the critical symmetry breaking, can introduce additional symmetry breaking and novel characteristics. To ensure stability, we substitute one of the vertically stacked Se atoms at site A with congeneric S, resulting in AM band structures with narrowing and even closing gaps in V$_2$SSeO and V$_2$SSe (Fig. 1c and h) due to the (vertical) symmetry-breaking-induced Stark effect. Inspired by these examples, we construct four structural frameworks, M$_2$A$_2$B and M$_2$A$_2$, and their Janus derivatives M$_2$AA$^{\prime }$B and M$_2$AA$^{\prime }$, for further design.

We next implement symmetry-allowed substitution of chemical elements at M, A/A$^{\prime }$ and B atomic sites within four design frameworks, with element selection aiming to maintain key chemical properties. The non-metal elements at site A and/or B are chosen from groups VA–VIIA (in the periodic table) to ensure strong covalent bonding analogous to that in V$_2$(Se,Te)$_2$O, while the metal elements at site M are chosen from transition metals (groups IIIB–IIB in the periodic table) because of their potential to host spin polarization and multiple oxidation states derived from partially filled *d* orbitals. We neglect very heavy elements and focus on ones within period 5 to match the common research interest. Consequently, the substituting elements encompass the first 20 transition metals (Sc–Cd) and the first four pnictogens (N–As), chalcogens (O–Te) and halogens (F–I), with metallic Sb being subtracted. To ensure the stability/metastability of the design candidates after substitution, the closed-shell electronic configuration (e.g. N$^{3-}$:[Ne], O$^{2-}$:[Ne] and F$^{1-}$:[Ne]) is required for non-metal elements, and the fractional or mixed valences are also avoided by restricting elements at site B to have even-integer valences (i.e. only chalcogens). Notably, the stability of transition metals is governed less by the closed *d* shell, but rather by ligand field effects and specific *d*-shell fillings (i.e. empty $d^0$ for early transition metals and half/full $d^5$/$d^{10}$ for late ones). Applying these rules and leveraging the multiple oxidation states enabled by transition-metal *d* shells, we design all feasible valence combinations (or ‘recipes’) for the substituting elements in the four aforementioned frameworks (Table 1). Therefore, a total of 2600 potential monolayer altermagnet candidates are generated by our design for high-throughput investigations, which include 880 for M$_2$A$_2$B, 220 for M$_2$A$_2$, 1200 for Janus M$_2$AA$^{\prime }$B and 300 for Janus M$_2$AA$^{\prime }$. As long as AM symmetry-preserving modifications and valence-adoptive substitutions can be applied, our design principles can be generalized to other 2D systems, such as A(BN)$_2$ [60], and even to 3D bulk ones.

**Table 1.** Possible valence recipes for substituting elements at sites M, A, A$^{\prime }$ and B in the four AM structural frameworks within the chemical design.

Design framework	M	A	A$^{\prime }$	B
M$_2$A$_2$B	+2	-1	–	-2
	+3	-2	–	-2
	+4	-3	–	-2
M$_2$A$_2$	+1	-1	–	–
	+2	-2	–	–
	+3	-3	–	–
M$_2$AA$^{\prime }$B	+2	-1	-1	-2
(Janus)	+3	-2	-2	-2
	+4	-3	-3	-2
M$_2$AA$^{\prime }$	+1	-1	-1	–
(Janus)	+2	-2	-2	–
	+3	-3	-3	–

### High-throughput calculations

The magnetic orderings and their corresponding electronic band structures for all 2600 candidate materials are evaluated and obtained using high-throughput first-principles calculations. Four magnetic orderings (or configurations) are considered in the calculations (Fig. S2), namely AM (equivalently, Néel AFM), stripe AFM (AFM for short), FM and NM. The lowest-energy one is identified as the ground state, with further distinction of the ground-state AFM order into stripe, zigzag-X and zigzag-Y shown in Fig. S3. Electronic band structures are calculated self-consistently based on these ground-state magnetic orderings.

**Figure 2.**
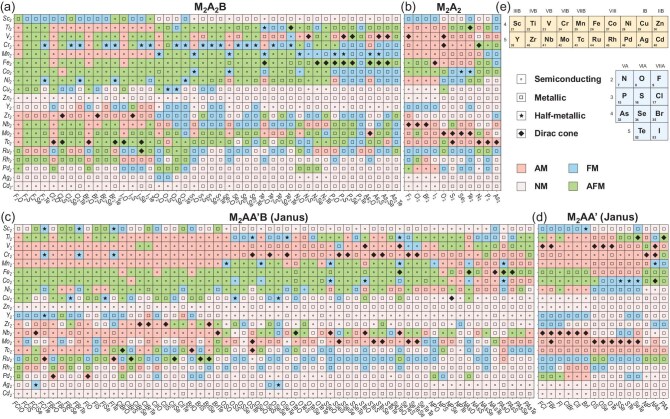
Ground-state magnetic orderings and electronic band structures of designed candidates from the frameworks (a) M$_2$A$_2$B, (b) M$_2$A$_2$, (c) Janus M$_2$AA$^{\prime }$B and (d) Janus M$_2$AA$^{\prime }$. The vertical and horizontal axes denote the metal and non-metal parts of the chemical formula. The horizontal altermagnetism-absent metal belts, the vertical dependence of the AM order on non-metal elements, and the strong relation between Dirac cones and the AM order are all clearly demonstrated. (e) Schematic periodic table showing the transition-metal and non-metal elements considered in our design.

**Figure 3.**
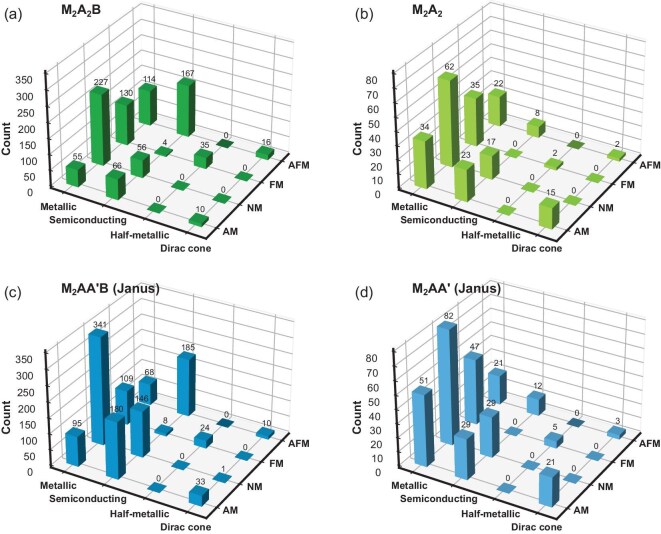
Statistical count of candidates with four different ground-state magnetic orderings and four distinct types of band structures for the material design frameworks (a) M$_2$A$_2$B, (b) M$_2$A$_2$, (c) Janus M$_2$AA$^{\prime }$B and (d) Janus M$_2$AA$^{\prime }$. The specific number for each pair of magnetic orderings and band-structure types is explicitly marked on top.

Our calculations identify 131 (14.9% of 880), 72 (32.7% of 220), 308 (25.7% of 1200) and 101 (33.7% of 300) potential monolayer altermagnets within the four respective design frameworks, yielding a total of 612, corresponding to 23.5% of the entire set of 2600. A diverse range of electronic properties, including semiconducting, metallic, half-metallic and, notably, Dirac-cone semimetallic, are also revealed. The finding that a quarter of the designed candidates are indeed the desired altermagnets underscores the rationality and effectiveness of our design principles, as well as contributes a substantial number of AM candidate materials for future experimental synthesis and investigation. More importantly, among these identified altermagnets, 10, 15, 33 and 21 candidates from the respective frameworks are also found to simultaneously host CSML Dirac-cone energy bands near the Fermi level. The ground-state magnetic orders and electronic band structures of all 2600 designed candidates are visually summarized in Fig. 2 using color fillings and centered symbols within mosaic squares (with corresponding statistical counts presented in Fig. 3), consistent with findings from the few examples reported previously [61,62].

Our results clearly demonstrate the dependence of AM ordering on the constituent metal and non-metal elements. In all four frameworks (Fig. 2), the emergence of the AM order shows a distinct dependence on the transition-metal constituents, leading to the formation of horizontal altermagnetism-absent belts of metals, regardless of the non-metal constituents. These belts span over six transition metals (Sc, Cu, Zn and Pd–Cd) in M$_2$A$_2$B (Fig. 2a) and seven (Pb replaced by Y and Ru) in M$_2$A$_2$ (Fig. 2b), while changing significantly in Janus M$_2$AA$^{\prime }$B and M$_2$AA$^{\prime }$ (Fig. 2c and d) to comprise four (Sc, Zn, Ag and Cd) and eight (Sc, Cu–Zr, Ru, Ag and Cd), respectively. Regarding the influence of non-metal constituents, M$_2$A$_2$B altermagnets (Fig. 2a) show a clear preference for either halogens occupying site A or oxygens occupying site B. Conversely, Janus M$_2$AA$^{\prime }$B altermagnets (Fig. 2c) exhibit much weaker dependence on non-metal constituents, although a slightly higher prevalence is observed when halogens or pnictogens occupy the A/A’ sites. In contrast, the formation of altermagnetism in M$_2$A$_2$ (Fig. 2b) and M$_2$AA$^{\prime }$ (Fig. 2d) appears to be largely insensitive to the A-site elements, with M $=$ Ni, Zr, Rh and Pd being exceptional cases that favor halogens at site A. These element-dependent trends can be attributed to the interplay between exchange interactions governed by the Goodenough–Kanamori–Anderson rules [63–65], and to the variations in *d*-shell electron filling arising from different chemical bonding environments and orbital hybridization.

The emergence of Dirac-cone band structures do have a strong dependence on the presence of AM order, as evidenced by Figs 2 and 3. Approximately 71.2% of candidates having Dirac-cone band structures are found to be AM; however, the dependence strength varies by design frameworks. The direct V$_2$Se$_2$O-analogous framework M$_2$A$_2$B has the weakest dependence, with only 38.5% of its Dirac-cone candidates being AM. In contrast, the modified and Janus-structured frameworks exhibit significantly enhanced dependence, namely 75.0% in M$_2$AA$^{\prime }$B, 87.5% in M$_2$AA$^{\prime }$ and peaking at 88.2% in M$_2$A$_2$, which adopts both B-site removal and Janus structuring. Such strong dependence likely stems from shared crystal-symmetry requirements for both Dirac-cone formation and altermagnetism, which are specifically the two symmetry-related sublattices composed of the same chemical species [66]. This suggests that the coexistence of Dirac cones and altermagnetism may be a frequent occurrence in other monolayer AM systems.

The Dirac-cone band structures possess linear, gapless band crossings near the Fermi level, which enables massless Dirac fermions and thus high-mobility carriers. When combined with the CSML, which occurs exclusively in altermagnets, the Dirac-cone-enabled ultra-fast transport exhibits spin selectivity and crystal anisotropy, and can be modulated by external strain more feasibly than by magnetic or electric fields, especially in nanostructures.

### Results of example candidates

**Figure 4.**
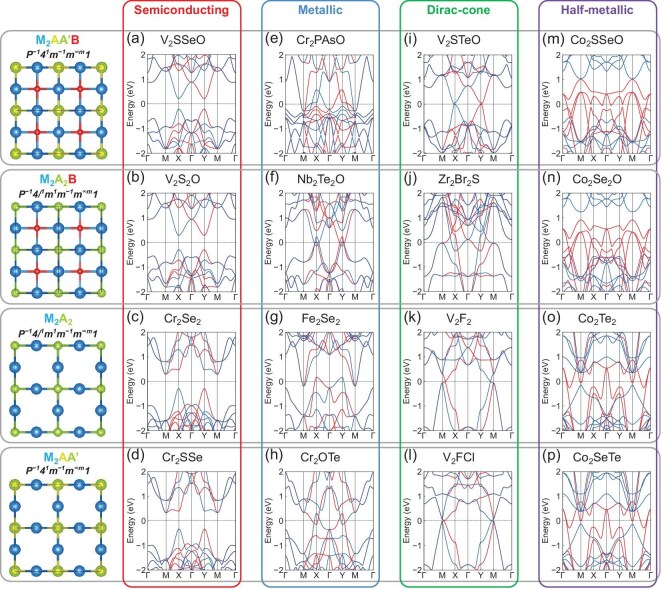
DFT-calculated spin-polarized band structures of selected AM candidates from the material design frameworks (from top to bottom) (a, e, i, m) Janus M$_2$AA$^{\prime }$B, (b, f, j, n) M$_2$A$_2$B, (c, g, k, o) M$_2$A$_2$ and (d, h, l, p) Janus M$_2$AA$^{\prime }$, hosting (from left to right) (a-d) semiconducting, (e-h) metallic, (i-l) Dirac-cone semimetallic and (m-p) half-metallic band features. The four types of band structures are marked at the top, while the top-view structures of the four material frameworks are shown on the left, with the colored letters in the chemical formula matching the respective atomic sites. AM ordering is the ground state for all semiconducting, metallic and Dirac-cone semimetallic candidates, whereas FM ordering occurs for the half-metallic ones. The spin space groups are also given above the respective schematic structures using the convention in ref. [57].

In Fig. 4, we selectively demonstrate examples of three primary spin-polarized electronic band structures under the AM order, namely semiconducting, metallic and Dirac-cone semimetallic, using specific material candidates. The evolution of band structures also reflects the influence of element substitutions to electronic structures. For semiconducting altermagnets (Fig. 4a–d), element substitutions primarily alter the magnitude of band gaps while largely preserving the overall shapes of band dispersions. For instance, in V$_2$S$_2$O, substituting Se with less electronegative S at site A (compared to V$_2$Se$_2$O in Fig. 1e) weakens the V-S covalent bonding, resulting in a narrowed band gap (Fig. 4b). Conversely, substituting V with Cr at site M in Cr$_2$Se$_2$ (compared to V$_2$Se$_2$ in Fig. 1g) enhances covalent bonding, as the *d*-shell filling of Cr$^{2+}$ ($3d^4$) is closer to half-filling than that of V$^{2+}$ ($3d^3$), leading to an enlarged band gap (Fig. 4c). Furthermore, the Janus structuring in V$_2$SSeO (Fig. 4a) and Cr$_2$SSe (Fig. 4d) only slightly modifies the gap magnitude due to the Stark effect arising from the Janus-induced structural asymmetry.

In contrast, for metallic altermagnets (Fig. 4e–h), element substitutions significantly modify the bonding states and orbital hybridization, causing shifts in both conduction and valence bands, which lead them to intersect the Fermi level. Specifically, in Nb$_2$Te$_2$O (Fig. 4f), the simultaneous substitutions at both sites M and A by chemically similar but more electropositive elements (V to Nb and Se to Te compared to V$_2$Se$_2$O) introduce more delocalized electrons, which lead to metallic states from intersecting valleys at the X point. Substituting Cr with Fe at site M in Fe$_2$Se$_2$ (compared to Cr$_2$Se$_2$) introduces electrons exceeding half filling of the *d* shell (Fe$^{2+}$ with $3d^6$), which weakens the covalent bonding and also closes the gap, as shown in Fig. 4g. The Janus structuring in metallic Cr$_2$PAsO (Fig. 4e) not only shifts conduction-valley momentum due to V-to-Cr substitution, but also gives rise to intersected, and more importantly, strongly hybridized and reconstructed bands (slightly below the Fermi level near the M point) due to the Stark effect combined with less electronegative substitution by pnictogens. In Janus Cr$_2$OTe (Fig. 4h), by contrast, the Janus-induced Stark effect primarily shifts both the conduction and valence bands to higher energies, causing them to intersect the Fermi level, compared with the case of Cr$_2$SSe.

The emergence of Dirac-cone band structures in altermagnets, as identified (Fig. 4i–l), involves not only substitution of more chemically active elements but also Stark-effect-inducing Janus structuring with congeneric elements. In both Zr$_2$Br$_2$S (Fig. 4j) and V$_2$F$_2$ (Fig. 4k), the substitution of original constituent elements (V, Cr, Se and O) with more chemically active ones results in significant interaction between the conduction and valence states at/near the Fermi level, leading to band-engineered formation of Dirac-cone linear dispersions (crossing points marked by black circles in Figs S3–S17). Differently, in V$_2$STeO (Fig. 4i), the Dirac cones are induced primarily by the Janus-induced Stark effect, causing the spin-polarized valleys (originally at the X/Y point of V$_2$Se$_2$O in Fig. 1e) to intersect precisely at a single point after the shift. The Dirac-cone band structures are preserved in V$_2$FCl after Janus structuring (Fig. 4l), which is expected given the chemical similarity between F and Cl, as well as the generally negligible influence of the Stark effect on metallic or semimetallic states.

**Figure 5.**
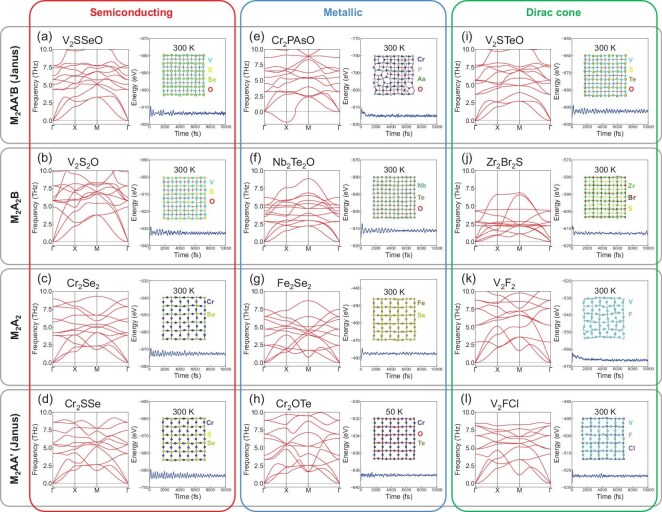
DFT-calculated phonon band structures (left) and AIMD simulations (right) for the same selected AM candidates from the four material frameworks (from top to bottom) (a, e, i) Janus M$_2$AA$^{\prime }$B, (b, f, j) M$_2$A$_2$B, (c, g, k) M$_2$A$_2$ and (d, h, l) Janus M$_2$AA$^{\prime }$, with (from left to right) (a-d) semiconducting, (e-h) metallic and (i-l) Dirac-cone semimetallic band features. The top-view crystal structures after AIMD simulations at 300 K (except 50 K for Cr$_2$OTe) after 10 ps are shown as insets for the different candidates, with colored letters on the right indicating the corresponding ions. Almost all candidates presented exhibit both kinetic and thermodynamic stability.

Nearly all altermagnets shown in Fig. 4 exhibit stability from both kinetic and thermodynamic perspective, as evidenced by first-principles phonon calculations and *ab initio* molecular dynamics (AIMD) simulations. As shown in Fig. 5, most candidates have no imaginary phonon modes and maintain atomic structures and energies at room temperature (about 300 K for over 10 ps), with only two exceptions: Cr$_2$PAsO and Cr$_2$OTe. Cr$_2$PAsO (Fig. 5e) shows large imaginary phonon modes, indicating kinetic instability despite seemingly stable AIMD results. Cr$_2$OTe (Fig. 5h), while having no imaginary phonon modes, demonstrates structural stability in AIMD simulations only at a low temperature (50 K). The confirmed stability of these altermagnets highlights their potential feasibility for future experimental synthesis and in-depth investigations.

In addition to band structures under the AM order, panels (m)–(p) of Fig. 4 also selectively present examples of spintronics-favored half-metallic structures under the FM order. The single-spin metallic character showcased by materials like Co$_2$Se$_2$O (Fig. 4n) and Co$_2$Te$_2$ (Fig. 4o) arises directly from band engineering. This involves not only weakened covalent bonding resulting from substitution with certain chemically active elements, but also altered *d*-shell filling due to the interplay between excess *d* electrons and FM crystal-field splitting. Notably, in contrast to the AM cases, subsequent Janus structuring exerts negligible influence on these half-metallic band structures (Fig. 4m and p). Such negligible influence can be attributed to the relative ineffectiveness of the intrinsic dipole field associated with the Stark effect in metallic systems. In the half-metallic phase, the band structure of only one spin channel is metallic, while that of the other is semiconducting or insulating, which enables complete spin polarization and spin conduction at/near the Fermi level and significantly reduces scattering from non-polarized electrons and energy dissipation. Unlike the normal FM phase, the half-metallic phase is robust at high temperatures [67] and also in low dimensions [68], which is beneficial for future nanostructured spintronics applications.

## CONCLUSIONS

In summary, this study establishes design principles for monolayer altermagnets utilizing symmetry-preserving structural engineering and valence-adaptive element substitution. Starting from the solely synthesized layered altermagnet V$_2$(Se,Te)$_2$O as the structure template, we derive four distinct structural frameworks: M$_2$A$_2$B, M$_2$A$_2$, M$_2$AA$^{\prime }$B and M$_2$AA$^{\prime }$. They are generated by either removing site B or Janus structuring at site A, while preserving the critical symmetry required by altermagnetism. On this basis, we construct 2600 candidates in total by substituting elements at all atomic sites, provided the valence-adaptive and closed-shell conditions are satisfied. The elements occupying sites M, A/A$^{\prime }$ and B are taken from the first 20 transition metals, the first four non-metal elements of groups VA–VIIA and the first four chalcogens, respectively. Further high-throughput first-principles calculations determine their ground-state magnetic orderings and electronic band structures, and identify 612 altermagnets representing approximately one-quarter of the designed candidates. Such highly predictive accuracy proves the rationality and effectiveness of our design principles in searching atomically thin altermagnets and implies potential generalization to other similar systems. Notably, 79 of these altermagnets simultaneously host CSML Dirac-cone band structures near the Fermi level. The coexistence of the AM order and CSML Dirac cones gives rise to anisotropic, spin-selective, ultra-fast carrier transport, thus holding promising for spintronics research and applications. Our designs and calculations not only provide plenty of highly realizable monolayer altermagnets and their diverse electronic properties to guide future investigations, but also establish generalizable principles for their rational design.

## METHODS

The high-throughput first-principles calculations in the chemical design are performed using density functional theory (DFT) as implemented in the Vienna *ab initio* Simulation Package (VASP) [69,70]. The exchange-correlation and ion-electron interactions are treated using the Perdew–Burke–Ernzerhof functional [71] and the projected-augmented wave method [72,73] within the generalized gradient approximation. The vacuum space between the periodic images along the *z* axis is no less than 12 Å to avoid unwanted interactions from neighboring van der Waals layers. Throughout all calculations, the convergence criteria for energy and force are less than $1\times 10^{-5}$ eV and $-0.01$ eV/Å, and the plane-wave energy cutoff is kept as 600 eV. The k-point sampling uses a Monkhorst–Pack scheme [74] with an accuracy of $0.06\pi /$Å and $0.02\pi /$Å for structural relaxations and self-consistent electronic calculations, respectively. To obtain reasonable electronic structures and magnetic orderings, the on-site Coulomb interaction is included by adding effective Hubbard U on each transition-metal element, following [75]. Phonon band structures are calculated using density functional perturbation theory [76] as implemented in VASP, with the aid of the PHONOPY package [77,78]. The pseudo-imaginary modes in a few candidates may arise from anharmonic higher-order phonons and are corrected by the Hiphive package [79,80]. AIMD simulations are also performed in VASP using a Nosé–Hoover thermostat [81–83] for no less than 10 ps.

## Supplementary Material

nwaf528_Supplemental_File
