# Supplementary material for: Chemical design of monolayer altermagnets
Source: Natl Sci Rev. 2025 Nov 22;13(2):nwaf528. doi: 10.1093/nsr/nwaf528 (PMC12831033; doi:10.1093/nsr/nwaf528)
Supplement: nwaf528_Supplemental_File [file nwaf528_supplemental_file.pdf]

Supplementary Data for
Chemical design of monolayer altermagnets

Runzhang Xu,[1,*] Yifan Gao,[1] and Junwei Liu[1,†]

[1] *Department of Physics, The Hong Kong University of Science and Technology, Clear Water Bay, Kowloon, Hong Kong, China*

Contents

1. Crystal symmetry-connected magnetic sublattices 2

2. Determination of magnetic orderings and anisotropy 2

3. Dynamically stable candidates from high-throughput calculations 4

4. Summary table of key properties for all 2600 candidates 5

5. Band structures from high-throughput calculations 64

This PDF file includes:

Supplementary Material 1 to 5.

Supplementary Figures S1 to S21.

Supplementary Tables S1 to S4.

* xurzliuj@ust.hk
† liuj@ust.hk

1. Crystal symmetry-connected magnetic sublattices

The Néel antiferromagnetic (AFM) ordered collinear spins on V ions in monolayer $V_2(Se,Te)_2O$ forms two spin-antiparallel magnetic sublattices (A and B denoted by red and yellow shaded squares), as depicted in Fig. S1. They can only be connected (or related) by crystal symmetry of four-fold rotation $\mathcal{C}_4$ ($\mathcal{C}_4\mathcal{T}$) rather than half translation τ ($\tau\mathcal{T}$), with $\mathcal{T}$ being the time-reversal symmetry. Such symmetry-connected magnetic sublattices break the combined symmetry of $\mathcal{PT}$ (with $\mathcal{P}$ being inversion), thus giving rise to altermagnetic spin splitting in momentum space under Néel-AFM order in real space, also termed as crystal-symmetry-paired spin-momentum locking (CSML).

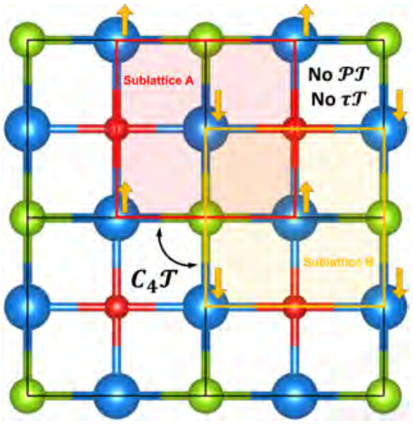

FIG. S1. Schematic of two spin-antiparallel magnetic sublattices in altermagnetic $V_2(Se,Te)_2O$ monolayers. Blue, green, and red spheres denote the V, Se(Te), and O ions. Yellow arrows mark the Néel-AFM-ordered collinear spins. Red and yellow shaded squares represent the two $\mathcal{C}_4\mathcal{T}$-related magnetic sublattices A and B.

2. Determination of magnetic orderings and anisotropy

Before band-structure calculations, the ground-state magnetic orderings of all 2600 design candidates are first evaluated by high-throughput first-principles methods. Here, six magnetic orderings are considered in the evaluation, which are altermagnetic (AM, or Néel-AFM), ferromagnetic (FM), non-magnetic (NM), AFM stripe, AFM zigzag-X and AFM zigzag-Y, as schematically shown in Fig. S2. For each candidate, the energy of four orderings is respectively calculated and compared, and the lowest-energy ordering is marked as the ground state. The determined magnetic orderings for all candidates are displayed in Fig. 2 of manuscript as color fillings with three AFM orders all classified as AFM for clear demonstration, and further distinguished in details in Fig. S3. Notably, some candidates have near-zero moments ($< 0.1\ \mu_B$) on magnetic ions even with lower energy in AM, FM, or AFM order, which may come from computational errors and are thus considered as NM. We also calculate the magnetic anisotropy by comparing the energies of spins parallels to [001], [100], and [110] directions and present the lowest-energy direction in Fig. S4.

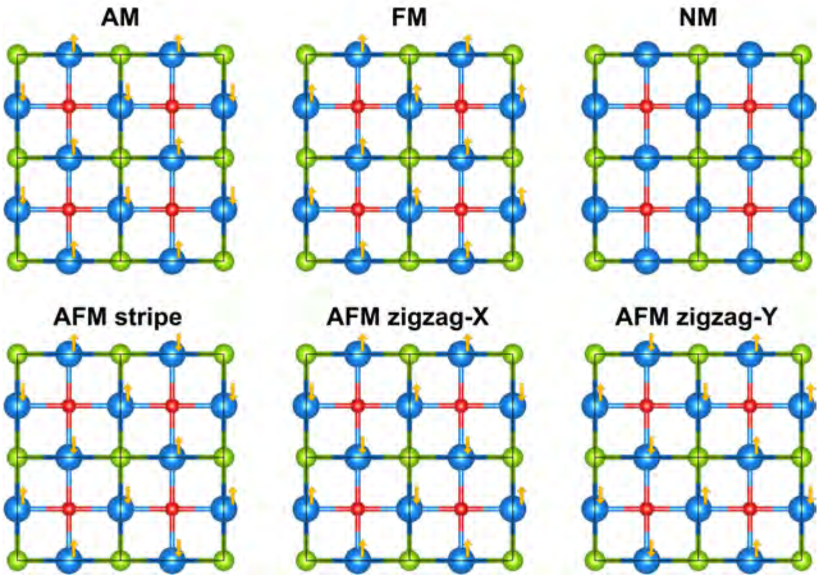

FIG. S2. Schematics of six magnetic orderings considered in high-throughput first-principles calculations. The collinear spins are presented by yellow arrows.

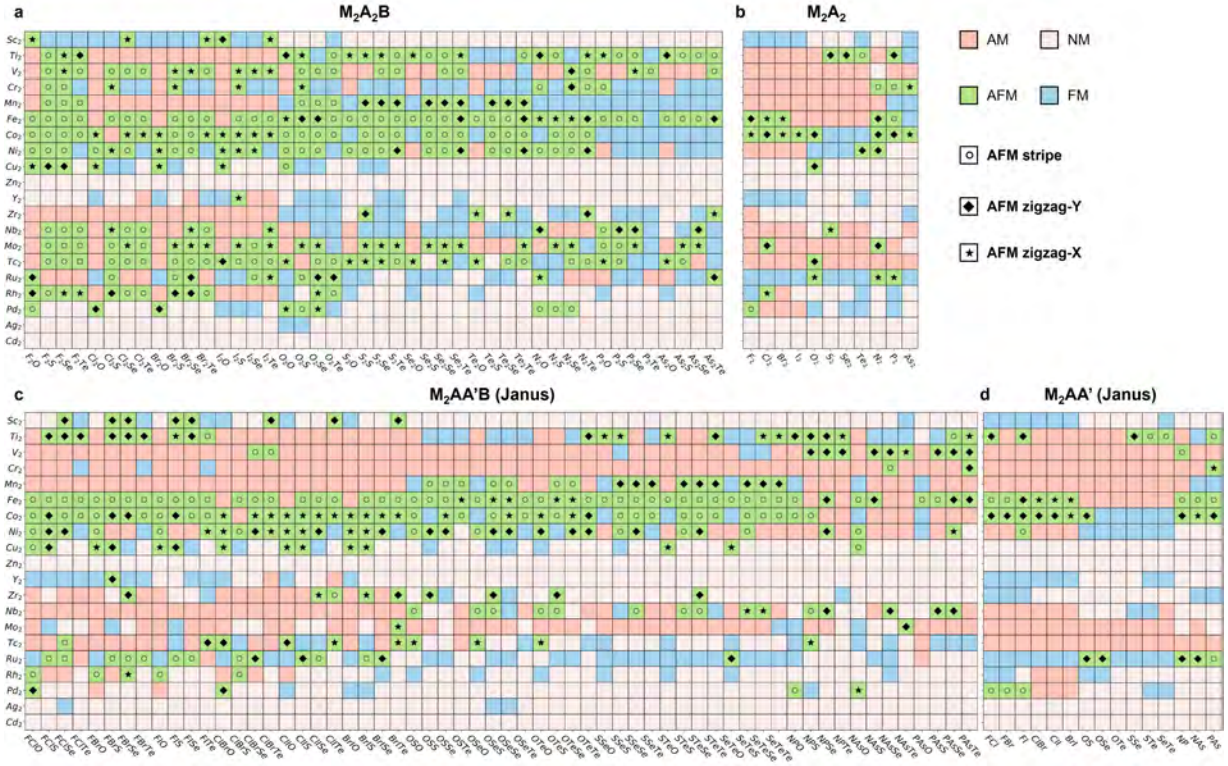

FIG. S3. Ground-state magnetic orderings (colored fillings) for all candidates and detailed AFM orders (centered symbols) for AFM candidates from structural framework (**a**) M_2A_2B, (**b**) M_2A_2, (**c**) Janus $M_2AA'B$, and (**d**) Janus M_2AA'. The vertical and horizontal axes denote the metal and non-metal parts of the chemical formula.

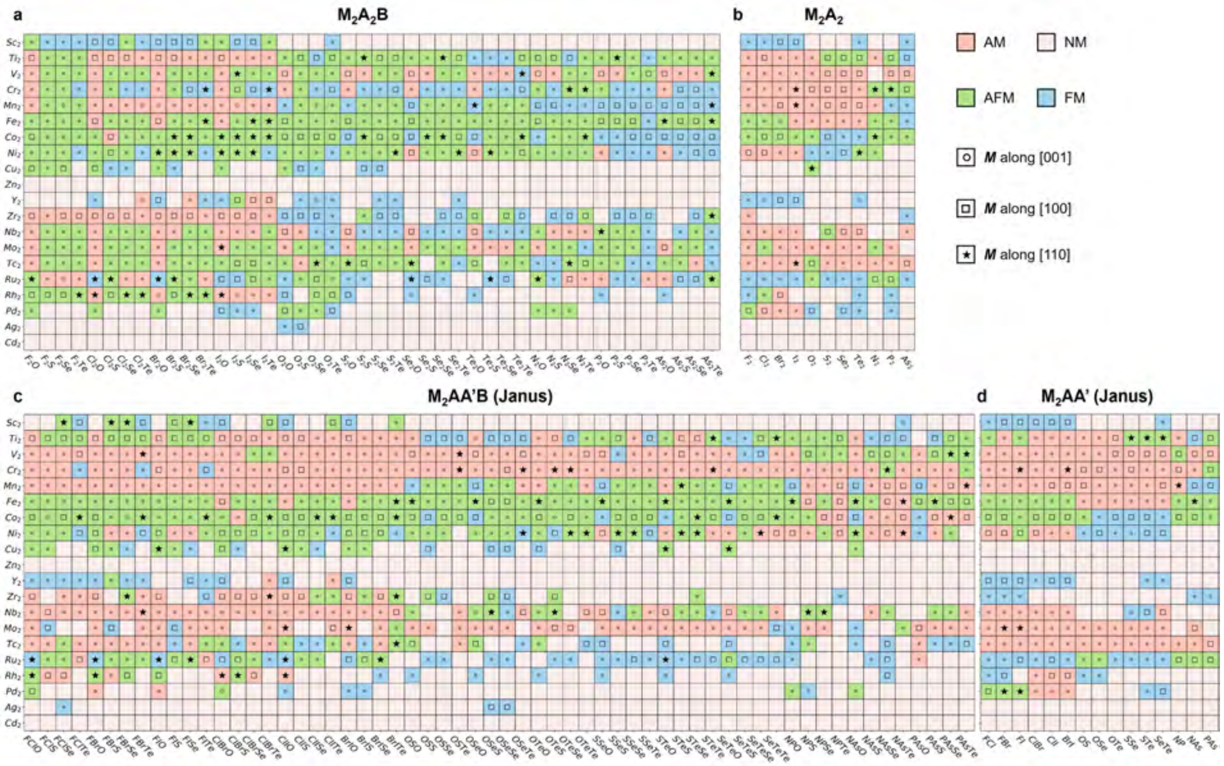

FIG. S4. Ground-state magnetic orderings (colored fillings) and magnetic anisotropy (centered symbols) for structural framework (**a**) M_2A_2B, (**b**) M_2A_2, (**c**) Janus $M_2AA'B$, and (**d**) Janus M_2AA', including three directions of [001], [100], and [110]. The vertical and horizontal axes denote the metal and non-metal parts of the chemical formula.

3. Dynamically stable candidates from high-throughput calculations

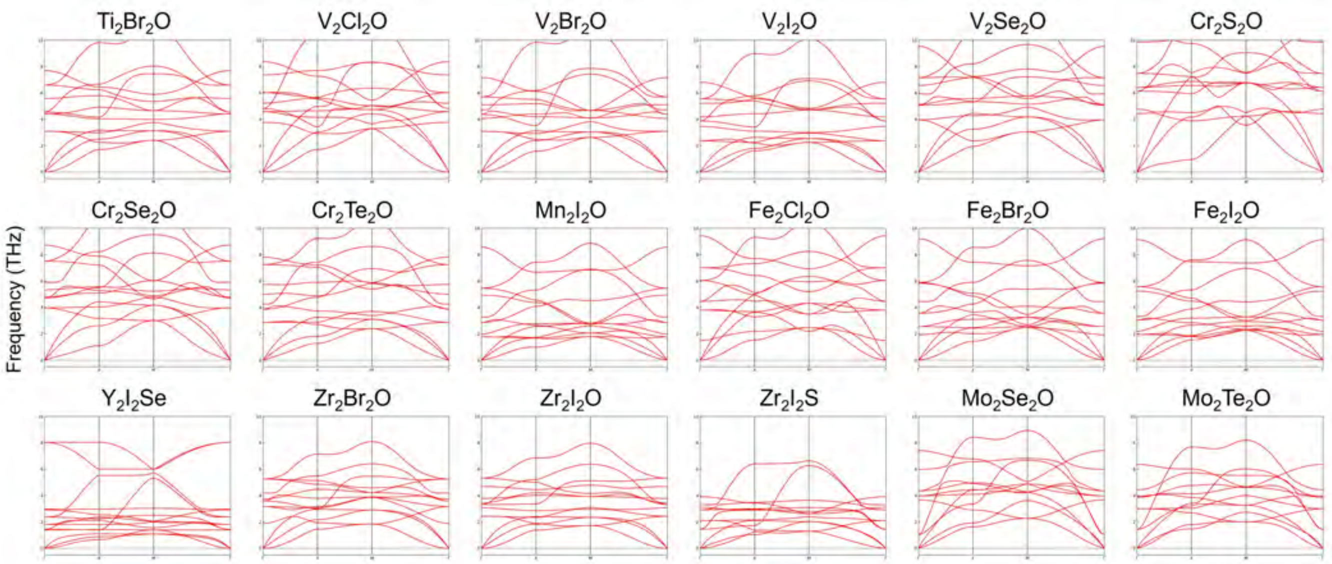

FIG. S5. Phonon band structures of selected stable altermagnets of M_2A_2B framework.

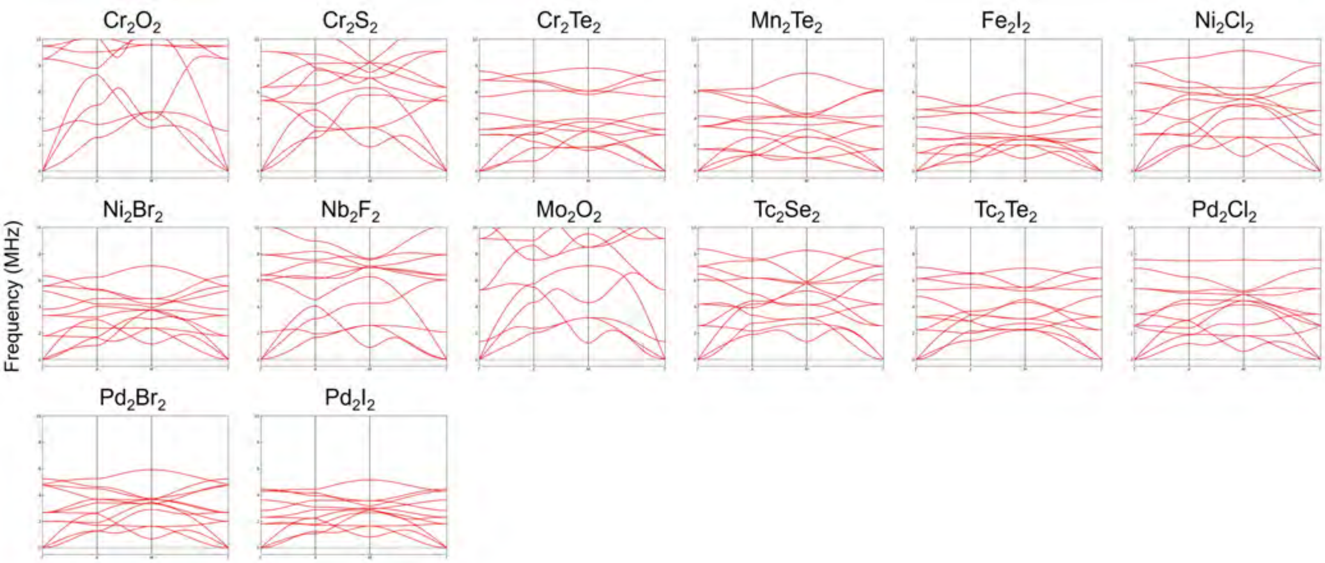

FIG. S6. Phonon band structures of selected stable altermagnets of M_2A_2 framework.

4. Summary table of key properties for all 2600 candidates

The key properties of all 2600 candidates in our high-throughput calculations, including lattice constant, magnetic orders, magnetic moments (Moments for short), band gaps, whether Dirac-cone altermagnets, locations of Dirac cones in momentum space (K-location for short), and magnetic anisotropy (MA for short), are summarized in Table S1-S4.

TABLE S1: Summary of key properties for all 880 candidates from the structural framework M_2A_2B.

Material	Lattice constant (Å)	Magnetic order	Moment (μ_B)	Band gap (eV)	Dirac-cone	K-location	MA
Sc_2F_2O	4.18	AFM zigzag-X	0.3	0.000	N	-	[001]
Sc_2F_2S	4.72	FM	0.4	0.000	N	-	[001]
Sc_2F_2Se	4.93	FM	0.5	0.000	N	-	[001]
Sc_2F_2Te	5.28	FM	0.7	0.000	N	-	[001]
Sc_2Cl_2O	4.30	FM	0.6	0.000	N	-	[100]
Sc_2Cl_2S	4.86	FM	0.4	0.000	N	-	[100]
Sc_2Cl_2Se	5.04	AFM zigzag-X	0.3	0.000	N	-	[001]
Sc_2Cl_2Te	5.39	FM	0.6	0.000	N	-	[001]
Sc_2Br_2O	4.31	FM	0.6	0.000	N	-	[100]
Sc_2Br_2S	4.91	FM	0.4	0.000	N	-	[100]
Sc_2Br_2Se	5.11	FM	0.5	0.000	N	-	[100]
Sc_2Br_2Te	5.43	AFM zigzag-X	0.6	0.341	N	-	[001]
Sc_2I_2O	4.30	AFM zigzag-Y	0.2	0.000	N	-	[001]
Sc_2I_2S	4.98	FM	0.5	0.000	N	-	[100]
Sc_2I_2Se	5.16	FM	0.5	0.000	N	-	[100]
Sc_2I_2Te	5.51	AFM zigzag-X	0.6	0.434	N	-	[001]
Sc_2O_2O	4.05	NM	0.0	0.000	N	-	-
Sc_2O_2S	4.59	NM	0.0	0.000	N	-	-
Sc_2O_2Se	4.77	NM	0.0	0.000	N	-	-
Sc_2O_2Te	5.07	FM	0.2	0.000	N	-	[001]
Sc_2S_2O	4.28	NM	0.0	0.417	N	-	-

Sc_2S_2S	4.79	NM	0.0	0.000	N	-	-
Sc_2S_2Se	4.95	NM	0.0	0.000	N	-	-
Sc_2S_2Te	5.23	NM	0.0	0.000	N	-	-
Sc_2Se_2O	4.33	NM	0.0	0.472	N	-	-
Sc_2Se_2S	4.85	NM	0.0	0.000	N	-	-
Sc_2Se_2Se	5.01	NM	0.0	0.000	N	-	-
Sc_2Se_2Te	5.29	NM	0.0	0.000	N	-	-
Sc_2Te_2O	4.40	NM	0.0	0.507	N	-	-
Sc_2Te_2S	4.94	NM	0.0	0.000	N	-	-
Sc_2Te_2Se	5.11	NM	0.0	0.000	N	-	-
Sc_2Te_2Te	5.39	NM	0.0	0.000	N	-	-
Sc_2N_2O	4.22	NM	0.0	0.236	N	-	-
Sc_2N_2S	4.71	NM	0.0	0.000	N	-	-
Sc_2N_2Se	4.88	NM	0.0	0.000	N	-	-
Sc_2N_2Te	5.20	NM	0.0	0.000	N	-	-
Sc_2P_2O	4.59	NM	0.0	0.526	N	-	-
Sc_2P_2S	4.99	NM	0.0	0.000	N	-	-
Sc_2P_2Se	5.11	NM	0.0	0.000	N	-	-
Sc_2P_2Te	5.36	NM	0.0	0.000	N	-	-
Sc_2As_2O	4.62	NM	0.0	0.723	N	-	-
Sc_2As_2S	5.04	NM	0.0	0.000	N	-	-
Sc_2As_2Se	5.17	NM	0.0	0.000	N	-	-
Sc_2As_2Te	5.41	NM	0.0	0.000	N	-	-
Ti_2F_2O	4.13	AM	1.6	1.113	N	-	[100]
Ti_2F_2S	4.72	AFM stripe	1.6	1.170	N	-	[001]
Ti_2F_2Se	4.87	AFM zigzag-X	1.6	1.231	N	-	[001]
Ti_2F_2Te	5.26	AFM zigzag-Y	1.7	1.374	N	-	[001]
Ti_2Cl_2O	4.24	AM	1.6	1.588	N	-	[100]
Ti_2Cl_2S	4.86	AM	1.6	1.405	N	-	[100]
Ti_2Cl_2Se	5.05	AM	1.7	1.574	N	-	[100]
Ti_2Cl_2Te	5.38	AM	1.7	1.715	N	-	[001]
Ti_2Br_2O	4.27	AM	1.5	1.535	N	-	[100]
Ti_2Br_2S	4.91	AM	1.6	1.407	N	-	[100]
Ti_2Br_2Se	5.15	AM	1.7	1.672	N	-	[001]
Ti_2Br_2Te	5.42	AM	1.7	1.714	N	-	[001]
Ti_2I_2O	4.30	AM	1.5	1.431	N	-	[100]
Ti_2I_2S	4.96	AM	1.6	1.344	N	-	[001]
Ti_2I_2Se	5.16	AM	1.6	1.526	N	-	[001]
Ti_2I_2Te	5.49	AM	1.7	1.679	N	-	[001]
Ti_2O_2O	3.89	AFM zigzag-Y	0.8	1.387	N	-	[001]
Ti_2O_2S	4.48	AFM zigzag-X	1.0	0.000	N	-	[100]
Ti_2O_2Se	4.66	FM	1.3	0.000	N	-	[100]
Ti_2O_2Te	5.20	AFM stripe	1.7	2.229	N	-	[100]
Ti_2S_2O	4.04	AFM zigzag-X	0.8	0.856	N	-	[001]
Ti_2S_2S	4.63	AFM zigzag-X	0.9	0.462	N	-	[110]

Ti_2S_2Se	4.81	AFM zigzag-X	0.9	0.334	N	-	[100]
Ti_2S_2Te	5.48	AFM stripe	1.7	2.260	N	-	[100]
Ti_2Se_2O	4.17	AFM zigzag-X	0.8	0.887	N	-	[001]
Ti_2Se_2S	4.69	AFM stripe	0.9	0.000	N	-	[001]
Ti_2Se_2Se	4.87	AFM stripe	1.0	0.000	N	-	[110]
Ti_2Se_2Te	5.21	AFM zigzag-X	1.3	0.321	N	-	[100]
Ti_2Te_2O	4.22	FM	1.0	0.000	N	-	[001]
Ti_2Te_2S	4.78	FM	1.0	0.000	N	-	[001]
Ti_2Te_2Se	4.96	FM	1.0	0.000	N	-	[001]
Ti_2Te_2Te	5.27	AFM stripe	1.3	0.000	Y	Γ-Y	[100]
Ti_2N_2O	4.02	AFM zigzag-Y	0.8	1.054	N	-	[100]
Ti_2N_2S	4.48	AFM stripe	0.9	0.000	N	-	[100]
Ti_2N_2Se	4.68	FM	1.0	0.000	N	-	[100]
Ti_2N_2Te	5.34	AFM zigzag-X	1.8	0.435	N	-	[001]
Ti_2P_2O	4.12	AFM zigzag-X	0.1	0.000	N	-	[001]
Ti_2P_2S	4.74	AFM stripe	0.9	0.000	N	-	[110]
Ti_2P_2Se	4.89	AFM stripe	1.0	0.000	N	-	[001]
Ti_2P_2Te	5.15	FM	1.0	0.000	N	-	[001]
Ti_2As_2O	4.13	AFM zigzag-Y	0.1	0.000	N	-	[001]
Ti_2As_2S	4.81	AFM stripe	0.9	0.000	N	-	[001]
Ti_2As_2Se	4.96	AFM stripe	1.0	0.000	N	-	[001]
Ti_2As_2Te	5.26	AFM stripe	1.1	0.000	N	-	[001]
V_2F_2O	4.04	AM	2.6	1.114	N	-	[001]
V_2F_2S	4.61	AFM stripe	2.6	1.510	N	-	[001]
V_2F_2Se	4.79	AFM zigzag-X	2.7	1.342	N	-	[001]
V_2F_2Te	5.07	AFM stripe	2.7	1.212	N	-	[001]
V_2Cl_2O	4.14	AM	2.6	1.748	N	-	[001]
V_2Cl_2S	4.73	AFM stripe	2.6	1.627	N	-	[001]
V_2Cl_2Se	4.92	AFM stripe	2.7	1.562	N	-	[001]
V_2Cl_2Te	5.24	AFM stripe	2.7	1.287	N	-	[001]
V_2Br_2O	4.18	AM	2.6	1.666	N	-	[001]
V_2Br_2S	4.77	AFM zigzag-X	2.7	1.528	N	-	[001]
V_2Br_2Se	4.96	AFM zigzag-X	2.7	1.415	N	-	[001]
V_2Br_2Te	5.28	AFM stripe	2.7	1.303	N	-	[001]
V_2I_2O	4.22	AM	2.6	1.505	N	-	[001]
V_2I_2S	4.83	AFM zigzag-X	2.6	1.475	N	-	[110]
V_2I_2Se	5.03	AFM zigzag-X	2.7	1.364	N	-	[001]
V_2I_2Te	5.33	AFM zigzag-X	2.7	1.095	N	-	[001]
V_2O_2O	3.81	AM	1.8	0.581	N	-	[100]
V_2O_2S	4.64	AFM stripe	2.6	1.669	N	-	[001]
V_2O_2Se	4.81	AFM stripe	2.7	1.588	N	-	[001]
V_2O_2Te	5.09	AFM stripe	2.7	1.366	N	-	[001]
V_2S_2O	3.98	AM	1.8	0.584	N	-	[100]
V_2S_2S	4.60	AM	2.3	0.000	N	-	[001]
V_2S_2Se	5.05	AFM stripe	2.7	1.015	N	-	[001]

V_2S_2Te	5.32	AFM stripe	2.7	1.261	N	-	[001]
V_2Se_2O	4.03	AM	1.9	0.305	N	-	[100]
V_2Se_2S	4.59	AM	2.1	0.000	N	-	[001]
V_2Se_2Se	5.12	AFM stripe	2.7	0.510	N	-	[001]
V_2Se_2Te	5.38	AFM stripe	2.7	0.822	N	-	[001]
V_2Te_2O	4.08	AM	1.9	0.000	N	-	[001]
V_2Te_2S	4.69	AM	2.2	0.000	N	-	[001]
V_2Te_2Se	4.95	AM	2.4	0.000	N	-	[001]
V_2Te_2Te	5.47	FM	2.7	0.100	N	-	[110]
V_2N_2O	3.90	AM	1.8	0.644	N	-	[100]
V_2N_2S	4.60	AM	2.5	0.000	Y	Γ-X, Γ-Y	[001]
V_2N_2Se	4.85	AFM zigzag-Y	2.8	0.000	N	-	[001]
V_2N_2Te	5.38	AFM stripe	3.1	0.306	N	-	[001]
V_2P_2O	4.29	AM	1.9	0.000	N	-	[100]
V_2P_2S	4.70	AM	2.1	0.000	Y	M-X, M-Y	[001]
V_2P_2Se	4.85	AFM zigzag-X	2.2	0.000	Y	Γ-Y	[001]
V_2P_2Te	5.18	AFM stripe	2.6	0.000	N	-	[100]
V_2As_2O	4.21	AM	1.9	0.000	N	-	[100]
V_2As_2S	4.74	AM	2.1	0.000	Y	M-X, M-Y	[001]
V_2As_2Se	4.90	AM	2.2	0.000	Y	M-X, M-Y	[001]
V_2As_2Te	5.18	AFM stripe	2.3	0.000	N	-	[110]
Cr_2F_2O	4.00	AM	3.7	1.078	N	-	[001]
Cr_2F_2S	4.55	AFM stripe	3.7	0.734	N	-	[001]
Cr_2F_2Se	4.73	AFM stripe	3.8	0.620	N	-	[001]
Cr_2F_2Te	5.07	FM	3.9	0.000	N	-	[001]
Cr_2Cl_2O	4.07	AM	3.6	1.039	N	-	[001]
Cr_2Cl_2S	4.68	AFM zigzag-X	3.7	0.592	N	-	[001]
Cr_2Cl_2Se	4.87	FM	3.8	0.000	N	-	[001]
Cr_2Cl_2Te	5.18	FM	3.9	0.000	N	-	[001]
Cr_2Br_2O	4.09	AM	3.6	0.949	N	-	[001]
Cr_2Br_2S	4.72	AFM zigzag-X	3.7	0.499	N	-	[001]
Cr_2Br_2Se	4.91	FM	3.8	0.000	N	-	[100]
Cr_2Br_2Te	5.22	FM	3.9	0.000	N	-	[110]
Cr_2I_2O	4.14	AM	3.6	0.612	N	-	[001]
Cr_2I_2S	4.76	AFM zigzag-X	3.7	0.352	N	-	[100]
Cr_2I_2Se	4.96	FM	3.8	0.000	N	-	[100]
Cr_2I_2Te	5.29	FM	3.9	0.000	N	-	[110]
Cr_2O_2O	3.76	AM	3.0	1.064	N	-	[001]
Cr_2O_2S	4.60	AFM zigzag-X	3.7	0.000	N	-	[001]
Cr_2O_2Se	4.78	FM	3.9	0.000	N	-	[001]
Cr_2O_2Te	5.08	FM	4.0	0.000	N	-	[100]
Cr_2S_2O	3.94	AM	3.0	1.117	N	-	[001]
Cr_2S_2S	4.54	FM	3.4	0.000	N	-	[001]
Cr_2S_2Se	4.97	FM	3.9	0.000	N	-	[001]
Cr_2S_2Te	5.27	FM	3.9	0.000	N	-	[100]

Cr_2Se_2O	3.99	AM	3.0	0.816	N	-	[001]
Cr_2Se_2S	4.59	FM	3.5	0.000	N	-	[001]
Cr_2Se_2Se	5.03	FM	3.8	0.000	N	-	[001]
Cr_2Se_2Te	5.33	FM	3.9	0.000	N	-	[001]
Cr_2Te_2O	4.07	AM	3.1	0.116	N	-	[001]
Cr_2Te_2S	4.68	FM	3.5	0.000	N	-	[001]
Cr_2Te_2Se	5.00	FM	3.6	0.000	N	-	[001]
Cr_2Te_2Te	5.40	FM	3.9	0.000	N	-	[100]
Cr_2N_2O	3.98	AFM stripe	3.1	0.884	N	-	[001]
Cr_2N_2S	4.55	FM	3.6	0.000	N	-	[001]
Cr_2N_2Se	5.10	AFM zigzag-Y	4.2	0.229	N	-	[110]
Cr_2N_2Te	5.54	AFM stripe	4.3	0.748	N	-	[110]
Cr_2P_2O	4.22	AFM stripe	3.1	0.600	N	-	[001]
Cr_2P_2S	4.69	FM	3.4	0.000	N	-	[001]
Cr_2P_2Se	4.85	FM	3.5	0.000	N	-	[001]
Cr_2P_2Te	5.15	FM	3.7	0.000	N	-	[001]
Cr_2As_2O	4.16	AM	3.1	0.000	N	-	[001]
Cr_2As_2S	4.74	FM	3.4	0.000	N	-	[100]
Cr_2As_2Se	4.90	FM	3.5	0.000	N	-	[100]
Cr_2As_2Te	5.19	FM	3.6	0.000	N	-	[001]
Mn_2F_2O	4.06	AM	4.6	2.376	N	-	[001]
Mn_2F_2S	4.58	AFM stripe	4.6	3.489	N	-	[001]
Mn_2F_2Se	4.76	AFM stripe	4.6	3.245	N	-	[001]
Mn_2F_2Te	5.09	AFM stripe	4.6	2.321	N	-	[001]
Mn_2Cl_2O	4.18	AM	4.6	3.128	N	-	[001]
Mn_2Cl_2S	4.74	AM	4.6	2.517	N	-	[001]
Mn_2Cl_2Se	4.93	AM	4.6	2.213	N	-	[001]
Mn_2Cl_2Te	5.23	AM	4.6	1.436	N	-	[001]
Mn_2Br_2O	4.21	AM	4.6	2.992	N	-	[001]
Mn_2Br_2S	4.80	AM	4.6	2.329	N	-	[001]
Mn_2Br_2Se	4.97	AM	4.6	1.983	N	-	[001]
Mn_2Br_2Te	5.28	AM	4.6	1.203	N	-	[001]
Mn_2I_2O	4.26	AM	4.6	2.552	N	-	[001]
Mn_2I_2S	4.85	AM	4.6	2.092	N	-	[001]
Mn_2I_2Se	5.05	AM	4.6	1.685	N	-	[001]
Mn_2I_2Te	5.35	AM	4.6	0.847	N	-	[001]
Mn_2O_2O	3.91	FM	4.3	0.000	N	-	[001]
Mn_2O_2S	4.67	AFM stripe	4.6	2.760	N	-	[001]
Mn_2O_2Se	4.82	AFM stripe	4.6	2.297	N	-	[001]
Mn_2O_2Te	5.07	AFM stripe	4.6	1.528	N	-	[001]
Mn_2S_2O	4.05	FM	4.2	0.000	N	-	[001]
Mn_2S_2S	4.95	AFM zigzag-Y	4.5	2.023	N	-	[001]
Mn_2S_2Se	5.09	AFM zigzag-Y	4.5	1.581	N	-	[001]
Mn_2S_2Te	5.35	AFM zigzag-Y	4.6	0.788	N	-	[001]
Mn_2Se_2O	4.11	FM	4.3	0.000	N	-	[100]

Mn_2Se_2S	5.03	AFM zigzag-Y	4.5	1.435	N	-	[001]
Mn_2Se_2Se	5.17	AFM zigzag-Y	4.5	1.345	N	-	[001]
Mn_2Se_2Te	5.42	AFM zigzag-Y	4.5	0.600	N	-	[001]
Mn_2Te_2O	4.10	FM	4.3	0.000	N	-	[110]
Mn_2Te_2S	5.13	AFM zigzag-Y	4.5	0.804	N	-	[001]
Mn_2Te_2Se	5.29	AFM zigzag-Y	4.5	0.777	N	-	[001]
Mn_2Te_2Te	5.54	AFM zigzag-Y	4.5	0.120	N	-	[001]
Mn_2N_2O	4.18	FM	4.4	0.000	N	-	[100]
Mn_2N_2S	4.71	FM	4.5	0.194	N	-	[100]
Mn_2N_2Se	4.87	FM	4.5	0.128	N	-	[001]
Mn_2N_2Te	5.19	FM	4.5	0.000	N	-	[001]
Mn_2P_2O	4.38	FM	4.2	0.000	N	-	[100]
Mn_2P_2S	4.85	FM	4.4	0.000	N	-	[100]
Mn_2P_2Se	5.01	FM	4.4	0.000	N	-	[100]
Mn_2P_2Te	5.31	FM	4.5	0.000	N	-	[100]
Mn_2As_2O	4.42	FM	4.3	0.000	N	-	[100]
Mn_2As_2S	4.90	FM	4.4	0.000	N	-	[100]
Mn_2As_2Se	5.06	FM	4.4	0.000	N	-	[100]
Mn_2As_2Te	5.33	FM	4.5	0.000	N	-	[110]
Fe_2F_2O	3.96	AFM stripe	3.7	2.409	N	-	[001]
Fe_2F_2S	4.47	AFM stripe	3.7	2.750	N	-	[001]
Fe_2F_2Se	4.65	AFM stripe	3.7	2.648	N	-	[001]
Fe_2F_2Te	4.95	AFM stripe	3.7	1.760	N	-	[001]
Fe_2Cl_2O	4.03	AM	3.7	2.981	N	-	[100]
Fe_2Cl_2S	4.51	AFM stripe	3.6	2.574	N	-	[001]
Fe_2Cl_2Se	4.70	AFM stripe	3.6	2.434	N	-	[001]
Fe_2Cl_2Te	5.12	AFM stripe	3.7	1.648	N	-	[001]
Fe_2Br_2O	4.07	AM	3.7	2.817	N	-	[100]
Fe_2Br_2S	4.66	AFM stripe	3.6	2.478	N	-	[001]
Fe_2Br_2Se	4.85	AFM stripe	3.7	2.164	N	-	[001]
Fe_2Br_2Te	5.17	AFM stripe	3.6	1.341	N	-	[110]
Fe_2I_2O	4.12	AM	3.7	2.421	N	-	[001]
Fe_2I_2S	4.73	AFM stripe	3.6	2.142	N	-	[001]
Fe_2I_2Se	4.91	AFM stripe	3.6	1.689	N	-	[110]
Fe_2I_2Te	5.21	AFM stripe	3.6	0.897	N	-	[110]
Fe_2O_2O	3.81	AFM zigzag-X	4.0	0.453	N	-	[100]
Fe_2O_2S	4.56	AFM zigzag-Y	3.7	2.025	N	-	[001]
Fe_2O_2Se	4.72	AFM zigzag-Y	3.7	1.702	N	-	[001]
Fe_2O_2Te	4.99	AFM stripe	3.6	1.286	N	-	[001]
Fe_2S_2O	4.00	AFM stripe	3.9	0.142	N	-	[100]
Fe_2S_2S	4.84	AFM stripe	3.6	1.731	N	-	[001]
Fe_2S_2Se	4.97	AFM stripe	3.6	1.343	N	-	[001]
Fe_2S_2Te	5.21	AFM stripe	3.6	0.604	N	-	[001]
Fe_2Se_2O	4.05	AFM stripe	3.9	0.000	N	-	[100]
Fe_2Se_2S	4.92	AFM stripe	3.6	1.259	N	-	[001]

Fe_2Se_2Se	5.05	AFM stripe	3.6	1.119	N	-	[001]
Fe_2Se_2Te	5.28	AFM zigzag-Y	3.6	0.395	N	-	[001]
Fe_2Te_2O	4.10	AFM stripe	3.7	0.000	Y	Γ-X	[001]
Fe_2Te_2S	4.98	AFM stripe	3.5	0.464	N	-	[001]
Fe_2Te_2Se	5.14	AFM stripe	3.5	0.619	N	-	[001]
Fe_2Te_2Te	5.39	AFM zigzag-Y	3.6	0.000	N	-	[001]
Fe_2N_2O	4.06	AFM zigzag-X	3.6	0.000	N	-	[100]
Fe_2N_2S	4.65	AFM zigzag-X	3.3	0.000	N	-	[001]
Fe_2N_2Se	4.85	AFM zigzag-X	3.2	0.000	Y	Γ-Y	[001]
Fe_2N_2Te	5.16	AFM zigzag-Y	3.1	0.000	Y	Γ-Y	[001]
Fe_2P_2O	4.27	AFM stripe	3.7	0.000	Y	Γ-X	[100]
Fe_2P_2S	4.70	AFM stripe	3.5	0.000	Y	Γ-X	[100]
Fe_2P_2Se	4.90	AFM stripe	3.4	0.000	Y	Γ-X	[100]
Fe_2P_2Te	5.05	FM	3.4	0.000	N	-	[001]
Fe_2As_2O	4.28	AFM stripe	3.7	0.000	Y	Γ-X	[110]
Fe_2As_2S	4.78	AFM stripe	3.5	0.000	Y	Γ-X	[100]
Fe_2As_2Se	4.94	AFM stripe	3.5	0.000	Y	Γ-X	[100]
Fe_2As_2Te	5.19	AFM zigzag-Y	3.4	0.000	N	-	[110]
Co_2F_2O	3.87	AFM stripe	2.7	3.397	N	-	[100]
Co_2F_2S	4.39	AFM stripe	2.7	1.060	N	-	[001]
Co_2F_2Se	4.57	AFM stripe	2.7	0.814	N	-	[001]
Co_2F_2Te	4.88	AFM stripe	2.7	0.397	N	-	[001]
Co_2Cl_2O	3.98	AFM zigzag-X	2.7	2.922	N	-	[001]
Co_2Cl_2S	4.54	AM	2.7	1.312	N	-	[100]
Co_2Cl_2Se	4.71	AFM zigzag-X	2.6	1.109	N	-	[001]
Co_2Cl_2Te	5.00	AFM zigzag-X	2.6	0.647	N	-	[001]
Co_2Br_2O	4.02	AFM zigzag-X	2.7	2.895	N	-	[001]
Co_2Br_2S	4.58	AFM stripe	2.6	2.038	N	-	[110]
Co_2Br_2Se	4.76	AFM stripe	2.6	1.688	N	-	[110]
Co_2Br_2Te	5.04	AFM zigzag-X	2.6	0.547	N	-	[001]
Co_2I_2O	4.09	AFM zigzag-X	2.6	2.452	N	-	[110]
Co_2I_2S	4.64	AFM zigzag-X	2.6	1.787	N	-	[110]
Co_2I_2Se	4.83	AFM zigzag-X	2.6	1.451	N	-	[110]
Co_2I_2Te	5.12	AFM zigzag-X	2.5	0.767	N	-	[110]
Co_2O_2O	3.79	AFM stripe	2.8	0.000	N	-	[100]
Co_2O_2S	4.48	AFM stripe	2.6	1.923	N	-	[100]
Co_2O_2Se	4.65	AFM stripe	2.6	1.503	N	-	[100]
Co_2O_2Te	4.89	AFM stripe	2.6	0.993	N	-	[100]
Co_2S_2O	3.90	FM	2.3	0.000	N	-	[100]
Co_2S_2S	4.71	AFM stripe	2.5	1.542	N	-	[110]
Co_2S_2Se	4.86	AFM stripe	2.5	1.178	N	-	[100]
Co_2S_2Te	5.10	AFM stripe	2.5	0.460	N	-	[100]
Co_2Se_2O	3.97	FM	2.4	0.000	N	-	[100]
Co_2Se_2S	4.78	AFM stripe	2.5	1.123	N	-	[110]
Co_2Se_2Se	4.93	AFM stripe	2.5	0.982	N	-	[110]

Co_2Se_2Te	5.18	AFM stripe	2.5	0.287	N	-	[100]
Co_2Te_2O	3.90	FM	2.5	0.000	N	-	[100]
Co_2Te_2S	4.84	AFM stripe	2.4	0.489	N	-	[001]
Co_2Te_2Se	5.01	AFM stripe	2.4	0.526	N	-	[001]
Co_2Te_2Te	5.27	AFM stripe	2.4	0.000	N	-	[110]
Co_2N_2O	4.06	FM	2.5	0.173	N	-	[001]
Co_2N_2S	4.64	AFM stripe	2.1	1.096	N	-	[001]
Co_2N_2Se	4.86	AFM stripe	2.1	1.224	N	-	[001]
Co_2N_2Te	5.21	AFM stripe	2.0	1.427	N	-	[110]
Co_2P_2O	4.18	FM	2.0	0.000	N	-	[001]
Co_2P_2S	4.62	FM	2.0	0.000	N	-	[001]
Co_2P_2Se	4.80	FM	2.0	0.000	N	-	[100]
Co_2P_2Te	5.07	FM	1.9	0.000	N	-	[100]
Co_2As_2O	4.19	FM	2.2	0.000	N	-	[100]
Co_2As_2S	4.65	FM	2.0	0.000	N	-	[100]
Co_2As_2Se	4.82	FM	2.1	0.000	N	-	[100]
Co_2As_2Te	4.88	FM	1.9	0.000	N	-	[100]
Ni_2F_2O	3.80	AFM stripe	1.8	2.270	N	-	[001]
Ni_2F_2S	4.31	AFM stripe	1.7	0.565	N	-	[001]
Ni_2F_2Se	4.49	AFM stripe	1.6	0.162	N	-	[001]
Ni_2F_2Te	4.74	FM	1.4	0.000	N	-	[001]
Ni_2Cl_2O	3.91	AFM stripe	1.7	1.969	N	-	[001]
Ni_2Cl_2S	4.45	AFM zigzag-X	1.6	0.677	N	-	[100]
Ni_2Cl_2Se	4.64	AFM stripe	1.6	0.305	N	-	[001]
Ni_2Cl_2Te	4.88	FM	1.4	0.000	N	-	[001]
Ni_2Br_2O	3.95	AFM zigzag-X	1.7	1.873	N	-	[110]
Ni_2Br_2S	4.50	AFM stripe	1.5	0.670	N	-	[110]
Ni_2Br_2Se	4.68	AFM stripe	1.5	0.223	N	-	[110]
Ni_2Br_2Te	4.92	FM	1.3	0.000	N	-	[001]
Ni_2I_2O	4.03	AFM zigzag-X	1.6	1.603	N	-	[110]
Ni_2I_2S	4.56	AFM zigzag-X	1.5	0.588	N	-	[110]
Ni_2I_2Se	4.51	AFM zigzag-X	1.4	0.159	N	-	[110]
Ni_2I_2Te	4.78	FM	1.2	0.000	N	-	[001]
Ni_2O_2O	3.96	AFM stripe	1.7	2.097	N	-	[001]
Ni_2O_2S	4.41	AFM stripe	1.6	1.043	N	-	[001]
Ni_2O_2Se	4.58	AFM stripe	1.6	0.739	N	-	[001]
Ni_2O_2Te	4.84	FM	1.3	0.000	N	-	[001]
Ni_2S_2O	4.03	AFM stripe	1.3	0.442	N	-	[001]
Ni_2S_2S	4.61	AFM stripe	1.4	0.612	N	-	[001]
Ni_2S_2Se	4.55	AFM stripe	1.3	0.000	N	-	[001]
Ni_2S_2Te	4.79	AFM zigzag-Y	1.0	0.000	N	-	[110]
Ni_2Se_2O	3.90	AM	1.0	0.000	N	-	[100]
Ni_2Se_2S	4.62	AFM stripe	1.3	0.470	N	-	[001]
Ni_2Se_2Se	4.79	AFM stripe	1.3	0.207	N	-	[001]
Ni_2Se_2Te	5.08	AFM zigzag-Y	1.1	0.000	N	-	[110]

Ni_2Te_2O	3.84	AM	1.1	0.000	N	-	[100]
Ni_2Te_2S	4.67	AFM stripe	1.1	0.000	N	-	[110]
Ni_2Te_2Se	4.85	AFM stripe	1.1	0.000	N	-	[001]
Ni_2Te_2Te	4.89	AFM zigzag-Y	1.0	0.000	N	-	[100]
Ni_2N_2O	3.84	AFM stripe	1.0	0.000	N	-	[001]
Ni_2N_2S	4.59	AFM stripe	1.0	0.380	N	-	[001]
Ni_2N_2Se	4.55	AFM stripe	1.0	0.237	N	-	[001]
Ni_2N_2Te	5.17	AFM zigzag-Y	1.0	0.494	N	-	[001]
Ni_2P_2O	3.92	AM	0.7	0.000	N	-	[001]
Ni_2P_2S	4.50	FM	0.8	0.000	N	-	[001]
Ni_2P_2Se	4.49	FM	0.7	0.000	N	-	[001]
Ni_2P_2Te	4.72	FM	0.7	0.000	N	-	[001]
Ni_2As_2O	3.85	AM	0.6	0.000	N	-	[001]
Ni_2As_2S	4.36	FM	0.6	0.000	N	-	[001]
Ni_2As_2Se	4.72	FM	0.8	0.000	N	-	[100]
Ni_2As_2Te	4.76	FM	0.7	0.000	N	-	[100]
Cu_2F_2O	3.78	AFM zigzag-X	0.7	1.176	N	-	[100]
Cu_2F_2S	4.22	AFM zigzag-Y	0.3	0.000	N	-	[001]
Cu_2F_2Se	4.43	AFM zigzag-Y	0.1	0.000	N	-	[100]
Cu_2F_2Te	4.74	NM	0.0	0.000	N	-	-
Cu_2Cl_2O	3.88	AFM zigzag-X	0.6	1.100	N	-	[100]
Cu_2Cl_2S	4.37	FM	0.2	0.000	N	-	[001]
Cu_2Cl_2Se	4.57	FM	0.1	0.000	N	-	[001]
Cu_2Cl_2Te	4.87	NM	0.0	0.000	N	-	-
Cu_2Br_2O	3.92	AFM zigzag-X	0.5	0.813	N	-	[001]
Cu_2Br_2S	4.42	FM	0.1	0.000	N	-	[001]
Cu_2Br_2Se	4.63	NM	0.0	0.000	N	-	-
Cu_2Br_2Te	4.92	NM	0.0	0.000	N	-	-
Cu_2I_2O	3.92	AFM zigzag-X	0.4	0.000	N	-	[001]
Cu_2I_2S	4.49	NM	0.0	0.000	N	-	-
Cu_2I_2Se	4.71	NM	0.0	0.000	N	-	-
Cu_2I_2Te	5.00	NM	0.0	0.000	N	-	-
Cu_2O_2O	3.97	AFM stripe	0.7	0.160	N	-	[001]
Cu_2O_2S	4.47	FM	0.2	0.000	N	-	[100]
Cu_2O_2Se	4.66	FM	0.2	0.000	N	-	[001]
Cu_2O_2Te	5.02	NM	0.0	0.000	N	-	-
Cu_2S_2O	4.19	NM	0.0	0.000	N	-	-
Cu_2S_2S	4.62	FM	0.1	0.000	N	-	[100]
Cu_2S_2Se	4.78	FM	0.1	0.000	N	-	[100]
Cu_2S_2Te	5.03	NM	0.0	0.000	N	-	-
Cu_2Se_2O	3.99	NM	0.0	0.000	N	-	-
Cu_2Se_2S	4.69	NM	0.0	0.000	N	-	-
Cu_2Se_2Se	4.84	NM	0.0	0.000	N	-	-
Cu_2Se_2Te	5.07	NM	0.0	0.000	N	-	-
Cu_2Te_2O	3.66	NM	0.0	0.000	N	-	-

Cu_2Te_2S	4.79	NM	0.0	0.000	N	-	-
Cu_2Te_2Se	4.95	NM	0.0	0.000	N	-	-
Cu_2Te_2Te	5.17	NM	0.0	0.000	N	-	-
Cu_2N_2O	4.19	NM	0.0	0.000	N	-	-
Cu_2N_2S	4.70	NM	0.0	0.840	N	-	-
Cu_2N_2Se	4.90	NM	0.0	0.941	N	-	-
Cu_2N_2Te	5.12	NM	0.0	1.035	N	-	-
Cu_2P_2O	4.21	NM	0.0	0.000	N	-	-
Cu_2P_2S	4.64	NM	0.0	0.000	N	-	-
Cu_2P_2Se	4.79	NM	0.0	0.000	N	-	-
Cu_2P_2Te	5.03	NM	0.0	0.000	N	-	-
Cu_2As_2O	4.25	NM	0.0	0.000	N	-	-
Cu_2As_2S	4.64	NM	0.0	0.000	N	-	-
Cu_2As_2Se	4.81	NM	0.0	0.000	N	-	-
Cu_2As_2Te	5.04	NM	0.0	0.000	N	-	-
Zn_2F_2O	3.87	NM	0.0	2.840	N	-	-
Zn_2F_2S	4.36	NM	0.0	2.069	N	-	-
Zn_2F_2Se	4.55	NM	0.0	1.292	N	-	-
Zn_2F_2Te	4.83	NM	0.0	0.056	N	-	-
Zn_2Cl_2O	4.01	NM	0.0	3.394	N	-	-
Zn_2Cl_2S	4.54	NM	0.0	0.775	N	-	-
Zn_2Cl_2Se	4.73	NM	0.0	0.153	N	-	-
Zn_2Cl_2Te	5.04	NM	0.0	0.000	N	-	-
Zn_2Br_2O	4.05	NM	0.0	3.205	N	-	-
Zn_2Br_2S	4.61	NM	0.0	0.376	N	-	-
Zn_2Br_2Se	4.80	NM	0.0	0.000	N	-	-
Zn_2Br_2Te	5.10	NM	0.0	0.000	N	-	-
Zn_2I_2O	4.13	NM	0.0	2.283	N	-	-
Zn_2I_2S	4.69	NM	0.0	0.000	N	-	-
Zn_2I_2Se	4.90	NM	0.0	0.000	N	-	-
Zn_2I_2Te	5.20	NM	0.0	0.000	N	-	-
Zn_2O_2O	4.05	NM	0.0	1.997	N	-	-
Zn_2O_2S	4.46	NM	0.0	0.707	N	-	-
Zn_2O_2Se	4.62	NM	0.0	0.030	N	-	-
Zn_2O_2Te	4.92	NM	0.0	0.000	N	-	-
Zn_2S_2O	4.32	NM	0.0	0.986	N	-	-
Zn_2S_2S	4.77	NM	0.0	0.000	N	-	-
Zn_2S_2Se	4.92	NM	0.0	0.000	N	-	-
Zn_2S_2Te	5.18	NM	0.0	0.000	N	-	-
Zn_2Se_2O	4.37	NM	0.0	0.221	N	-	-
Zn_2Se_2S	4.86	NM	0.0	0.000	N	-	-
Zn_2Se_2Se	5.01	NM	0.0	0.000	N	-	-
Zn_2Se_2Te	5.26	NM	0.0	0.000	N	-	-
Zn_2Te_2O	3.94	NM	0.0	0.000	N	-	-
Zn_2Te_2S	4.99	NM	0.0	0.000	N	-	-

Zn_2Te_2Se	5.15	NM	0.0	0.000	N	-	-
Zn_2Te_2Te	5.39	NM	0.0	0.000	N	-	-
Zn_2N_2O	4.09	NM	0.0	0.000	N	-	-
Zn_2N_2S	5.15	NM	0.0	0.000	N	-	-
Zn_2N_2Se	5.57	NM	0.0	0.000	N	-	-
Zn_2N_2Te	5.59	NM	0.0	0.000	N	-	-
Zn_2P_2O	4.33	NM	0.0	0.000	N	-	-
Zn_2P_2S	4.81	NM	0.0	0.000	N	-	-
Zn_2P_2Se	4.97	NM	0.0	0.000	N	-	-
Zn_2P_2Te	5.23	NM	0.0	0.000	N	-	-
Zn_2As_2O	4.37	NM	0.0	0.000	N	-	-
Zn_2As_2S	4.85	NM	0.0	0.000	N	-	-
Zn_2As_2Se	5.02	NM	0.0	0.000	N	-	-
Zn_2As_2Te	5.28	NM	0.0	0.000	N	-	-
Y_2F_2O	4.60	NM	0.0	0.000	N	-	-
Y_2F_2S	5.08	NM	0.0	0.000	N	-	-
Y_2F_2Se	5.23	NM	0.0	0.000	N	-	-
Y_2F_2Te	5.46	NM	0.0	0.000	N	-	-
Y_2Cl_2O	4.74	FM	0.2	0.000	N	-	[001]
Y_2Cl_2S	5.27	NM	0.0	0.000	N	-	-
Y_2Cl_2Se	5.45	NM	0.0	0.000	N	-	-
Y_2Cl_2Te	5.75	AM	0.1	0.000	N	-	[001]
Y_2Br_2O	4.77	FM	0.4	0.000	N	-	[100]
Y_2Br_2S	5.32	NM	0.0	0.000	N	-	-
Y_2Br_2Se	5.50	AM	0.1	0.000	N	-	[001]
Y_2Br_2Te	5.80	FM	0.2	0.000	N	-	[001]
Y_2I_2O	4.67	FM	0.4	0.000	N	-	[001]
Y_2I_2S	5.37	AFM zigzag-X	0.1	0.000	N	-	[100]
Y_2I_2Se	5.56	AM	0.1	0.000	N	-	[100]
Y_2I_2Te	5.85	AM	0.2	0.000	N	-	[100]
Y_2O_2O	4.44	NM	0.0	0.513	N	-	-
Y_2O_2S	4.97	FM	0.2	0.000	N	-	[001]
Y_2O_2Se	5.12	FM	0.2	0.000	N	-	[001]
Y_2O_2Te	5.39	FM	0.3	0.000	N	-	[001]
Y_2S_2O	4.69	NM	0.0	1.069	N	-	-
Y_2S_2S	5.19	NM	0.0	0.000	N	-	-
Y_2S_2Se	5.34	FM	0.1	0.000	N	-	[001]
Y_2S_2Te	5.61	FM	0.1	0.000	N	-	[001]
Y_2Se_2O	4.75	NM	0.0	1.099	N	-	-
Y_2Se_2S	5.25	NM	0.0	0.000	N	-	-
Y_2Se_2Se	5.40	NM	0.0	0.000	N	-	-
Y_2Se_2Te	5.67	FM	0.1	0.000	N	-	[001]
Y_2Te_2O	4.83	NM	0.0	1.114	N	-	-
Y_2Te_2S	5.35	NM	0.0	0.092	N	-	-
Y_2Te_2Se	5.50	NM	0.0	0.000	N	-	-

Y_2Te_2Te	5.77	NM	0.0	0.000	N	-	-
Y_2N_2O	4.62	NM	0.0	0.877	N	-	-
Y_2N_2S	5.07	NM	0.0	0.000	N	-	-
Y_2N_2Se	5.21	NM	0.0	0.000	N	-	-
Y_2N_2Te	5.49	NM	0.0	0.000	N	-	-
Y_2P_2O	5.01	NM	0.0	1.066	N	-	-
Y_2P_2S	5.39	NM	0.0	0.061	N	-	-
Y_2P_2Se	5.51	NM	0.0	0.000	N	-	-
Y_2P_2Te	5.74	NM	0.0	0.000	N	-	-
Y_2As_2O	5.04	NM	0.0	1.241	N	-	-
Y_2As_2S	5.45	NM	0.0	0.260	N	-	-
Y_2As_2Se	5.57	NM	0.0	0.105	N	-	-
Y_2As_2Te	5.79	NM	0.0	0.000	N	-	-
Zr_2F_2O	4.39	AM	1.0	0.170	N	-	[100]
Zr_2F_2S	4.87	AM	1.0	0.000	N	-	[001]
Zr_2F_2Se	4.96	AM	1.0	0.000	N	-	[100]
Zr_2F_2Te	5.23	AM	1.1	0.000	N	-	[100]
Zr_2Cl_2O	4.47	AM	1.0	0.237	N	-	[100]
Zr_2Cl_2S	4.98	AM	1.0	0.000	Y	M-X, M-Y	[100]
Zr_2Cl_2Se	5.21	AM	1.1	0.000	N	-	[100]
Zr_2Cl_2Te	5.56	AM	1.2	0.000	N	-	[001]
Zr_2Br_2O	4.49	AM	0.9	0.152	N	-	[100]
Zr_2Br_2S	5.02	AM	1.0	0.000	Y	M-X, M-Y	[100]
Zr_2Br_2Se	5.25	AM	1.1	0.000	N	-	[100]
Zr_2Br_2Te	5.60	AM	1.2	0.000	N	-	[001]
Zr_2I_2O	4.50	AM	0.8	0.000	N	-	[100]
Zr_2I_2S	5.00	AM	0.9	0.000	Y	M-X, M-Y	[100]
Zr_2I_2Se	5.18	AM	1.0	0.000	N	-	[100]
Zr_2I_2Te	5.63	AM	1.2	0.000	N	-	[001]
Zr_2O_2O	4.18	FM	0.3	0.000	N	-	[100]
Zr_2O_2S	4.65	FM	0.3	0.000	N	-	[100]
Zr_2O_2Se	4.83	FM	0.4	0.000	N	-	[100]
Zr_2O_2Te	5.14	FM	0.7	0.000	N	-	[001]
Zr_2S_2O	4.36	NM	0.0	0.000	N	-	-
Zr_2S_2S	4.82	AFM zigzag-Y	0.1	0.000	N	-	[001]
Zr_2S_2Se	5.00	FM	0.4	0.000	N	-	[100]
Zr_2S_2Te	5.26	FM	0.6	0.000	N	-	[100]
Zr_2Se_2O	4.40	NM	0.0	0.000	N	-	-
Zr_2Se_2S	4.87	NM	0.0	0.000	N	-	-
Zr_2Se_2Se	5.04	FM	0.3	0.000	N	-	[100]
Zr_2Se_2Te	5.34	FM	0.6	0.000	N	-	[100]
Zr_2Te_2O	4.45	AFM zigzag-X	0.2	0.000	N	-	[100]
Zr_2Te_2S	4.95	NM	0.0	0.000	N	-	-
Zr_2Te_2Se	5.13	AFM zigzag-X	0.3	0.000	N	-	[100]
Zr_2Te_2Te	5.42	FM	0.6	0.000	N	-	[100]

Zr_2N_2O	4.22	NM	0.0	0.000	N	-	-
Zr_2N_2S	4.75	FM	0.3	0.000	N	-	[100]
Zr_2N_2Se	4.92	FM	0.3	0.000	N	-	[100]
Zr_2N_2Te	5.23	AFM zigzag-Y	0.6	0.000	N	-	[100]
Zr_2P_2O	4.44	NM	0.0	0.000	N	-	-
Zr_2P_2S	5.03	FM	0.4	0.000	N	-	[100]
Zr_2P_2Se	5.15	FM	0.4	0.000	N	-	[100]
Zr_2P_2Te	5.40	FM	0.5	0.000	N	-	[100]
Zr_2As_2O	4.46	NM	0.0	0.000	N	-	-
Zr_2As_2S	4.99	NM	0.0	0.000	N	-	-
Zr_2As_2Se	5.21	FM	0.4	0.000	N	-	[100]
Zr_2As_2Te	5.45	AFM zigzag-X	0.3	0.000	N	-	[110]
Nb_2F_2O	4.29	AM	2.2	0.073	N	-	[001]
Nb_2F_2S	4.80	AFM stripe	2.2	0.540	N	-	[001]
Nb_2F_2Se	4.98	AFM stripe	2.3	0.547	N	-	[001]
Nb_2F_2Te	5.06	AFM stripe	2.2	0.397	N	-	[001]
Nb_2Cl_2O	4.35	AM	2.1	0.938	N	-	[001]
Nb_2Cl_2S	4.89	AFM zigzag-X	2.1	0.644	N	-	[001]
Nb_2Cl_2Se	5.10	AFM stripe	2.3	0.976	N	-	[001]
Nb_2Cl_2Te	5.42	AFM stripe	2.3	0.981	N	-	[001]
Nb_2Br_2O	4.37	AM	2.0	0.735	N	-	[001]
Nb_2Br_2S	4.93	AM	2.1	0.500	N	-	[001]
Nb_2Br_2Se	5.13	AFM zigzag-X	2.2	1.019	N	-	[001]
Nb_2Br_2Te	5.45	AFM stripe	2.3	0.986	N	-	[001]
Nb_2I_2O	4.29	AM	1.8	0.061	N	-	[001]
Nb_2I_2S	4.96	AM	2.0	0.365	N	-	[001]
Nb_2I_2Se	5.17	AM	2.2	0.597	N	-	[001]
Nb_2I_2Te	5.49	AFM zigzag-X	2.3	0.907	N	-	[001]
Nb_2O_2O	4.06	AM	1.2	0.000	N	-	[100]
Nb_2O_2S	4.43	AM	1.3	0.000	N	-	[001]
Nb_2O_2Se	4.64	FM	1.5	0.000	N	-	[001]
Nb_2O_2Te	4.94	FM	1.7	0.000	N	-	[001]
Nb_2S_2O	4.20	AM	1.1	0.000	N	-	[100]
Nb_2S_2S	4.58	FM	1.2	0.000	N	-	[001]
Nb_2S_2Se	4.84	FM	1.4	0.000	N	-	[001]
Nb_2S_2Te	5.07	FM	1.5	0.000	N	-	[001]
Nb_2Se_2O	4.24	AM	1.1	0.000	N	-	[100]
Nb_2Se_2S	4.63	FM	1.2	0.000	N	-	[001]
Nb_2Se_2Se	4.81	FM	1.4	0.000	N	-	[001]
Nb_2Se_2Te	5.29	FM	1.7	0.000	N	-	[001]
Nb_2Te_2O	4.28	AM	1.1	0.000	N	-	[100]
Nb_2Te_2S	4.78	FM	1.3	0.000	N	-	[001]
Nb_2Te_2Se	4.97	FM	1.4	0.000	N	-	[001]
Nb_2Te_2Te	5.35	FM	1.6	0.000	N	-	[001]
Nb_2N_2O	4.18	AFM zigzag-Y	1.0	0.000	N	-	[001]

Nb_2N_2S	4.56	AM	1.2	0.000	N	-	[001]
Nb_2N_2Se	4.82	AM	1.5	0.000	N	-	[001]
Nb_2N_2Te	5.29	AM	2.1	0.000	N	-	[001]
Nb_2P_2O	4.18	AFM stripe	0.4	0.000	N	-	[110]
Nb_2P_2S	4.76	AFM zigzag-Y	1.1	0.000	N	-	[001]
Nb_2P_2Se	4.91	AFM zigzag-Y	1.3	0.000	N	-	[001]
Nb_2P_2Te	5.20	FM	1.4	0.000	N	-	[001]
Nb_2As_2O	4.18	NM	0.0	0.000	N	-	-
Nb_2As_2S	4.73	FM	1.2	0.000	N	-	[001]
Nb_2As_2Se	4.96	AFM zigzag-Y	1.4	0.000	N	-	[001]
Nb_2As_2Te	5.24	FM	1.5	0.000	N	-	[001]
Mo_2F_2O	4.25	AM	3.2	0.415	N	-	[001]
Mo_2F_2S	4.72	AFM stripe	3.2	0.188	N	-	[001]
Mo_2F_2Se	4.94	AFM stripe	3.2	0.173	N	-	[001]
Mo_2F_2Te	5.17	AFM stripe	3.3	0.000	N	-	[001]
Mo_2Cl_2O	4.27	AM	3.0	0.739	N	-	[001]
Mo_2Cl_2S	4.51	AFM stripe	2.5	0.000	N	-	[001]
Mo_2Cl_2Se	4.71	AFM zigzag-X	3.0	0.000	N	-	[001]
Mo_2Cl_2Te	5.02	AFM stripe	3.1	0.000	N	-	[001]
Mo_2Br_2O	4.27	AM	3.0	0.674	N	-	[001]
Mo_2Br_2S	4.53	AFM zigzag-X	2.0	0.000	N	-	[001]
Mo_2Br_2Se	4.74	AFM zigzag-X	2.9	0.000	N	-	[001]
Mo_2Br_2Te	5.03	AFM zigzag-X	3.1	0.000	N	-	[001]
Mo_2I_2O	4.22	AM	2.8	0.333	N	-	[110]
Mo_2I_2S	4.57	AFM zigzag-X	1.9	0.000	N	-	[001]
Mo_2I_2Se	4.77	AFM stripe	2.5	0.000	N	-	[001]
Mo_2I_2Te	5.05	AFM zigzag-X	3.0	0.000	N	-	[001]
Mo_2O_2O	4.00	AM	2.3	0.545	N	-	[001]
Mo_2O_2S	4.37	AFM zigzag-X	1.9	0.000	N	-	[001]
Mo_2O_2Se	4.67	AFM zigzag-X	2.5	0.000	N	-	[001]
Mo_2O_2Te	5.22	FM	3.4	0.000	N	-	[001]
Mo_2S_2O	4.14	AM	2.1	0.454	N	-	[001]
Mo_2S_2S	4.58	AFM zigzag-X	2.1	0.000	N	-	[001]
Mo_2S_2Se	4.79	AFM zigzag-X	2.4	0.000	N	-	[001]
Mo_2S_2Te	5.11	AFM zigzag-X	2.6	0.000	N	-	[001]
Mo_2Se_2O	4.17	AM	2.1	0.592	N	-	[001]
Mo_2Se_2S	4.64	AFM zigzag-X	2.2	0.000	N	-	[001]
Mo_2Se_2Se	4.84	AFM zigzag-X	2.4	0.000	N	-	[001]
Mo_2Se_2Te	4.93	AFM zigzag-X	2.3	0.000	N	-	[001]
Mo_2Te_2O	4.22	AM	2.1	0.247	N	-	[001]
Mo_2Te_2S	4.70	AM	2.2	0.000	N	-	[001]
Mo_2Te_2Se	4.92	AM	2.4	0.000	N	-	[001]
Mo_2Te_2Te	5.22	AFM zigzag-X	2.6	0.000	N	-	[001]
Mo_2N_2O	4.20	AM	2.4	0.034	N	-	[001]
Mo_2N_2S	4.64	AFM zigzag-X	2.5	0.000	N	-	[001]

Mo_2N_2Se	4.85	AFM zigzag-X	2.7	0.000	N	-	[001]
Mo_2N_2Te	5.31	FM	3.3	0.000	N	-	[001]
Mo_2P_2O	4.16	AFM stripe	1.5	0.000	N	-	[001]
Mo_2P_2S	4.81	AFM stripe	2.4	0.000	N	-	[001]
Mo_2P_2Se	4.97	AFM zigzag-X	2.5	0.000	N	-	[001]
Mo_2P_2Te	5.23	FM	2.8	0.000	N	-	[001]
Mo_2As_2O	4.12	AM	1.4	0.000	Y	Γ-X, Γ-Y	[100]
Mo_2As_2S	4.85	AFM zigzag-X	2.4	0.000	N	-	[001]
Mo_2As_2Se	5.02	AFM zigzag-X	2.6	0.000	N	-	[001]
Mo_2As_2Te	5.28	FM	2.8	0.000	N	-	[001]
Tc_2F_2O	4.27	AM	4.1	0.398	N	-	[001]
Tc_2F_2S	4.72	AFM stripe	3.9	0.227	N	-	[001]
Tc_2F_2Se	4.90	AFM stripe	4.0	0.412	N	-	[001]
Tc_2F_2Te	5.15	AFM stripe	4.0	0.546	N	-	[001]
Tc_2Cl_2O	4.28	AM	3.8	0.000	Y	Γ-X, Γ-Y	[001]
Tc_2Cl_2S	4.84	AFM stripe	3.8	0.000	Y	M-X	[001]
Tc_2Cl_2Se	5.06	AFM stripe	4.0	0.507	N	-	[001]
Tc_2Cl_2Te	5.36	AFM stripe	4.0	0.761	N	-	[001]
Tc_2Br_2O	4.26	AM	3.7	0.000	Y	Γ-X, Γ-Y	[001]
Tc_2Br_2S	4.47	AFM stripe	2.9	0.116	N	-	[001]
Tc_2Br_2Se	5.10	AFM stripe	3.9	0.356	N	-	[001]
Tc_2Br_2Te	4.97	AFM stripe	3.4	0.000	Y	Γ-X	[001]
Tc_2I_2O	4.15	AFM zigzag-Y	2.7	0.000	Y	M-X	[001]
Tc_2I_2S	4.50	AFM stripe	2.8	0.215	N	-	[100]
Tc_2I_2Se	5.13	AFM stripe	3.8	0.000	Y	M-X	[100]
Tc_2I_2Te	5.44	AFM stripe	3.9	0.414	N	-	[001]
Tc_2O_2O	3.83	AFM zigzag-X	1.1	0.000	N	-	[001]
Tc_2O_2S	4.33	AM	2.2	0.000	N	-	[001]
Tc_2O_2Se	5.02	AFM stripe	4.0	0.632	N	-	[110]
Tc_2O_2Te	5.23	AFM stripe	3.9	0.531	N	-	[001]
Tc_2S_2O	4.00	AFM zigzag-X	1.1	0.000	N	-	[110]
Tc_2S_2S	4.32	AFM zigzag-X	1.0	0.000	N	-	[100]
Tc_2S_2Se	4.52	AFM zigzag-X	1.6	0.000	N	-	[001]
Tc_2S_2Te	5.49	AFM stripe	3.9	0.636	N	-	[001]
Tc_2Se_2O	4.04	AFM zigzag-X	1.2	0.000	N	-	[110]
Tc_2Se_2S	4.36	NM	0.0	0.000	N	-	-
Tc_2Se_2Se	4.56	AFM zigzag-X	1.6	0.000	N	-	[001]
Tc_2Se_2Te	4.84	FM	1.8	0.000	N	-	[001]
Tc_2Te_2O	4.10	AFM zigzag-X	1.2	0.000	N	-	[100]
Tc_2Te_2S	4.42	NM	0.0	0.000	N	-	-
Tc_2Te_2Se	4.83	AFM stripe	2.4	0.000	N	-	[001]
Tc_2Te_2Te	5.12	AFM stripe	2.6	0.000	N	-	[001]
Tc_2N_2O	4.12	FM	2.5	0.000	N	-	[001]
Tc_2N_2S	4.63	FM	2.5	0.000	N	-	[001]
Tc_2N_2Se	5.12	AFM stripe	3.5	0.620	N	-	[110]

Tc_2N_2Te	5.37	AFM stripe	3.5	0.593	N	-	[100]
Tc_2P_2O	4.08	AFM zigzag-X	1.4	0.000	N	-	[001]
Tc_2P_2S	4.67	AFM stripe	2.3	0.000	N	-	[001]
Tc_2P_2Se	4.67	FM	2.0	0.000	N	-	[001]
Tc_2P_2Te	4.94	FM	2.1	0.000	N	-	[001]
Tc_2As_2O	4.06	AFM zigzag-X	1.3	0.000	N	-	[001]
Tc_2As_2S	4.68	AFM stripe	2.3	0.000	N	-	[001]
Tc_2As_2Se	4.65	AM	1.9	0.000	N	-	[001]
Tc_2As_2Te	4.96	FM	2.1	0.000	N	-	[001]
Ru_2F_2O	4.18	AFM zigzag-Y	2.9	0.000	N	-	[110]
Ru_2F_2S	4.55	AM	2.8	0.000	N	-	[001]
Ru_2F_2Se	4.74	AM	2.8	0.000	N	-	[001]
Ru_2F_2Te	5.05	AM	2.8	0.000	N	-	[001]
Ru_2Cl_2O	4.17	FM	2.7	0.000	N	-	[110]
Ru_2Cl_2S	4.52	AFM stripe	2.1	0.000	N	-	[110]
Ru_2Cl_2Se	4.81	AM	2.6	0.000	N	-	[001]
Ru_2Cl_2Te	5.08	AM	2.6	0.000	N	-	[001]
Ru_2Br_2O	4.18	FM	2.6	0.000	N	-	[110]
Ru_2Br_2S	4.57	AFM stripe	2.2	0.000	N	-	[110]
Ru_2Br_2Se	4.88	AFM zigzag-Y	2.5	0.000	N	-	[001]
Ru_2Br_2Te	5.09	AM	2.5	0.000	N	-	[001]
Ru_2I_2O	4.21	FM	2.5	0.000	N	-	[100]
Ru_2I_2S	4.54	FM	1.6	0.000	N	-	[100]
Ru_2I_2Se	4.85	AFM stripe	2.2	0.000	Y	M-X	[100]
Ru_2I_2Te	5.11	AFM zigzag-X	2.0	0.000	N	-	[001]
Ru_2O_2O	3.85	FM	0.9	0.000	N	-	[001]
Ru_2O_2S	4.31	AFM stripe	2.0	0.000	N	-	[001]
Ru_2O_2Se	4.85	AFM zigzag-Y	2.9	0.334	N	-	[100]
Ru_2O_2Te	5.07	AFM zigzag-Y	2.8	0.000	N	-	[001]
Ru_2S_2O	3.99	FM	1.1	0.000	N	-	[001]
Ru_2S_2S	4.35	FM	0.8	0.000	N	-	[001]
Ru_2S_2Se	4.63	NM	0.0	0.000	N	-	-
Ru_2S_2Te	4.96	NM	0.0	0.000	N	-	-
Ru_2Se_2O	4.04	FM	1.1	0.000	N	-	[110]
Ru_2Se_2S	4.39	FM	0.8	0.000	N	-	[100]
Ru_2Se_2Se	4.65	FM	0.8	0.000	N	-	[001]
Ru_2Se_2Te	4.91	NM	0.0	0.000	N	-	-
Ru_2Te_2O	4.07	NM	0.0	0.000	N	-	-
Ru_2Te_2S	4.50	FM	0.9	0.000	N	-	[110]
Ru_2Te_2Se	4.69	FM	0.9	0.000	N	-	[100]
Ru_2Te_2Te	4.96	NM	0.0	0.000	N	-	-
Ru_2N_2O	4.09	AFM zigzag-X	1.2	0.000	N	-	[110]
Ru_2N_2S	4.45	FM	0.8	0.000	N	-	[001]
Ru_2N_2Se	4.65	AM	1.6	0.000	N	-	[100]
Ru_2N_2Te	4.94	AM	1.9	0.000	N	-	[001]

Ru_2P_2O	4.02	AM	0.4	0.000	N	-	[001]
Ru_2P_2S	4.54	FM	1.1	0.000	N	-	[001]
Ru_2P_2Se	4.72	FM	1.1	0.000	N	-	[001]
Ru_2P_2Te	5.04	AM	1.3	0.000	N	-	[001]
Ru_2As_2O	4.01	AM	0.5	0.000	N	-	[001]
Ru_2As_2S	4.43	FM	1.0	0.000	N	-	[100]
Ru_2As_2Se	4.73	FM	1.2	0.000	N	-	[100]
Ru_2As_2Te	5.06	AFM zigzag-Y	1.1	0.000	N	-	[110]
Rh_2F_2O	4.05	AFM zigzag-Y	1.3	0.000	N	-	[100]
Rh_2F_2S	4.41	AFM stripe	1.1	0.000	N	-	[100]
Rh_2F_2Se	4.62	AFM zigzag-X	1.2	0.000	N	-	[100]
Rh_2F_2Te	4.88	AFM zigzag-X	1.3	0.000	N	-	[110]
Rh_2Cl_2O	4.10	AM	1.2	0.000	N	-	[110]
Rh_2Cl_2S	4.49	AFM zigzag-Y	0.9	0.000	N	-	[100]
Rh_2Cl_2Se	4.69	AFM stripe	1.0	0.000	N	-	[110]
Rh_2Cl_2Te	4.97	AFM stripe	1.0	0.000	N	-	[110]
Rh_2Br_2O	4.12	AM	1.1	0.000	N	-	[001]
Rh_2Br_2S	4.52	AFM zigzag-Y	0.8	0.000	N	-	[100]
Rh_2Br_2Se	4.69	AFM zigzag-Y	0.9	0.000	N	-	[110]
Rh_2Br_2Te	4.99	AFM stripe	0.9	0.000	N	-	[110]
Rh_2I_2O	4.20	AM	0.6	0.000	N	-	[110]
Rh_2I_2S	4.56	AM	0.7	0.000	N	-	[001]
Rh_2I_2Se	4.74	AM	0.7	0.000	N	-	[001]
Rh_2I_2Te	4.98	AM	0.7	0.000	N	-	[001]
Rh_2O_2O	3.89	FM	0.3	0.000	N	-	[100]
Rh_2O_2S	4.26	NM	0.0	0.000	N	-	-
Rh_2O_2Se	4.47	AFM zigzag-X	0.2	0.000	N	-	[100]
Rh_2O_2Te	4.92	AFM stripe	0.8	0.000	N	-	[100]
Rh_2S_2O	4.05	FM	0.3	0.000	N	-	[100]
Rh_2S_2S	4.40	NM	0.0	0.000	N	-	-
Rh_2S_2Se	4.59	NM	0.0	0.000	N	-	-
Rh_2S_2Te	5.00	NM	0.0	0.000	N	-	-
Rh_2Se_2O	4.10	FM	0.4	0.000	N	-	[001]
Rh_2Se_2S	4.46	NM	0.0	0.000	N	-	-
Rh_2Se_2Se	4.63	NM	0.0	0.000	N	-	-
Rh_2Se_2Te	4.89	NM	0.0	0.000	N	-	-
Rh_2Te_2O	4.18	FM	0.4	0.000	N	-	[001]
Rh_2Te_2S	4.54	NM	0.0	0.000	N	-	-
Rh_2Te_2Se	4.71	NM	0.0	0.000	N	-	-
Rh_2Te_2Te	4.94	NM	0.0	0.000	N	-	-
Rh_2N_2O	4.16	NM	0.0	0.000	N	-	-
Rh_2N_2S	4.55	NM	0.0	0.000	N	-	-
Rh_2N_2Se	4.74	NM	0.0	0.000	N	-	-
Rh_2N_2Te	5.02	NM	0.0	0.000	N	-	-
Rh_2P_2O	4.22	FM	0.2	0.000	N	-	[001]

Rh_2P_2S	4.56	NM	0.0	0.000	N	-	-
Rh_2P_2Se	4.71	NM	0.0	0.000	N	-	-
Rh_2P_2Te	4.92	NM	0.0	0.000	N	-	-
Rh_2As_2O	4.19	FM	0.2	0.000	N	-	[001]
Rh_2As_2S	4.59	NM	0.0	0.000	N	-	-
Rh_2As_2Se	4.74	NM	0.0	0.000	N	-	-
Rh_2As_2Te	4.95	NM	0.0	0.000	N	-	-
Pd_2F_2O	4.05	AFM stripe	1.4	0.509	N	-	[001]
Pd_2F_2S	4.37	NM	0.0	0.000	N	-	-
Pd_2F_2Se	4.58	NM	0.0	0.000	N	-	-
Pd_2F_2Te	4.84	NM	0.0	0.000	N	-	-
Pd_2Cl_2O	4.12	AFM zigzag-Y	0.7	0.000	N	-	[001]
Pd_2Cl_2S	4.46	NM	0.0	0.000	N	-	-
Pd_2Cl_2Se	4.66	NM	0.0	0.000	N	-	-
Pd_2Cl_2Te	4.94	NM	0.0	0.000	N	-	-
Pd_2Br_2O	4.12	AFM zigzag-Y	0.7	0.000	N	-	[001]
Pd_2Br_2S	4.50	NM	0.0	0.000	N	-	-
Pd_2Br_2Se	4.70	NM	0.0	0.000	N	-	-
Pd_2Br_2Te	4.97	NM	0.0	0.000	N	-	-
Pd_2I_2O	4.16	FM	0.2	0.000	N	-	[100]
Pd_2I_2S	4.56	FM	0.1	0.000	N	-	[001]
Pd_2I_2Se	4.77	FM	0.1	0.000	N	-	[001]
Pd_2I_2Te	5.01	NM	0.0	0.000	N	-	-
Pd_2O_2O	3.91	AFM zigzag-X	0.2	0.000	N	-	[100]
Pd_2O_2S	4.58	AFM stripe	0.6	0.000	N	-	[001]
Pd_2O_2Se	4.75	AFM zigzag-X	0.6	0.000	N	-	[001]
Pd_2O_2Te	5.00	FM	0.2	0.000	N	-	[100]
Pd_2S_2O	4.09	NM	0.0	0.000	N	-	-
Pd_2S_2S	4.72	NM	0.0	0.000	N	-	-
Pd_2S_2Se	4.87	NM	0.0	0.000	N	-	-
Pd_2S_2Te	5.10	NM	0.0	0.000	N	-	-
Pd_2Se_2O	4.15	NM	0.0	0.000	N	-	-
Pd_2Se_2S	4.77	NM	0.0	0.000	N	-	-
Pd_2Se_2Se	4.92	NM	0.0	0.000	N	-	-
Pd_2Se_2Te	5.14	NM	0.0	0.000	N	-	-
Pd_2Te_2O	4.24	NM	0.0	0.000	N	-	-
Pd_2Te_2S	4.68	NM	0.0	0.000	N	-	-
Pd_2Te_2Se	4.99	NM	0.0	0.000	N	-	-
Pd_2Te_2Te	5.20	NM	0.0	0.000	N	-	-
Pd_2N_2O	4.34	AFM stripe	0.7	0.000	N	-	[001]
Pd_2N_2S	4.75	AFM stripe	0.5	0.000	N	-	[001]
Pd_2N_2Se	4.93	AFM stripe	0.5	0.000	N	-	[001]
Pd_2N_2Te	5.17	NM	0.0	0.000	N	-	-
Pd_2P_2O	4.32	NM	0.0	0.000	N	-	-
Pd_2P_2S	4.72	NM	0.0	0.000	N	-	-

Pd_2P_2Se	4.87	NM	0.0	0.000	N	-	-
Pd_2P_2Te	5.07	NM	0.0	0.000	N	-	-
Pd_2As_2O	4.34	NM	0.0	0.000	N	-	-
Pd_2As_2S	4.74	NM	0.0	0.000	N	-	-
Pd_2As_2Se	4.89	NM	0.0	0.000	N	-	-
Pd_2As_2Te	5.10	NM	0.0	0.000	N	-	-
Ag_2F_2O	4.16	NM	0.0	0.000	N	-	-
Ag_2F_2S	4.59	NM	0.0	0.000	N	-	-
Ag_2F_2Se	4.80	NM	0.0	0.000	N	-	-
Ag_2F_2Te	5.11	NM	0.0	0.000	N	-	-
Ag_2Cl_2O	4.27	NM	0.0	0.000	N	-	-
Ag_2Cl_2S	4.75	NM	0.0	0.000	N	-	-
Ag_2Cl_2Se	4.95	NM	0.0	0.000	N	-	-
Ag_2Cl_2Te	5.23	NM	0.0	0.000	N	-	-
Ag_2Br_2O	4.30	NM	0.0	0.000	N	-	-
Ag_2Br_2S	4.80	NM	0.0	0.000	N	-	-
Ag_2Br_2Se	5.01	NM	0.0	0.000	N	-	-
Ag_2Br_2Te	5.29	NM	0.0	0.000	N	-	-
Ag_2I_2O	4.36	NM	0.0	0.000	N	-	-
Ag_2I_2S	4.86	NM	0.0	0.000	N	-	-
Ag_2I_2Se	5.08	NM	0.0	0.000	N	-	-
Ag_2I_2Te	5.37	NM	0.0	0.000	N	-	-
Ag_2O_2O	4.48	FM	0.1	0.000	N	-	[001]
Ag_2O_2S	4.89	FM	0.1	0.000	N	-	[100]
Ag_2O_2Se	5.08	NM	0.0	0.000	N	-	-
Ag_2O_2Te	5.29	NM	0.0	0.000	N	-	-
Ag_2S_2O	4.67	NM	0.0	0.000	N	-	-
Ag_2S_2S	5.04	NM	0.0	0.000	N	-	-
Ag_2S_2Se	5.20	NM	0.0	0.000	N	-	-
Ag_2S_2Te	5.44	NM	0.0	0.000	N	-	-
Ag_2Se_2O	4.71	NM	0.0	0.000	N	-	-
Ag_2Se_2S	5.10	NM	0.0	0.000	N	-	-
Ag_2Se_2Se	5.24	NM	0.0	0.000	N	-	-
Ag_2Se_2Te	5.47	NM	0.0	0.000	N	-	-
Ag_2Te_2O	4.45	NM	0.0	0.000	N	-	-
Ag_2Te_2S	5.19	NM	0.0	0.000	N	-	-
Ag_2Te_2Se	5.34	NM	0.0	0.000	N	-	-
Ag_2Te_2Te	5.55	NM	0.0	0.000	N	-	-
Ag_2N_2O	4.83	NM	0.0	0.426	N	-	-
Ag_2N_2S	5.14	NM	0.0	0.962	N	-	-
Ag_2N_2Se	5.27	NM	0.0	0.999	N	-	-
Ag_2N_2Te	5.46	NM	0.0	1.080	N	-	-
Ag_2P_2O	4.66	NM	0.0	0.000	N	-	-
Ag_2P_2S	5.06	NM	0.0	0.000	N	-	-
Ag_2P_2Se	5.19	NM	0.0	0.000	N	-	-

Ag_2P_2Te	5.45	NM	0.0	0.000	N	-	-
Ag_2As_2O	4.69	NM	0.0	0.000	N	-	-
Ag_2As_2S	5.11	NM	0.0	0.000	N	-	-
Ag_2As_2Se	5.24	NM	0.0	0.000	N	-	-
Ag_2As_2Te	5.46	NM	0.0	0.000	N	-	-
Cd_2F_2O	4.32	NM	0.0	1.720	N	-	-
Cd_2F_2S	4.77	NM	0.0	1.963	N	-	-
Cd_2F_2Se	4.94	NM	0.0	1.390	N	-	-
Cd_2F_2Te	5.22	NM	0.0	0.332	N	-	-
Cd_2Cl_2O	4.45	NM	0.0	2.135	N	-	-
Cd_2Cl_2S	4.94	NM	0.0	1.389	N	-	-
Cd_2Cl_2Se	5.11	NM	0.0	0.809	N	-	-
Cd_2Cl_2Te	5.39	NM	0.0	0.000	N	-	-
Cd_2Br_2O	4.49	NM	0.0	2.187	N	-	-
Cd_2Br_2S	5.00	NM	0.0	1.018	N	-	-
Cd_2Br_2Se	5.17	NM	0.0	0.455	N	-	-
Cd_2Br_2Te	5.46	NM	0.0	0.000	N	-	-
Cd_2I_2O	4.54	NM	0.0	2.383	N	-	-
Cd_2I_2S	5.07	NM	0.0	0.522	N	-	-
Cd_2I_2Se	5.25	NM	0.0	0.000	N	-	-
Cd_2I_2Te	5.55	NM	0.0	0.000	N	-	-
Cd_2O_2O	4.51	NM	0.0	0.722	N	-	-
Cd_2O_2S	4.87	NM	0.0	0.561	N	-	-
Cd_2O_2Se	4.99	NM	0.0	0.173	N	-	-
Cd_2O_2Te	5.24	NM	0.0	0.000	N	-	-
Cd_2S_2O	4.78	NM	0.0	0.615	N	-	-
Cd_2S_2S	5.17	NM	0.0	0.000	N	-	-
Cd_2S_2Se	5.30	NM	0.0	0.000	N	-	-
Cd_2S_2Te	5.54	NM	0.0	0.000	N	-	-
Cd_2Se_2O	4.82	NM	0.0	0.316	N	-	-
Cd_2Se_2S	5.24	NM	0.0	0.000	N	-	-
Cd_2Se_2Se	5.38	NM	0.0	0.000	N	-	-
Cd_2Se_2Te	5.62	NM	0.0	0.000	N	-	-
Cd_2Te_2O	4.56	NM	0.0	0.000	N	-	-
Cd_2Te_2S	5.37	NM	0.0	0.000	N	-	-
Cd_2Te_2Se	5.52	NM	0.0	0.000	N	-	-
Cd_2Te_2Te	5.75	NM	0.0	0.000	N	-	-
Cd_2N_2O	4.59	NM	0.0	0.000	N	-	-
Cd_2N_2S	5.50	NM	0.0	0.000	N	-	-
Cd_2N_2Se	5.65	NM	0.0	0.000	N	-	-
Cd_2N_2Te	5.85	NM	0.0	0.000	N	-	-
Cd_2P_2O	4.77	NM	0.0	0.000	N	-	-
Cd_2P_2S	5.20	NM	0.0	0.000	N	-	-
Cd_2P_2Se	5.37	NM	0.0	0.000	N	-	-
Cd_2P_2Te	5.62	NM	0.0	0.000	N	-	-

Cd_2As_2O	4.79	NM	0.0	0.000	N	-	-
Cd_2As_2S	5.26	NM	0.0	0.000	N	-	-
Cd_2As_2Se	5.41	NM	0.0	0.000	N	-	-
Cd_2As_2Te	5.67	NM	0.0	0.000	N	-	-

TABLE S2: Summary of key properties for all 220 candidates from the structural framework M_2A_2.

Material	Lattice constant (Å)	Magnetic order	Moment (μ_B)	Band gap (eV)	Dirac-cone	K-location	MA
Sc_2F_2	4.05	FM	0.6	0.000	N	-	[001]
Sc_2Cl_2	4.32	FM	0.6	0.000	N	-	[001]
Sc_2Br_2	4.40	FM	0.7	0.000	N	-	[100]
Sc_2I_2	4.47	FM	0.7	0.000	N	-	[100]
Sc_2O_2	3.86	NM	0.0	0.000	N	-	-
Sc_2S_2	4.26	NM	0.0	0.000	N	-	-
Sc_2Se_2	4.37	NM	0.0	0.000	N	-	-
Sc_2Te_2	4.53	FM	0.4	0.000	N	-	[001]
Sc_2N_2	4.18	NM	0.0	0.000	N	-	-
Sc_2P_2	4.73	NM	0.0	0.000	N	-	-
Sc_2As_2	4.81	FM	0.1	0.000	N	-	[001]
Ti_2F_2	4.03	AM	1.6	0.129	N	-	[001]
Ti_2Cl_2	4.22	AM	1.6	0.000	N	-	[100]
Ti_2Br_2	4.29	AM	1.5	0.578	N	-	[001]
Ti_2I_2	4.37	AM	1.5	0.000	N	-	[001]
Ti_2O_2	3.62	AM	0.5	0.000	N	-	[001]
Ti_2S_2	3.94	AFM zigzag-Y	0.9	0.000	N	-	[100]
Ti_2Se_2	4.04	AFM zigzag-Y	1.0	0.000	N	-	[100]
Ti_2Te_2	4.28	AFM stripe	1.3	0.000	Y	M-X, M-Y	[100]
Ti_2N_2	3.96	AM	0.5	0.000	N	-	[001]
Ti_2P_2	4.48	AFM zigzag-Y	1.1	0.000	N	-	[100]
Ti_2As_2	4.45	FM	1.2	0.000	N	-	[100]
V_2F_2	3.81	AM	2.6	0.000	Y	M-X, M-Y	[001]
V_2Cl_2	3.96	AM	2.6	0.174	N	-	[001]
V_2Br_2	4.03	AM	2.6	0.251	N	-	[001]
V_2I_2	4.15	AM	2.7	0.351	N	-	[001]
V_2O_2	3.51	AM	1.7	0.000	Y	M-X, M-Y	[001]
V_2S_2	3.80	AM	2.0	0.000	N	-	[100]
V_2Se_2	3.95	AM	2.3	0.000	N	-	[100]
V_2Te_2	4.14	AM	2.4	0.000	N	-	[100]
V_2N_2	3.44	NM	0.0	0.000	N	-	-
V_2P_2	3.76	AM	1.5	0.000	N	-	[100]
V_2As_2	3.69	AM	1.6	0.000	N	-	[100]
Cr_2F_2	3.85	AM	4.0	0.000	N	-	[001]
Cr_2Cl_2	4.03	AM	4.1	0.133	N	-	[001]
Cr_2Br_2	4.11	AM	4.1	0.233	N	-	[001]
Cr_2I_2	4.28	AM	4.1	0.084	N	-	[110]
Cr_2O_2	3.44	AM	3.1	0.000	Y	M-X, M-Y	[100]

Cr_2S_2	3.80	AM	3.4	0.749	N	-	[100]
Cr_2Se_2	3.94	AM	3.6	0.573	N	-	[100]
Cr_2Te_2	4.16	AM	3.7	0.142	N	-	[001]
Cr_2N_2	3.83	AFM stripe	3.4	0.000	Y	Γ-X	[110]
Cr_2P_2	4.28	AFM stripe	3.7	0.350	N	-	[110]
Cr_2As_2	4.37	AFM zigzag-X	3.8	0.000	N	-	[100]
Mn_2F_2	3.95	AM	4.6	0.560	N	-	[001]
Mn_2Cl_2	4.12	AM	4.6	0.548	N	-	[001]
Mn_2Br_2	4.16	AM	4.6	0.506	N	-	[100]
Mn_2I_2	4.23	AM	4.6	0.314	N	-	[110]
Mn_2O_2	3.58	AM	4.2	0.000	N	-	[001]
Mn_2S_2	3.92	AM	4.3	0.000	N	-	[100]
Mn_2Se_2	4.05	AM	4.4	0.000	N	-	[100]
Mn_2Te_2	4.21	AM	4.4	0.000	N	-	[100]
Mn_2N_2	3.89	AM	4.1	0.000	N	-	[001]
Mn_2P_2	4.34	FM	4.3	0.000	N	-	[001]
Mn_2As_2	4.44	FM	4.4	0.000	N	-	[001]
Fe_2F_2	3.72	AFM zigzag-Y	3.7	0.748	N	-	[001]
Fe_2Cl_2	3.77	AFM zigzag-X	3.2	0.000	N	-	[001]
Fe_2Br_2	3.85	AFM zigzag-X	3.2	0.000	N	-	[001]
Fe_2I_2	4.14	AM	3.0	0.000	Y	M-X, M-Y	[001]
Fe_2O_2	3.49	AM	3.6	0.681	N	-	[001]
Fe_2S_2	3.80	AM	3.5	0.000	N	-	[001]
Fe_2Se_2	3.92	AM	3.5	0.000	N	-	[001]
Fe_2Te_2	4.11	AM	3.4	0.000	N	-	[001]
Fe_2N_2	3.76	AFM zigzag-Y	3.4	0.000	N	-	[001]
Fe_2P_2	4.06	AFM stripe	3.2	0.000	N	-	[001]
Fe_2As_2	3.59	FM	3.0	0.000	N	-	[001]
Co_2F_2	3.62	AFM zigzag-X	2.4	0.175	N	-	[001]
Co_2Cl_2	3.74	AFM zigzag-Y	2.0	0.670	N	-	[100]
Co_2Br_2	3.84	AFM zigzag-X	2.0	0.619	N	-	[100]
Co_2I_2	4.04	AFM zigzag-X	2.0	0.636	N	-	[001]
Co_2O_2	3.41	AFM zigzag-Y	2.6	0.000	N	-	[001]
Co_2S_2	3.71	FM	2.1	0.000	N	-	[100]
Co_2Se_2	3.81	FM	2.2	0.000	N	-	[001]
Co_2Te_2	3.99	FM	2.1	0.000	N	-	[001]
Co_2N_2	3.69	AFM zigzag-Y	1.8	0.000	N	-	[110]
Co_2P_2	3.98	AFM zigzag-Y	2.1	0.000	N	-	[001]
Co_2As_2	3.59	AFM zigzag-X	2.0	0.000	N	-	[001]
Ni_2F_2	3.47	AM	1.1	1.016	N	-	[100]
Ni_2Cl_2	3.64	AM	1.0	1.643	N	-	[100]
Ni_2Br_2	3.76	AM	1.0	1.668	N	-	[001]
Ni_2I_2	3.97	AM	0.9	1.271	N	-	[001]
Ni_2O_2	3.23	FM	1.1	0.000	N	-	[001]
Ni_2S_2	3.51	FM	0.5	0.000	N	-	[001]

Ni_2Se_2	3.60	FM	0.6	0.000	N	-	[100]
Ni_2Te_2	3.70	AFM zigzag-Y	0.4	0.000	N	-	[110]
Ni_2N_2	3.58	AFM zigzag-Y	0.5	0.000	N	-	[001]
Ni_2P_2	3.46	NM	0.0	0.000	N	-	-
Ni_2As_2	3.32	NM	0.0	0.000	N	-	-
Cu_2F_2	3.52	NM	0.0	1.322	N	-	-
Cu_2Cl_2	3.70	NM	0.0	1.525	N	-	-
Cu_2Br_2	3.83	NM	0.0	1.288	N	-	-
Cu_2I_2	4.05	NM	0.0	0.872	N	-	-
Cu_2O_2	3.35	AFM zigzag-Y	0.4	0.311	N	-	[110]
Cu_2S_2	3.61	NM	0.0	0.000	N	-	-
Cu_2Se_2	3.72	NM	0.0	0.000	N	-	-
Cu_2Te_2	3.92	NM	0.0	0.000	N	-	-
Cu_2N_2	3.68	NM	0.0	0.000	N	-	-
Cu_2P_2	3.94	NM	0.0	0.000	N	-	-
Cu_2As_2	4.03	NM	0.0	0.000	N	-	-
Zn_2F_2	3.64	NM	0.0	0.949	N	-	-
Zn_2Cl_2	3.73	NM	0.0	1.245	N	-	-
Zn_2Br_2	3.79	NM	0.0	1.026	N	-	-
Zn_2I_2	3.93	NM	0.0	0.218	N	-	-
Zn_2O_2	3.46	NM	0.0	0.000	N	-	-
Zn_2S_2	3.77	NM	0.0	0.000	N	-	-
Zn_2Se_2	3.87	NM	0.0	0.000	N	-	-
Zn_2Te_2	4.05	NM	0.0	0.000	N	-	-
Zn_2N_2	3.70	NM	0.0	0.000	N	-	-
Zn_2P_2	3.81	NM	0.0	0.000	N	-	-
Zn_2As_2	3.89	NM	0.0	0.000	N	-	-
Y_2F_2	4.49	FM	0.7	0.000	N	-	[001]
Y_2Cl_2	4.86	FM	0.6	0.000	N	-	[001]
Y_2Br_2	4.92	FM	0.6	0.000	N	-	[100]
Y_2I_2	5.02	FM	0.5	0.000	N	-	[100]
Y_2O_2	4.32	NM	0.0	0.000	N	-	-
Y_2S_2	4.75	NM	0.0	0.000	N	-	-
Y_2Se_2	4.85	NM	0.0	0.000	N	-	-
Y_2Te_2	5.02	FM	0.1	0.000	N	-	[001]
Y_2N_2	4.61	NM	0.0	0.000	N	-	-
Y_2P_2	5.19	NM	0.0	0.000	N	-	-
Y_2As_2	5.28	NM	0.0	0.000	N	-	-
Zr_2F_2	4.14	AM	0.3	0.000	N	-	[001]
Zr_2Cl_2	4.21	NM	0.0	0.000	N	-	-
Zr_2Br_2	4.24	NM	0.0	0.000	N	-	-
Zr_2I_2	4.26	NM	0.0	0.000	N	-	-
Zr_2O_2	3.96	NM	0.0	0.000	N	-	-
Zr_2S_2	4.26	NM	0.0	0.000	N	-	-
Zr_2Se_2	4.33	NM	0.0	0.000	N	-	-

Zr_2Te_2	4.43	NM	0.0	0.000	N	-	-
Zr_2N_2	4.27	NM	0.0	0.000	N	-	-
Zr_2P_2	4.75	NM	0.0	0.000	N	-	-
Zr_2As_2	4.47	FM	0.4	0.000	N	-	[001]
Nb_2F_2	4.03	AM	2.0	0.000	Y	M-X, M-Y	[001]
Nb_2Cl_2	4.08	AM	1.8	0.000	Y	M-X, M-Y	[001]
Nb_2Br_2	4.09	AM	1.7	0.000	Y	M-X, M-Y	[001]
Nb_2I_2	4.12	AM	1.6	0.000	N	-	[001]
Nb_2O_2	3.72	NM	0.0	0.000	N	-	-
Nb_2S_2	3.93	AFM zigzag-X	0.1	0.000	N	-	[100]
Nb_2Se_2	3.97	AM	0.3	0.000	N	-	[100]
Nb_2Te_2	4.04	AM	0.3	0.000	N	-	[100]
Nb_2N_2	3.78	NM	0.0	0.000	N	-	-
Nb_2P_2	4.02	NM	0.0	0.000	N	-	-
Nb_2As_2	3.98	AM	0.1	0.000	N	-	[001]
Mo_2F_2	4.01	AM	3.0	0.000	N	-	[001]
Mo_2Cl_2	3.72	AFM zigzag-Y	0.3	0.000	N	-	[001]
Mo_2Br_2	3.88	AM	1.1	0.000	N	-	[001]
Mo_2I_2	3.98	AM	1.3	0.000	N	-	[001]
Mo_2O_2	3.66	AM	1.7	0.000	Y	M-X, M-Y	[001]
Mo_2S_2	3.85	AM	1.7	0.000	Y	M-X, M-Y	[001]
Mo_2Se_2	3.90	AM	1.7	0.000	Y	M-X, M-Y	[001]
Mo_2Te_2	3.97	AM	1.8	0.000	Y	M-X, M-Y	[001]
Mo_2N_2	3.56	AFM zigzag-Y	0.2	0.000	N	-	[001]
Mo_2P_2	3.74	AM	0.5	0.000	N	-	[001]
Mo_2As_2	3.69	NM	0.0	0.000	N	-	-
Tc_2F_2	3.86	AM	3.2	0.000	N	-	[001]
Tc_2Cl_2	3.81	AM	2.7	0.000	N	-	[001]
Tc_2Br_2	3.85	AM	2.7	0.000	N	-	[001]
Tc_2I_2	3.98	AM	2.7	0.000	N	-	[110]
Tc_2O_2	3.53	AFM zigzag-Y	1.3	0.000	N	-	[100]
Tc_2S_2	3.75	AM	1.5	0.000	Y	M-X, M-Y	[001]
Tc_2Se_2	3.81	AM	1.5	0.000	N	-	[001]
Tc_2Te_2	3.91	AM	1.6	0.000	N	-	[001]
Tc_2N_2	3.53	AM	1.1	0.000	Y	M-X, M-Y	[001]
Tc_2P_2	3.77	AM	1.5	0.000	Y	M-X, M-Y	[001]
Tc_2As_2	3.69	AM	1.4	0.000	Y	M-X, M-Y	[100]
Ru_2F_2	3.87	FM	2.9	0.000	N	-	[001]
Ru_2Cl_2	3.85	FM	2.7	0.000	N	-	[001]
Ru_2Br_2	3.97	FM	2.6	0.000	N	-	[001]
Ru_2I_2	4.14	FM	2.3	0.000	N	-	[001]
Ru_2O_2	3.51	AFM zigzag-X	0.5	0.000	N	-	[001]
Ru_2S_2	3.74	FM	1.1	0.000	N	-	[001]
Ru_2Se_2	3.86	FM	1.2	0.000	N	-	[001]
Ru_2Te_2	3.96	FM	1.3	0.000	N	-	[001]

Ru_2N_2	3.50	AFM zigzag-X	1.1	0.000	N	-	[100]
Ru_2P_2	3.86	AFM zigzag-X	0.9	0.000	N	-	[100]
Ru_2As_2	3.80	FM	1.6	0.000	N	-	[001]
Rh_2F_2	3.70	FM	1.8	0.000	N	-	[001]
Rh_2Cl_2	3.79	AFM zigzag-X	1.1	0.000	N	-	[001]
Rh_2Br_2	3.93	AM	0.6	0.000	N	-	[100]
Rh_2I_2	4.02	NM	0.0	0.000	N	-	-
Rh_2O_2	3.56	NM	0.0	0.000	N	-	-
Rh_2S_2	3.77	NM	0.0	0.000	N	-	-
Rh_2Se_2	3.82	NM	0.0	0.000	N	-	-
Rh_2Te_2	3.91	FM	0.1	0.000	N	-	[001]
Rh_2N_2	3.90	NM	0.0	0.000	N	-	-
Rh_2P_2	3.96	FM	0.2	0.000	N	-	[001]
Rh_2As_2	3.94	NM	0.0	0.000	N	-	-
Pd_2F_2	3.88	AFM stripe	0.9	1.458	N	-	[100]
Pd_2Cl_2	3.99	AM	0.8	2.024	N	-	[100]
Pd_2Br_2	4.07	AM	0.8	1.624	N	-	[001]
Pd_2I_2	4.13	AM	0.6	0.545	N	-	[001]
Pd_2O_2	3.62	FM	0.3	0.000	N	-	[100]
Pd_2S_2	3.87	NM	0.0	0.000	N	-	-
Pd_2Se_2	3.98	FM	0.2	0.000	N	-	[100]
Pd_2Te_2	4.05	FM	0.2	0.000	N	-	[001]
Pd_2N_2	4.21	NM	0.0	0.195	N	-	-
Pd_2P_2	4.30	FM	0.2	0.000	N	-	[001]
Pd_2As_2	3.77	NM	0.0	0.000	N	-	-
Ag_2F_2	4.02	NM	0.0	1.363	N	-	-
Ag_2Cl_2	4.16	NM	0.0	2.037	N	-	-
Ag_2Br_2	4.23	NM	0.0	1.851	N	-	-
Ag_2I_2	4.38	NM	0.0	1.446	N	-	-
Ag_2O_2	3.81	NM	0.0	0.000	N	-	-
Ag_2S_2	4.04	NM	0.0	0.000	N	-	-
Ag_2Se_2	4.13	NM	0.0	0.000	N	-	-
Ag_2Te_2	4.27	NM	0.0	0.000	N	-	-
Ag_2N_2	4.30	NM	0.0	0.000	N	-	-
Ag_2P_2	4.41	NM	0.0	0.000	N	-	-
Ag_2As_2	4.48	NM	0.0	0.000	N	-	-
Cd_2F_2	4.10	NM	0.0	0.462	N	-	-
Cd_2Cl_2	4.20	NM	0.0	0.835	N	-	-
Cd_2Br_2	4.23	NM	0.0	0.758	N	-	-
Cd_2I_2	4.28	NM	0.0	0.616	N	-	-
Cd_2O_2	3.93	NM	0.0	0.000	N	-	-
Cd_2S_2	4.19	NM	0.0	0.000	N	-	-
Cd_2Se_2	4.29	NM	0.0	0.000	N	-	-
Cd_2Te_2	4.43	NM	0.0	0.000	N	-	-
Cd_2N_2	4.60	NM	0.0	0.000	N	-	-

Cd_2P_2	4.27	NM	0.0	0.000	N	-	-
Cd_2As_2	4.29	NM	0.0	0.000	N	-	-

TABLE S3: Summary of key properties for all 1200 candidates from the structural framework Janus $M_2AA'B$.

Material	Lattice constant (Å)	Magnetic order	Moment (μ_B)	Band gap (eV)	Dirac-cone	K-location	MA
Sc_2FClO	4.18	NM	0.0	0.000	N	-	-
Sc_2FClS	4.43	NM	0.0	0.000	N	-	-
Sc_2FClSe	4.45	AFM Zigzag-Y	0.4	0.000	N	-	[110]
Sc_2FClTe	4.46	FM	0.7	0.000	N	-	[100]
Sc_2FBrO	4.19	NM	0.0	0.000	N	-	-
Sc_2FBrS	4.46	AFM Zigzag-Y	0.3	0.000	N	-	[110]
Sc_2FBrSe	4.48	AFM Zigzag-Y	0.4	0.000	N	-	[110]
Sc_2FBrTe	4.49	FM	0.7	0.000	N	-	[100]
Sc_2FIO	4.21	NM	0.0	0.000	N	-	-
Sc_2FIS	4.50	AFM Zigzag-Y	0.3	0.000	N	-	[100]
Sc_2FISe	4.52	AFM Zigzag-Y	0.4	0.000	N	-	[110]
Sc_2FITe	4.54	FM	0.6	0.000	N	-	[001]
Sc_2ClBrO	4.31	FM	0.6	0.000	N	-	[100]
Sc_2ClBrS	4.73	NM	0.0	0.000	N	-	-
$Sc_2ClBrSe$	4.80	NM	0.0	0.000	N	-	-
$Sc_2ClBrTe$	4.92	AFM Zigzag-Y	0.3	0.000	N	-	[100]
Sc_2ClIO	4.30	FM	0.5	0.000	N	-	[100]
Sc_2ClIS	4.76	NM	0.0	0.000	N	-	-
Sc_2ClISe	4.84	NM	0.0	0.000	N	-	-
Sc_2ClITe	4.96	AFM Zigzag-Y	0.3	0.000	N	-	[100]
Sc_2BrIO	4.30	FM	0.5	0.000	N	-	[100]
Sc_2BrIS	4.85	NM	0.0	0.000	N	-	-
Sc_2BrISe	4.93	NM	0.0	0.000	N	-	-
Sc_2BrITe	5.07	AFM Zigzag-Y	0.2	0.000	N	-	[001]
Sc_2OSO	4.14	NM	0.0	0.295	N	-	-
Sc_2OSS	4.32	NM	0.0	0.000	N	-	-
Sc_2OSSe	4.51	NM	0.0	0.000	N	-	-
Sc_2OSTe	4.32	NM	0.0	0.000	N	-	-
Sc_2OSeO	4.16	NM	0.0	0.393	N	-	-
Sc_2OSeS	4.35	NM	0.0	0.000	N	-	-
Sc_2OSeSe	4.57	NM	0.0	0.000	N	-	-
Sc_2OSeTe	4.25	NM	0.0	0.000	N	-	-
Sc_2OTeO	4.19	NM	0.0	0.165	N	-	-
Sc_2OTeS	4.40	NM	0.0	0.000	N	-	-
Sc_2OTeSe	4.65	NM	0.0	0.000	N	-	-
Sc_2OTeTe	4.36	NM	0.0	0.000	N	-	-
Sc_2SSeO	4.31	NM	0.0	0.451	N	-	-
Sc_2SSeS	4.72	NM	0.0	0.000	N	-	-
Sc_2SSeSe	4.79	NM	0.0	0.000	N	-	-
Sc_2SSeTe	4.85	NM	0.0	0.107	N	-	-

Sc_2STeO	4.34	NM	0.0	0.534	N	-	-
Sc_2STeS	4.78	NM	0.0	0.000	N	-	-
Sc_2STeSe	4.90	NM	0.0	0.000	N	-	-
Sc_2STeTe	4.97	NM	0.0	0.000	N	-	-
Sc_2SeTeO	4.37	NM	0.0	0.527	N	-	-
Sc_2SeTeS	4.85	NM	0.0	0.000	N	-	-
$Sc_2SeTeSe$	4.91	NM	0.0	0.000	N	-	-
$Sc_2SeTeTe$	5.04	NM	0.0	0.000	N	-	-
Sc_2NPO	4.34	NM	0.0	0.850	N	-	-
Sc_2NPS	4.51	NM	0.0	0.335	N	-	-
Sc_2NPSe	4.51	NM	0.0	0.275	N	-	-
Sc_2NPTe	4.51	NM	0.0	0.089	N	-	-
Sc_2NAsO	4.34	NM	0.0	0.909	N	-	-
Sc_2NAsS	4.51	NM	0.0	0.298	N	-	-
Sc_2NAsSe	4.51	NM	0.0	0.247	N	-	-
Sc_2NAsTe	4.57	FM	0.2	0.000	N	-	[001]
Sc_2PAsO	4.60	NM	0.0	0.648	N	-	-
Sc_2PAsS	4.99	NM	0.0	0.000	N	-	-
Sc_2PAsSe	5.14	NM	0.0	0.000	N	-	-
Sc_2PAsTe	5.11	NM	0.0	0.000	N	-	-
Ti_2FClO	4.18	AM	1.6	1.536	N	-	[100]
Ti_2FClS	4.46	AFM Zigzag-Y	1.6	0.921	N	-	[100]
Ti_2FClSe	4.44	AFM Zigzag-Y	1.6	0.642	N	-	[100]
Ti_2FClTe	4.25	AFM Zigzag-Y	1.5	0.794	N	-	[100]
Ti_2FBrO	4.20	AM	1.6	1.478	N	-	[100]
Ti_2FBrS	4.49	AFM Zigzag-Y	1.6	0.963	N	-	[100]
Ti_2FBrSe	4.30	AFM Zigzag-Y	1.6	0.216	N	-	[100]
Ti_2FBrTe	4.54	AFM Zigzag-Y	1.6	0.622	N	-	[100]
Ti_2FIO	4.22	AM	1.6	1.629	N	-	[100]
Ti_2FIS	4.67	AFM Zigzag-X	1.6	1.411	N	-	[100]
Ti_2FISe	4.57	AFM Zigzag-Y	1.6	0.775	N	-	[100]
Ti_2FITe	5.13	AFM Stripe	1.7	1.837	N	-	[100]
Ti_2ClBrO	4.25	AM	1.5	1.568	N	-	[100]
Ti_2ClBrS	4.78	AM	1.6	1.843	N	-	[100]
$Ti_2ClBrSe$	4.85	AM	1.6	1.926	N	-	[100]
$Ti_2ClBrTe$	4.97	AM	1.6	1.605	N	-	[001]
Ti_2ClIO	4.27	AM	1.5	1.521	N	-	[100]
Ti_2ClIS	4.81	AM	1.6	1.776	N	-	[100]
Ti_2ClISe	4.97	AM	1.6	2.013	N	-	[001]
Ti_2ClITe	5.01	AM	1.6	1.548	N	-	[001]
Ti_2BrIO	4.28	AM	1.5	1.485	N	-	[100]
Ti_2BrIS	4.89	AM	1.6	1.587	N	-	[001]
Ti_2BrISe	4.98	AM	1.6	1.935	N	-	[001]
Ti_2BrITe	5.13	AM	1.6	1.763	N	-	[001]
Ti_2OSO	3.93	AM	0.7	1.164	N	-	[001]

Ti_2OSS	4.11	FM	0.8	0.000	N	-	[100]
Ti_2OSSe	4.23	FM	1.0	0.000	N	-	[100]
Ti_2OSTe	4.07	FM	0.9	0.000	N	-	[100]
Ti_2OSeO	3.96	AM	0.7	1.074	N	-	[001]
Ti_2OSeS	4.27	FM	0.9	0.000	N	-	[100]
Ti_2OSeSe	4.30	FM	1.0	0.000	N	-	[100]
Ti_2OSeTe	4.11	FM	1.0	0.000	N	-	[100]
Ti_2OTeO	4.00	AM	0.7	0.695	N	-	[001]
Ti_2OTeS	4.13	AM	0.7	0.000	N	-	[100]
Ti_2OTeSe	4.40	FM	1.0	0.000	N	-	[100]
Ti_2OTeTe	5.25	AFM Zigzag-Y	1.6	0.251	N	-	[001]
Ti_2SSeO	4.11	AFM Zigzag-X	0.8	0.895	N	-	[001]
Ti_2SSeS	4.66	AFM Zigzag-X	0.9	0.553	N	-	[100]
Ti_2SSeSe	4.53	AM	0.8	0.512	N	-	[001]
Ti_2SSeTe	4.57	FM	1.2	0.000	N	-	[100]
Ti_2STeO	4.17	AFM Zigzag-X	0.8	0.659	N	-	[001]
Ti_2STeS	4.52	AM	0.8	0.612	N	-	[100]
Ti_2STeSe	4.58	AM	0.8	0.472	N	-	[100]
Ti_2STeTe	4.67	AFM Zigzag-Y	1.3	0.000	N	-	[110]
Ti_2SeTeO	4.19	FM	1.0	0.000	N	-	[001]
Ti_2SeTeS	4.68	FM	1.0	0.000	N	-	[001]
$Ti_2SeTeSe$	4.72	AFM Zigzag-X	0.9	0.877	N	-	[100]
$Ti_2SeTeTe$	4.76	AFM Zigzag-X	1.0	0.645	N	-	[110]
Ti_2NPO	4.07	AFM Zigzag-Y	0.3	0.287	N	-	[001]
Ti_2NPS	4.21	AFM Zigzag-Y	0.6	0.674	N	-	[001]
Ti_2NPSe	4.21	AFM Zigzag-Y	0.7	0.610	N	-	[001]
Ti_2NPTe	4.21	AFM Zigzag-X	0.7	0.532	N	-	[100]
Ti_2NAsO	4.11	AM	0.6	0.000	N	-	[001]
Ti_2NAsS	4.36	FM	0.9	0.000	N	-	[001]
Ti_2NAsSe	4.55	FM	0.9	0.000	N	-	[100]
Ti_2NAsTe	4.47	FM	1.4	0.000	N	-	[100]
Ti_2PAsO	4.12	NM	0.0	0.000	N	-	-
Ti_2PAsS	4.63	FM	0.8	0.000	N	-	[100]
Ti_2PAsSe	4.84	AFM Stripe	1.0	0.338	N	-	[100]
Ti_2PAsTe	4.78	AFM Zigzag-X	1.0	0.375	N	-	[001]
V_2FClO	4.07	AM	2.6	1.810	N	-	[001]
V_2FClS	4.39	AM	2.6	1.251	N	-	[001]
V_2FClSe	4.43	AM	2.6	0.856	N	-	[001]
V_2FClTe	4.46	AM	2.6	0.303	N	-	[100]
V_2FBrO	4.09	AM	2.6	1.814	N	-	[001]
V_2FBrS	4.42	AM	2.6	1.288	N	-	[001]
V_2FBrSe	4.47	AM	2.6	0.914	N	-	[001]
V_2FBrTe	4.50	AM	2.6	0.371	N	-	[110]
V_2FIO	4.11	AM	2.6	1.682	N	-	[001]
V_2FIS	4.47	AM	2.6	1.331	N	-	[001]

V_2FISe	4.52	AM	2.6	0.964	N	-	[001]
V_2FITe	4.56	AM	2.6	0.444	N	-	[001]
V_2ClBrO	4.16	AM	2.6	1.710	N	-	[001]
V_2ClBrS	4.62	AM	2.6	1.903	N	-	[001]
$V_2ClBrSe$	4.94	AFM Stripe	2.7	1.564	N	-	[001]
$V_2ClBrTe$	5.26	AFM Stripe	2.7	1.297	N	-	[001]
V_2ClIO	4.18	AM	2.6	1.588	N	-	[001]
V_2ClIS	4.65	AM	2.6	1.917	N	-	[001]
V_2ClISe	4.77	AM	2.6	1.591	N	-	[001]
V_2ClITe	4.96	AM	2.7	0.934	N	-	[001]
V_2BrIO	4.20	AM	2.6	1.562	N	-	[001]
V_2BrIS	4.70	AM	2.6	1.908	N	-	[001]
V_2BrISe	4.84	AM	2.6	1.717	N	-	[001]
V_2BrITe	5.06	AM	2.7	1.118	N	-	[001]
V_2OSO	3.85	AM	1.7	0.772	N	-	[100]
V_2OSS	4.57	AM	2.6	0.439	N	-	[001]
V_2OSSe	4.60	AM	2.6	0.212	N	-	[001]
V_2OSTe	3.90	AM	1.9	0.000	N	-	[110]
V_2OSeO	3.88	AM	1.8	0.664	N	-	[100]
V_2OSeS	3.94	AM	1.7	0.000	N	-	[001]
V_2OSeSe	3.95	AM	1.8	0.000	N	-	[001]
V_2OSeTe	3.94	AM	1.9	0.000	N	-	[100]
V_2OTeO	3.91	AM	1.8	0.256	N	-	[001]
V_2OTeS	3.99	AM	1.8	0.000	N	-	[001]
V_2OTeSe	4.00	AM	1.8	0.000	N	-	[001]
V_2OTeTe	3.99	AM	1.9	0.000	N	-	[100]
V_2SSeO	4.01	AM	1.8	0.427	N	-	[100]
V_2SSeS	4.31	FM	2.1	0.000	N	-	[001]
V_2SSeSe	4.99	AM	2.7	0.771	N	-	[001]
V_2SSeTe	5.16	AM	2.7	0.568	N	-	[001]
V_2STeO	4.03	AM	1.9	0.000	Y	M-X, M-Y	[001]
V_2STeS	4.36	AM	1.9	0.336	N	-	[100]
V_2STeSe	4.93	AM	2.6	0.189	N	-	[001]
V_2STeTe	5.24	AM	2.7	0.501	N	-	[001]
V_2SeTeO	4.06	AM	1.9	0.000	Y	M-X, M-Y	[001]
V_2SeTeS	4.45	FM	2.1	0.213	N	-	[001]
$V_2SeTeSe$	4.95	FM	2.6	0.295	N	-	[100]
$V_2SeTeTe$	5.22	AM	2.7	0.199	N	-	[001]
V_2NPO	4.07	AM	1.9	0.224	N	-	[001]
V_2NPS	4.07	AFM Zigzag-Y	1.8	0.593	N	-	[100]
V_2NPSe	4.07	AFM Zigzag-Y	1.9	0.557	N	-	[001]
V_2NPTe	4.08	AFM Zigzag-Y	1.9	0.580	N	-	[001]
V_2NAsO	4.06	AM	1.9	0.304	N	-	[001]
V_2NAsS	4.07	AFM Zigzag-Y	1.8	0.605	N	-	[100]
V_2NAsSe	4.07	AFM Zigzag-Y	1.9	0.568	N	-	[100]

V_2NAsTe	4.40	AFM Zigzag-X	2.6	0.000	N	-	[001]
V_2PAsO	4.21	AM	1.9	0.000	N	-	[001]
V_2PAsS	4.57	AFM Zigzag-Y	2.1	0.000	N	-	[100]
V_2PAsSe	4.63	AFM Zigzag-Y	2.2	0.471	N	-	[110]
V_2PAsTe	4.72	AFM Zigzag-Y	2.4	0.086	N	-	[110]
Cr_2FClO	4.03	AM	3.6	1.140	N	-	[001]
Cr_2FClS	4.38	AM	3.7	0.831	N	-	[001]
Cr_2FClSe	4.44	AM	3.7	0.281	N	-	[001]
Cr_2FClTe	4.36	FM	3.9	0.000	N	-	[001]
Cr_2FBrO	4.04	AM	3.6	1.076	N	-	[001]
Cr_2FBrS	4.41	AM	3.7	0.834	N	-	[001]
Cr_2FBrSe	4.47	AM	3.7	0.288	N	-	[001]
Cr_2FBrTe	4.38	FM	3.9	0.000	N	-	[001]
Cr_2FIO	4.06	AM	3.6	0.795	N	-	[100]
Cr_2FIS	4.44	AM	3.7	0.708	N	-	[001]
Cr_2FISe	4.51	AM	3.7	0.293	N	-	[001]
Cr_2FITe	4.42	FM	3.9	0.000	N	-	[100]
Cr_2ClBrO	4.08	AM	3.6	0.987	N	-	[001]
Cr_2ClBrS	4.58	AM	3.7	0.648	N	-	[001]
$Cr_2ClBrSe$	4.71	AM	3.8	0.763	N	-	[001]
$Cr_2ClBrTe$	4.89	AM	3.8	0.147	N	-	[001]
Cr_2ClIO	4.10	AM	3.6	0.760	N	-	[100]
Cr_2ClIS	4.60	AM	3.7	0.547	N	-	[100]
Cr_2ClISe	4.74	AM	3.8	0.662	N	-	[001]
Cr_2ClITe	4.93	AM	3.8	0.183	N	-	[001]
Cr_2BrIO	4.12	AM	3.6	0.750	N	-	[001]
Cr_2BrIS	4.65	AM	3.7	0.483	N	-	[001]
Cr_2BrISe	4.79	AM	3.8	0.587	N	-	[001]
Cr_2BrITe	5.02	AM	3.8	0.348	N	-	[001]
Cr_2OSO	3.81	AM	2.9	1.183	N	-	[001]
Cr_2OSS	4.51	AM	3.7	0.282	N	-	[001]
Cr_2OSSe	4.54	AM	3.7	0.000	N	-	[001]
Cr_2OSTe	4.28	AM	3.7	0.000	Y	Γ-X, Γ-Y	[110]
Cr_2OSeO	3.83	AM	2.9	1.151	N	-	[001]
Cr_2OSeS	4.01	AM	3.0	0.000	Y	Γ-X, Γ-Y	[001]
Cr_2OSeSe	4.28	AM	3.6	0.023	N	-	[100]
Cr_2OSeTe	4.29	AM	3.6	0.000	Y	Γ-X, Γ-Y	[110]
Cr_2OTeO	3.88	AM	3.0	0.300	N	-	[001]
Cr_2OTeS	4.29	AM	3.6	0.000	N	-	[110]
Cr_2OTeSe	4.29	AM	3.6	0.000	N	-	[110]
Cr_2OTeTe	3.92	AM	3.4	0.000	N	-	[001]
Cr_2SSeO	3.96	AM	3.0	0.965	N	-	[001]
Cr_2SSeS	4.34	AM	3.2	0.000	Y	Γ-X, Γ-Y	[001]
Cr_2SSeSe	4.81	AM	3.7	0.000	Y	Γ-X, Γ-Y	[001]
Cr_2SSeTe	4.95	AM	3.8	0.000	N	-	[001]

Cr_2STeO	4.00	AM	3.0	0.444	N	-	[001]
Cr_2STeS	4.44	AM	3.3	0.000	Y	Γ-X, Γ-Y	[001]
Cr_2STeSe	4.57	AM	3.4	0.000	Y	Γ-X, Γ-Y	[001]
Cr_2STeTe	4.82	AM	3.8	0.000	N	-	[110]
Cr_2SeTeO	4.03	AM	3.0	0.376	N	-	[001]
Cr_2SeTeS	4.49	AM	3.3	0.000	Y	Γ-X, Γ-Y	[001]
$Cr_2SeTeSe$	4.56	AM	3.4	0.000	Y	Γ-X, Γ-Y	[001]
$Cr_2SeTeTe$	4.49	AM	3.7	0.000	N	-	[001]
Cr_2NPO	4.04	AM	3.1	0.647	N	-	[001]
Cr_2NPS	4.41	AM	3.3	0.000	N	-	[001]
Cr_2NPSe	4.41	AM	3.5	0.000	N	-	[001]
Cr_2NPTe	4.09	AM	3.4	0.000	N	-	[001]
Cr_2NAsO	4.04	AM	3.1	0.436	N	-	[001]
Cr_2NAsS	4.40	AM	3.3	0.051	N	-	[001]
Cr_2NAsSe	4.13	AFM Stripe	3.8	0.000	N	-	[110]
Cr_2NAsTe	4.14	AM	3.8	0.000	N	-	[001]
Cr_2PAsO	4.20	AM	3.1	0.000	N	-	[001]
Cr_2PAsS	4.62	AM	3.3	0.000	N	-	[001]
Cr_2PAsSe	4.65	AM	3.4	0.000	N	-	[001]
Cr_2PAsTe	4.68	AFM Zigzag-Y	3.7	0.000	N	-	[001]
Mn_2FClO	4.11	AM	4.6	2.799	N	-	[001]
Mn_2FClS	4.44	AM	4.6	3.068	N	-	[001]
Mn_2FClSe	4.49	AM	4.6	2.813	N	-	[001]
Mn_2FClTe	4.55	AM	4.6	2.031	N	-	[001]
Mn_2FBrO	4.13	AM	4.6	2.816	N	-	[001]
Mn_2FBrS	4.46	AM	4.6	3.091	N	-	[001]
Mn_2FBrSe	4.51	AM	4.6	2.859	N	-	[001]
Mn_2FBrTe	4.57	AM	4.6	2.097	N	-	[001]
Mn_2FIO	4.15	AM	4.6	2.934	N	-	[001]
Mn_2FIS	4.49	AM	4.6	3.281	N	-	[001]
Mn_2FISe	4.54	AM	4.6	3.074	N	-	[001]
Mn_2FITe	4.61	AM	4.6	2.310	N	-	[001]
Mn_2ClBrO	4.19	AM	4.6	3.090	N	-	[001]
Mn_2ClBrS	4.73	AM	4.6	2.519	N	-	[001]
$Mn_2ClBrSe$	4.82	AM	4.6	2.234	N	-	[001]
$Mn_2ClBrTe$	4.98	AM	4.6	1.720	N	-	[001]
Mn_2ClIO	4.22	AM	4.6	3.035	N	-	[001]
Mn_2ClIS	4.74	AM	4.6	2.519	N	-	[001]
Mn_2ClISe	4.86	AM	4.6	2.224	N	-	[001]
Mn_2ClITe	5.01	AM	4.6	1.658	N	-	[001]
Mn_2BrIO	4.23	AM	4.6	2.840	N	-	[001]
Mn_2BrIS	4.80	AM	4.6	2.287	N	-	[001]
Mn_2BrISe	4.92	AM	4.6	1.942	N	-	[001]
Mn_2BrITe	5.11	AM	4.6	1.310	N	-	[001]
Mn_2OSO	3.96	FM	4.3	0.000	N	-	[001]

Mn_2OSS	4.57	AFM Stripe	4.5	2.142	N	-	[001]
Mn_2OSSe	4.60	AFM Stripe	4.5	1.993	N	-	[001]
Mn_2OSTe	4.64	AFM Stripe	4.5	1.804	N	-	[001]
Mn_2OSeO	3.97	FM	4.3	0.000	N	-	[100]
Mn_2OSeS	4.56	AFM Stripe	4.5	1.536	N	-	[001]
Mn_2OSeSe	4.58	AFM Stripe	4.5	1.503	N	-	[001]
Mn_2OSeTe	4.62	AM	4.6	1.150	N	-	[001]
Mn_2OTeO	3.98	AM	4.3	0.000	N	-	[001]
Mn_2OTeS	4.56	AFM Stripe	4.5	0.538	N	-	[001]
Mn_2OTeSe	4.59	AFM Stripe	4.5	0.557	N	-	[001]
Mn_2OTeTe	4.63	AM	4.6	0.457	N	-	[001]
Mn_2SSeO	4.08	FM	4.2	0.000	N	-	[100]
Mn_2SSeS	4.97	AFM Zigzag-Y	4.5	1.835	N	-	[001]
Mn_2SSeSe	5.07	AFM Zigzag-Y	4.5	1.854	N	-	[001]
Mn_2SSeTe	5.19	AFM Zigzag-Y	4.5	1.282	N	-	[001]
Mn_2STeO	4.06	AM	4.2	0.000	N	-	[001]
Mn_2STeS	5.00	AFM Zigzag-Y	4.5	1.381	N	-	[110]
Mn_2STeSe	5.09	AFM Zigzag-Y	4.5	1.452	N	-	[001]
Mn_2STeTe	5.21	AFM Zigzag-Y	4.5	1.195	N	-	[001]
Mn_2SeTeO	4.10	FM	4.3	0.000	N	-	[100]
Mn_2SeTeS	5.08	AFM Zigzag-Y	4.5	1.104	N	-	[001]
$Mn_2SeTeSe$	5.19	AFM Zigzag-Y	4.5	1.253	N	-	[001]
$Mn_2SeTeTe$	5.35	AFM Zigzag-Y	4.5	0.867	N	-	[001]
Mn_2NPO	4.18	FM	4.3	0.743	N	-	[100]
Mn_2NPS	4.26	AM	4.3	0.415	N	-	[001]
Mn_2NPSe	4.27	AM	4.3	0.402	N	-	[001]
Mn_2NPTe	4.40	AM	4.4	0.000	N	-	[100]
Mn_2NAsO	4.15	FM	4.3	0.283	N	-	[100]
Mn_2NAsS	4.22	AM	4.3	0.000	N	-	[001]
Mn_2NAsSe	4.41	AM	4.4	0.000	N	-	[100]
Mn_2NAsTe	4.29	AM	4.5	0.000	N	-	[100]
Mn_2PAsO	4.40	FM	4.2	0.000	N	-	[100]
Mn_2PAsS	4.73	AM	4.3	0.000	N	-	[001]
Mn_2PAsSe	4.69	AM	4.3	0.000	N	-	[100]
Mn_2PAsTe	4.77	AM	4.4	0.000	N	-	[110]
Fe_2FClO	4.01	AFM Stripe	3.7	2.507	N	-	[001]
Fe_2FClS	4.42	AFM Stripe	3.7	2.638	N	-	[001]
Fe_2FClSe	4.59	AFM Stripe	3.7	2.533	N	-	[001]
Fe_2FClTe	4.48	AFM Stripe	3.6	1.840	N	-	[001]
Fe_2FBrO	4.02	AFM Stripe	3.7	2.676	N	-	[001]
Fe_2FBrS	4.43	AFM Stripe	3.7	2.628	N	-	[001]
Fe_2FBrSe	4.25	AFM Stripe	3.6	2.179	N	-	[001]
Fe_2FBrTe	4.50	AFM Stripe	3.6	1.935	N	-	[001]
Fe_2FIO	4.03	AFM Stripe	3.7	2.708	N	-	[001]
Fe_2FIS	4.46	AFM Stripe	3.7	2.612	N	-	[001]

Fe_2FISe	4.41	AFM Stripe	3.7	2.388	N	-	[001]
Fe_2FITe	4.54	AFM Stripe	3.6	1.966	N	-	[001]
Fe_2ClBrO	4.05	AM	3.7	2.922	N	-	[100]
Fe_2ClBrS	4.53	AFM Stripe	3.6	2.418	N	-	[001]
$Fe_2ClBrSe$	4.73	AFM Stripe	3.7	2.109	N	-	[001]
$Fe_2ClBrTe$	4.89	AFM Stripe	3.7	1.535	N	-	[001]
Fe_2ClIO	4.08	AM	3.7	2.676	N	-	[001]
Fe_2ClIS	4.64	AFM Stripe	3.6	2.407	N	-	[001]
Fe_2ClISe	4.75	AFM Stripe	3.7	1.994	N	-	[001]
Fe_2ClITe	4.93	AFM Stripe	3.7	1.384	N	-	[001]
Fe_2BrIO	4.10	AM	3.7	2.646	N	-	[001]
Fe_2BrIS	4.69	AFM Stripe	3.6	2.348	N	-	[001]
Fe_2BrISe	4.82	AFM Stripe	3.6	1.956	N	-	[001]
Fe_2BrITe	5.01	AFM Stripe	3.6	1.229	N	-	[110]
Fe_2OSO	3.88	AFM Stripe	4.0	0.637	N	-	[110]
Fe_2OSS	4.44	AFM Stripe	3.6	1.969	N	-	[001]
Fe_2OSSe	4.51	AFM Stripe	3.6	1.948	N	-	[001]
Fe_2OSTe	4.53	AFM Zigzag-X	3.6	1.372	N	-	[001]
Fe_2OSeO	3.91	AFM Stripe	4.0	0.469	N	-	[110]
Fe_2OSeS	4.40	AFM Zigzag-X	3.7	1.137	N	-	[100]
Fe_2OSeSe	4.48	AFM Zigzag-X	3.7	1.214	N	-	[100]
Fe_2OSeTe	4.50	AFM Stripe	3.6	1.645	N	-	[001]
Fe_2OTeO	3.95	AFM Stripe	3.9	0.000	N	-	[110]
Fe_2OTeS	4.40	AFM Zigzag-X	3.6	0.280	N	-	[001]
Fe_2OTeSe	4.49	AFM Zigzag-X	3.6	0.431	N	-	[001]
Fe_2OTeTe	4.50	AFM Stripe	3.6	1.005	N	-	[001]
Fe_2SSeO	4.03	AFM Stripe	3.9	0.000	N	-	[110]
Fe_2SSeS	4.83	AFM Stripe	3.6	1.590	N	-	[001]
Fe_2SSeSe	4.97	AFM Stripe	3.6	1.588	N	-	[001]
Fe_2SSeTe	5.10	AFM Stripe	3.6	0.963	N	-	[001]
Fe_2STeO	4.06	AFM Stripe	3.8	0.000	N	-	[110]
Fe_2STeS	4.89	AFM Stripe	3.6	1.329	N	-	[001]
Fe_2STeSe	5.00	AFM Stripe	3.6	1.317	N	-	[001]
Fe_2STeTe	5.13	AFM Stripe	3.6	0.818	N	-	[001]
Fe_2SeTeO	4.08	AFM Stripe	3.8	0.000	Y	Γ-X	[110]
Fe_2SeTeS	4.95	AFM Stripe	3.6	0.983	N	-	[001]
$Fe_2SeTeSe$	5.08	AFM Stripe	3.6	1.072	N	-	[001]
$Fe_2SeTeTe$	5.25	AFM Stripe	3.6	0.551	N	-	[001]
Fe_2NPO	4.11	AFM Stripe	3.7	1.173	N	-	[110]
Fe_2NPS	4.16	AM	3.4	0.035	N	-	[100]
Fe_2NPSe	4.15	AFM Zigzag-Y	3.5	0.000	N	-	[001]
Fe_2NPTe	4.13	AM	3.3	0.000	N	-	[100]
Fe_2NAsO	4.03	AFM Stripe	3.6	0.000	Y	Γ-X	[110]
Fe_2NAsS	3.95	AFM Zigzag-Y	3.5	0.000	N	-	[001]
Fe_2NAsSe	4.22	AM	3.4	0.000	N	-	[100]

Fe_2NAsTe	3.92	AM	3.4	0.000	Y	Γ-X, Γ-Y	[110]
Fe_2PAsO	4.27	AFM Stripe	3.7	0.000	Y	Γ-X	[100]
Fe_2PAsS	4.75	AFM Stripe	3.5	0.000	Y	Γ-X	[110]
Fe_2PAsSe	4.58	AFM Zigzag-Y	3.4	0.000	N	-	[100]
Fe_2PAsTe	4.55	AFM Zigzag-Y	3.2	0.000	N	-	[100]
Co_2FClO	3.92	AFM Stripe	2.7	3.286	N	-	[100]
Co_2FClS	4.31	AFM Zigzag-Y	2.7	2.298	N	-	[001]
Co_2FClSe	4.27	AFM Stripe	2.6	1.778	N	-	[100]
Co_2FClTe	4.35	AFM Stripe	2.6	1.467	N	-	[110]
Co_2FBrO	3.94	AFM Stripe	2.7	3.232	N	-	[100]
Co_2FBrS	4.31	AFM Zigzag-Y	2.7	2.225	N	-	[001]
Co_2FBrSe	4.51	AFM Zigzag-Y	2.7	2.077	N	-	[001]
Co_2FBrTe	4.38	AFM Stripe	2.6	1.521	N	-	[110]
Co_2FIO	3.97	AFM Stripe	2.7	2.617	N	-	[001]
Co_2FIS	4.35	AFM Zigzag-Y	2.7	2.058	N	-	[001]
Co_2FISe	4.35	AFM Stripe	1.1	0.021	N	-	[001]
Co_2FITe	4.43	AFM Stripe	2.6	1.573	N	-	[110]
Co_2ClBrO	4.00	AFM Zigzag-X	2.7	2.910	N	-	[001]
Co_2ClBrS	4.47	AM	2.7	1.341	N	-	[001]
$Co_2ClBrSe$	4.61	AFM Zigzag-X	2.6	1.901	N	-	[100]
$Co_2ClBrTe$	4.76	AFM Zigzag-X	2.6	1.192	N	-	[110]
Co_2ClIO	4.03	AFM Zigzag-X	2.7	2.542	N	-	[100]
Co_2ClIS	4.55	AFM Zigzag-X	2.6	2.087	N	-	[100]
Co_2ClISe	4.67	AFM Zigzag-X	2.6	1.885	N	-	[110]
Co_2ClITe	4.80	AFM Zigzag-X	2.6	1.363	N	-	[110]
Co_2BrIO	4.05	AFM Zigzag-X	2.6	2.566	N	-	[100]
Co_2BrIS	4.60	AFM Zigzag-X	2.6	1.988	N	-	[100]
Co_2BrISe	4.72	AFM Zigzag-X	2.6	1.721	N	-	[100]
Co_2BrITe	4.89	AFM Zigzag-X	2.6	1.273	N	-	[110]
Co_2OSO	4.11	AFM Stripe	2.6	1.343	N	-	[100]
Co_2OSS	4.41	FM	2.7	1.688	N	-	[100]
Co_2OSSe	4.45	AFM Zigzag-X	2.6	1.779	N	-	[100]
Co_2OSTe	4.50	AFM Stripe	2.5	1.720	N	-	[100]
Co_2OSeO	4.10	FM	2.7	0.421	N	-	[100]
Co_2OSeS	4.40	AFM Stripe	2.6	1.403	N	-	[001]
Co_2OSeSe	4.42	AFM Zigzag-X	2.6	1.544	N	-	[001]
Co_2OSeTe	4.44	AFM Stripe	2.6	1.587	N	-	[001]
Co_2OTeO	3.88	AFM Zigzag-X	2.7	0.000	N	-	[100]
Co_2OTeS	4.36	AFM Stripe	2.6	0.591	N	-	[001]
Co_2OTeSe	4.38	AFM Zigzag-X	2.6	0.663	N	-	[100]
Co_2OTeTe	3.69	AFM Zigzag-Y	2.4	0.000	N	-	[001]
Co_2SSeO	3.95	FM	2.4	0.000	N	-	[001]
Co_2SSeS	4.74	AFM Stripe	2.5	1.385	N	-	[100]
Co_2SSeSe	4.86	AFM Stripe	2.5	1.379	N	-	[100]
Co_2SSeTe	4.97	AFM Stripe	2.5	0.997	N	-	[100]

Co_2STeO	3.96	FM	2.4	0.000	N	-	[001]
Co_2STeS	4.77	AFM Stripe	2.5	1.080	N	-	[001]
Co_2STeSe	4.88	AFM Stripe	2.5	1.211	N	-	[110]
Co_2STeTe	4.99	AFM Stripe	2.5	0.852	N	-	[100]
Co_2SeTeO	3.93	FM	2.5	0.000	N	-	[100]
Co_2SeTeS	4.82	AFM Stripe	2.5	0.813	N	-	[100]
$Co_2SeTeSe$	4.97	AFM Stripe	2.5	0.823	N	-	[100]
$Co_2SeTeTe$	5.08	AFM Stripe	2.5	0.000	N	-	[110]
Co_2NPO	4.03	AFM Stripe	2.3	1.015	N	-	[001]
Co_2NPS	4.26	AFM Stripe	2.0	0.645	N	-	[001]
Co_2NPSe	4.27	AM	2.0	0.191	N	-	[100]
Co_2NPTe	4.21	AM	2.0	0.256	N	-	[100]
Co_2NAsO	4.01	FM	2.4	0.354	N	-	[100]
Co_2NAsS	4.20	AM	2.1	0.099	N	-	[001]
Co_2NAsSe	4.16	AM	2.0	0.000	N	-	[001]
Co_2NAsTe	4.31	AM	2.0	0.259	N	-	[100]
Co_2PAsO	4.23	FM	2.2	0.000	N	-	[001]
Co_2PAsS	4.38	AM	2.2	0.000	N	-	[100]
Co_2PAsSe	4.31	AM	2.0	0.000	N	-	[110]
Co_2PAsTe	4.39	AM	2.0	0.000	N	-	[100]
Ni_2FClO	3.84	AFM Stripe	1.7	2.256	N	-	[001]
Ni_2FClS	4.21	AFM Zigzag-Y	1.7	1.576	N	-	[001]
Ni_2FClSe	4.32	AFM Zigzag-Y	1.6	1.196	N	-	[001]
Ni_2FClTe	3.48	FM	1.0	0.000	N	-	[100]
Ni_2FBrO	3.86	AFM Stripe	1.7	2.256	N	-	[100]
Ni_2FBrS	4.24	AM	1.6	1.305	N	-	[001]
Ni_2FBrSe	4.35	AM	1.6	0.938	N	-	[001]
Ni_2FBrTe	3.51	FM	1.0	0.000	N	-	[100]
Ni_2FIO	3.90	AFM Stripe	1.7	2.117	N	-	[100]
Ni_2FIS	4.28	AM	1.6	1.350	N	-	[001]
Ni_2FISe	4.39	AM	1.6	0.966	N	-	[001]
Ni_2FITe	4.12	AFM Zigzag-X	1.3	0.205	N	-	[001]
Ni_2ClBrO	3.93	AFM Zigzag-X	1.7	1.923	N	-	[100]
Ni_2ClBrS	4.39	AFM Stripe	1.6	1.173	N	-	[100]
$Ni_2ClBrSe$	4.53	AFM Zigzag-Y	1.6	0.920	N	-	[100]
$Ni_2ClBrTe$	3.61	AFM Zigzag-X	0.5	0.000	N	-	[001]
Ni_2ClIO	3.97	AFM Zigzag-X	1.6	1.828	N	-	[100]
Ni_2ClIS	4.42	AFM Zigzag-X	1.5	1.004	N	-	[100]
Ni_2ClISe	4.56	AFM Zigzag-Y	1.5	0.849	N	-	[001]
Ni_2ClITe	3.68	FM	0.6	0.000	N	-	[001]
Ni_2BrIO	3.99	AFM Zigzag-X	1.6	1.769	N	-	[100]
Ni_2BrIS	4.45	AFM Zigzag-X	1.5	0.949	N	-	[100]
Ni_2BrISe	4.60	AFM Zigzag-Y	1.5	0.680	N	-	[100]
Ni_2BrITe	3.72	FM	0.6	0.000	N	-	[001]
Ni_2OSO	4.06	AFM Stripe	1.6	1.216	N	-	[001]

Ni_2OSS	4.40	AFM Zigzag-Y	1.5	1.698	N	-	[001]
Ni_2OSSe	4.51	AFM Zigzag-Y	1.5	1.572	N	-	[001]
Ni_2OSTe	4.39	NM	0.0	0.000	N	-	-
Ni_2OSeO	4.05	AFM Stripe	1.6	0.821	N	-	[001]
Ni_2OSeS	4.39	AFM Zigzag-Y	1.5	1.466	N	-	[001]
Ni_2OSeSe	4.41	AFM Zigzag-Y	1.5	1.398	N	-	[001]
Ni_2OSeTe	3.32	FM	1.1	0.000	N	-	[110]
Ni_2OTeO	3.77	AFM Zigzag-Y	1.7	0.000	N	-	[001]
Ni_2OTeS	4.28	FM	1.5	0.136	N	-	[100]
Ni_2OTeSe	4.45	AFM Zigzag-Y	1.1	0.993	N	-	[110]
Ni_2OTeTe	4.64	AFM Zigzag-Y	1.0	0.332	N	-	[110]
Ni_2SSeO	3.88	AM	1.0	0.000	N	-	[100]
Ni_2SSeS	4.56	AFM Stripe	1.4	0.999	N	-	[110]
Ni_2SSeSe	4.73	AFM Zigzag-Y	1.4	0.805	N	-	[110]
Ni_2SSeTe	3.56	FM	0.5	0.000	N	-	[100]
Ni_2STeO	3.91	AM	1.0	0.000	N	-	[001]
Ni_2STeS	4.54	AFM Stripe	1.3	0.663	N	-	[110]
Ni_2STeSe	4.61	AFM Zigzag-Y	1.3	0.555	N	-	[110]
Ni_2STeTe	4.03	AM	0.9	0.000	N	-	[001]
Ni_2SeTeO	3.93	AM	1.0	0.000	N	-	[001]
Ni_2SeTeS	4.60	AFM Stripe	1.2	0.453	N	-	[001]
$Ni_2SeTeSe$	4.19	AM	1.0	0.000	N	-	[110]
$Ni_2SeTeTe$	4.17	AM	0.9	0.000	N	-	[100]
Ni_2NPO	3.93	AM	1.0	0.723	N	-	[100]
Ni_2NPS	3.70	AM	0.7	0.000	N	-	[001]
Ni_2NPSe	4.10	AFM Zigzag-Y	0.9	0.347	N	-	[001]
Ni_2NPTe	3.69	AM	0.6	0.000	N	-	[100]
Ni_2NAsO	3.73	AFM Stripe	0.5	0.769	N	-	[110]
Ni_2NAsS	3.67	AM	0.6	0.000	N	-	[001]
Ni_2NAsSe	3.68	AM	0.6	0.000	N	-	[100]
Ni_2NAsTe	3.67	AM	0.5	0.000	N	-	[110]
Ni_2PAsO	4.13	FM	0.8	0.000	N	-	[001]
Ni_2PAsS	4.10	AM	0.6	0.000	N	-	[001]
Ni_2PAsSe	4.15	AFM Zigzag-X	0.4	0.000	N	-	[001]
Ni_2PAsTe	4.21	NM	0.0	0.000	N	-	-
Cu_2FClO	3.82	AFM Stripe	0.7	1.391	N	-	[001]
Cu_2FClS	4.17	AFM Zigzag-Y	0.4	0.377	N	-	[001]
Cu_2FClSe	3.73	NM	0.0	0.000	N	-	-
Cu_2FClTe	3.62	NM	0.0	0.000	N	-	-
Cu_2FBrO	3.84	AFM Zigzag-X	0.6	0.952	N	-	[100]
Cu_2FBrS	4.20	AFM Zigzag-Y	0.4	0.223	N	-	[001]
Cu_2FBrSe	4.30	FM	0.3	0.000	N	-	[001]
Cu_2FBrTe	3.67	NM	0.0	0.000	N	-	-
Cu_2FIO	3.87	AFM Zigzag-X	0.6	0.000	N	-	[110]
Cu_2FIS	4.26	AFM Zigzag-Y	0.3	0.000	N	-	[001]

Cu_2FISe	4.39	FM	0.3	0.000	N	-	[001]
Cu_2FITe	3.77	NM	0.0	0.000	N	-	-
Cu_2ClBrO	3.90	AFM Zigzag-X	0.6	0.998	N	-	[100]
Cu_2ClBrS	4.33	FM	0.2	0.000	N	-	[001]
$Cu_2ClBrSe$	4.07	NM	0.0	0.000	N	-	-
$Cu_2ClBrTe$	3.76	NM	0.0	0.000	N	-	-
Cu_2ClIO	3.89	AFM Zigzag-X	0.5	0.000	N	-	[110]
Cu_2ClIS	4.37	AFM Zigzag-X	0.1	0.000	N	-	[001]
Cu_2ClISe	4.49	FM	0.1	0.000	N	-	[001]
Cu_2ClITe	3.85	NM	0.0	0.000	N	-	-
Cu_2BrIO	3.90	AFM Zigzag-X	0.4	0.000	N	-	[001]
Cu_2BrIS	4.38	AFM Zigzag-X	0.1	0.000	N	-	[001]
Cu_2BrISe	4.21	NM	0.0	0.000	N	-	-
Cu_2BrITe	4.36	NM	0.0	0.000	N	-	-
Cu_2OSO	4.11	NM	0.0	0.000	N	-	-
Cu_2OSS	4.53	FM	0.2	0.000	N	-	[100]
Cu_2OSSe	4.67	NM	0.0	0.000	N	-	-
Cu_2OSTe	4.96	NM	0.0	0.000	N	-	-
Cu_2OSeO	4.10	NM	0.0	0.000	N	-	-
Cu_2OSeS	4.49	FM	0.2	0.000	N	-	[100]
Cu_2OSeSe	4.64	FM	0.2	0.000	N	-	[100]
Cu_2OSeTe	4.91	NM	0.0	0.000	N	-	-
Cu_2OTeO	3.67	FM	0.3	0.000	N	-	[100]
Cu_2OTeS	3.64	NM	0.0	0.000	N	-	-
Cu_2OTeSe	4.59	NM	0.0	0.000	N	-	-
Cu_2OTeTe	4.87	NM	0.0	0.000	N	-	-
Cu_2SSeO	4.20	NM	0.0	0.000	N	-	-
Cu_2SSeS	4.65	FM	0.1	0.000	N	-	[100]
Cu_2SSeSe	4.78	NM	0.0	0.000	N	-	-
Cu_2SSeTe	4.55	NM	0.0	0.000	N	-	-
Cu_2STeO	3.93	AFM Zigzag-X	0.3	0.000	N	-	[110]
Cu_2STeS	4.69	NM	0.0	0.000	N	-	-
Cu_2STeSe	4.79	NM	0.0	0.000	N	-	-
Cu_2STeTe	5.07	NM	0.0	0.000	N	-	-
Cu_2SeTeO	3.94	AFM Zigzag-X	0.2	0.000	N	-	[110]
Cu_2SeTeS	4.73	NM	0.0	0.000	N	-	-
$Cu_2SeTeSe$	4.88	NM	0.0	0.000	N	-	-
$Cu_2SeTeTe$	5.11	NM	0.0	0.000	N	-	-
Cu_2NPO	3.73	NM	0.0	0.197	N	-	-
Cu_2NPS	4.44	NM	0.0	0.779	N	-	-
Cu_2NPSe	3.70	NM	0.0	0.000	Y	Γ-X	-
Cu_2NPTe	4.24	NM	0.0	0.461	N	-	-
Cu_2NAsO	3.82	AFM Stripe	0.3	0.452	N	-	[001]
Cu_2NAsS	3.70	NM	0.0	0.480	N	-	-
Cu_2NAsSe	3.76	NM	0.0	0.258	N	-	-

Cu_2NAsTe	3.86	NM	0.0	0.000	N	-	-
Cu_2PAsO	4.24	NM	0.0	0.000	N	-	-
Cu_2PAsS	4.57	NM	0.0	0.187	N	-	-
Cu_2PAsSe	4.15	NM	0.0	0.000	N	-	-
Cu_2PAsTe	4.18	NM	0.0	0.357	N	-	-
Zn_2FClO	3.93	NM	0.0	3.129	N	-	-
Zn_2FClS	4.25	NM	0.0	2.543	N	-	-
Zn_2FClSe	4.34	NM	0.0	2.139	N	-	-
Zn_2FClTe	4.47	NM	0.0	1.105	N	-	-
Zn_2FBrO	3.95	NM	0.0	2.964	N	-	-
Zn_2FBrS	4.27	NM	0.0	2.509	N	-	-
Zn_2FBrSe	4.35	NM	0.0	2.119	N	-	-
Zn_2FBrTe	4.49	NM	0.0	1.073	N	-	-
Zn_2FIO	3.99	NM	0.0	2.056	N	-	-
Zn_2FIS	4.30	NM	0.0	1.989	N	-	-
Zn_2FISe	4.38	NM	0.0	1.894	N	-	-
Zn_2FITe	4.52	NM	0.0	0.964	N	-	-
Zn_2ClBrO	4.03	NM	0.0	3.300	N	-	-
Zn_2ClBrS	4.49	NM	0.0	0.956	N	-	-
$Zn_2ClBrSe$	4.60	NM	0.0	0.512	N	-	-
$Zn_2ClBrTe$	4.72	NM	0.0	0.000	N	-	-
Zn_2ClIO	4.07	NM	0.0	2.797	N	-	-
Zn_2ClIS	4.52	NM	0.0	0.815	N	-	-
Zn_2ClISe	4.63	NM	0.0	0.379	N	-	-
Zn_2ClITe	4.77	NM	0.0	0.000	N	-	-
Zn_2BrIO	4.09	NM	0.0	2.736	N	-	-
Zn_2BrIS	4.57	NM	0.0	0.505	N	-	-
Zn_2BrISe	4.69	NM	0.0	0.063	N	-	-
Zn_2BrITe	4.82	NM	0.0	0.000	N	-	-
Zn_2OSO	4.13	NM	0.0	0.524	N	-	-
Zn_2OSS	4.42	NM	0.0	0.749	N	-	-
Zn_2OSSe	4.50	NM	0.0	0.571	N	-	-
Zn_2OSTe	4.67	NM	0.0	0.000	N	-	-
Zn_2OSeO	4.14	NM	0.0	0.000	N	-	-
Zn_2OSeS	4.40	NM	0.0	0.653	N	-	-
Zn_2OSeSe	4.47	NM	0.0	0.563	N	-	-
Zn_2OSeTe	4.60	NM	0.0	0.180	N	-	-
Zn_2OTeO	3.71	NM	0.0	0.000	N	-	-
Zn_2OTeS	4.41	NM	0.0	0.000	N	-	-
Zn_2OTeSe	4.48	NM	0.0	0.000	N	-	-
Zn_2OTeTe	4.61	NM	0.0	0.000	N	-	-
Zn_2SSeO	4.35	NM	0.0	0.552	N	-	-
Zn_2SSeS	4.80	NM	0.0	0.000	N	-	-
Zn_2SSeSe	4.87	NM	0.0	0.000	N	-	-
Zn_2SSeTe	5.03	NM	0.0	0.000	N	-	-

Zn_2STeO	3.76	NM	0.0	0.000	N	-	-
Zn_2STeS	4.83	NM	0.0	0.000	N	-	-
Zn_2STeSe	4.92	NM	0.0	0.000	N	-	-
Zn_2STeTe	5.07	NM	0.0	0.000	N	-	-
Zn_2SeTeO	3.80	NM	0.0	0.000	N	-	-
Zn_2SeTeS	4.91	NM	0.0	0.000	N	-	-
$Zn_2SeTeSe$	4.99	NM	0.0	0.000	N	-	-
$Zn_2SeTeTe$	5.15	NM	0.0	0.000	N	-	-
Zn_2NPO	3.72	NM	0.0	0.000	N	-	-
Zn_2NPS	3.74	NM	0.0	0.000	N	-	-
Zn_2NPSe	3.78	NM	0.0	0.000	N	-	-
Zn_2NPTe	4.99	NM	0.0	0.000	N	-	-
Zn_2NAsO	3.70	NM	0.0	0.000	N	-	-
Zn_2NAsS	4.60	NM	0.0	0.000	N	-	-
Zn_2NAsSe	4.82	NM	0.0	0.000	N	-	-
Zn_2NAsTe	3.86	NM	0.0	0.000	N	-	-
Zn_2PAsO	4.34	NM	0.0	0.000	N	-	-
Zn_2PAsS	4.82	NM	0.0	0.000	N	-	-
Zn_2PAsSe	4.93	NM	0.0	0.000	N	-	-
Zn_2PAsTe	4.61	NM	0.0	0.000	N	-	-
Y_2FClO	4.66	FM	0.1	0.000	N	-	[001]
Y_2FClS	4.94	FM	0.2	0.000	N	-	[001]
Y_2FClSe	4.95	FM	0.3	0.000	N	-	[001]
Y_2FClTe	5.01	FM	0.5	0.000	N	-	[001]
Y_2FBrO	4.69	FM	0.1	0.000	N	-	[001]
Y_2FBrS	5.01	AFM Zigzag-Y	0.1	0.000	N	-	[001]
Y_2FBrSe	5.01	FM	0.2	0.000	N	-	[001]
Y_2FBrTe	5.09	FM	0.5	0.000	N	-	[001]
Y_2FIO	4.71	NM	0.0	0.000	N	-	-
Y_2FIS	5.07	NM	0.0	0.000	N	-	-
Y_2FISe	5.10	FM	0.2	0.000	N	-	[100]
Y_2FITe	5.16	FM	0.4	0.000	N	-	[001]
Y_2ClBrO	4.76	FM	0.3	0.000	N	-	[100]
Y_2ClBrS	5.10	NM	0.0	0.000	N	-	-
$Y_2ClBrSe$	5.16	NM	0.0	0.000	N	-	-
$Y_2ClBrTe$	5.77	AM	0.1	0.000	N	-	[001]
Y_2ClIO	4.75	FM	0.4	0.000	N	-	[100]
Y_2ClIS	5.13	NM	0.0	0.000	N	-	-
Y_2ClISe	5.20	NM	0.0	0.000	N	-	-
Y_2ClITe	5.81	AM	0.2	0.000	N	-	[001]
Y_2BrIO	4.66	FM	0.5	0.000	N	-	[100]
Y_2BrIS	5.20	NM	0.0	0.000	N	-	-
Y_2BrISe	5.27	NM	0.0	0.000	N	-	-
Y_2BrITe	5.46	NM	0.0	0.000	N	-	-
Y_2OSO	4.54	NM	0.0	0.897	N	-	-

Y_2OSS	4.73	NM	0.0	0.171	N	-	-
Y_2OSSe	4.92	NM	0.0	0.127	N	-	-
Y_2OSTe	4.93	NM	0.0	0.301	N	-	-
Y_2OSeO	4.56	NM	0.0	0.933	N	-	-
Y_2OSeS	4.77	NM	0.0	0.191	N	-	-
Y_2OSeSe	4.99	NM	0.0	0.113	N	-	-
Y_2OSeTe	4.62	NM	0.0	0.000	N	-	-
Y_2OTeO	4.60	NM	0.0	0.914	N	-	-
Y_2OTeS	4.81	NM	0.0	0.162	N	-	-
Y_2OTeSe	5.09	NM	0.0	0.077	N	-	-
Y_2OTeTe	4.68	NM	0.0	0.000	N	-	-
Y_2SSeO	4.72	NM	0.0	1.082	N	-	-
Y_2SSeS	5.13	NM	0.0	0.123	N	-	-
Y_2SSeSe	5.37	NM	0.0	0.000	N	-	-
Y_2SSeTe	5.24	NM	0.0	0.236	N	-	-
Y_2STeO	4.76	NM	0.0	1.100	N	-	-
Y_2STeS	5.18	NM	0.0	0.233	N	-	-
Y_2STeSe	5.22	NM	0.0	0.343	N	-	-
Y_2STeTe	5.38	NM	0.0	0.222	N	-	-
Y_2SeTeO	4.79	NM	0.0	1.118	N	-	-
Y_2SeTeS	5.26	NM	0.0	0.170	N	-	-
Y_2SeTeSe	5.30	NM	0.0	0.297	N	-	-
Y_2SeTeTe	5.44	NM	0.0	0.218	N	-	-
Y_2NPO	4.75	NM	0.0	1.234	N	-	-
Y_2NPS	4.94	NM	0.0	0.661	N	-	-
Y_2NPSe	4.94	NM	0.0	0.730	N	-	-
Y_2NPTe	4.95	NM	0.0	0.623	N	-	-
Y_2NAsO	4.75	NM	0.0	1.437	N	-	-
Y_2NAsS	4.94	NM	0.0	0.837	N	-	-
Y_2NAsSe	4.94	NM	0.0	0.796	N	-	-
Y_2NAsTe	5.19	NM	0.0	0.000	N	-	-
Y_2PAsO	5.02	NM	0.0	1.173	N	-	-
Y_2PAsS	5.41	NM	0.0	0.253	N	-	-
Y_2PAsSe	5.44	NM	0.0	0.510	N	-	-
Y_2PAsTe	5.51	NM	0.0	0.337	N	-	-
Zr_2FClO	4.37	AM	0.9	0.544	N	-	[100]
Zr_2FClS	4.35	NM	0.0	0.000	N	-	-
Zr_2FClSe	4.54	AM	0.2	0.000	N	-	[001]
Zr_2FClTe	4.62	AM	0.2	0.000	N	-	[001]
Zr_2FBrO	4.39	AM	0.9	0.491	N	-	[100]
Zr_2FBrS	4.37	NM	0.0	0.000	N	-	-
Zr_2FBrSe	4.81	AFM Zigzag-Y	1.0	0.156	N	-	[110]
Zr_2FBrTe	4.72	AM	0.3	0.000	N	-	[001]
Zr_2FIO	4.40	AM	0.9	0.186	N	-	[100]
Zr_2FIS	4.40	NM	0.0	0.000	N	-	-

Zr_2FISe	4.43	NM	0.0	0.000	N	-	-
Zr_2FITe	4.85	FM	1.0	0.000	N	-	[100]
Zr_2ClBrO	4.47	AM	0.9	0.349	N	-	[100]
Zr_2ClBrS	4.77	AM	0.8	0.162	N	-	[100]
$Zr_2ClBrSe$	4.88	AM	0.9	0.000	Y	M-X, M-Y	[100]
$Zr_2ClBrTe$	4.98	AM	0.9	0.000	Y	M-X, M-Y	[110]
Zr_2ClIO	4.47	AM	0.9	0.324	N	-	[100]
Zr_2ClIS	4.79	AM	0.8	0.000	Y	M-X, M-Y	[100]
Zr_2ClISe	4.84	AFM Zigzag-X	0.7	0.383	N	-	[001]
Zr_2ClITe	4.77	AFM Stripe	0.5	0.254	N	-	[001]
Zr_2BrIO	4.48	AM	0.8	0.228	N	-	[100]
Zr_2BrIS	4.71	AFM Zigzag-X	0.6	0.226	N	-	[001]
Zr_2BrISe	4.92	AM	0.8	0.000	Y	M-X, M-Y	[100]
Zr_2BrITe	5.03	AFM Zigzag-Y	0.8	0.243	N	-	[110]
Zr_2OSO	4.18	NM	0.0	0.247	N	-	-
Zr_2OSS	4.34	AFM Zigzag-Y	0.1	0.000	N	-	[100]
Zr_2OSSe	4.33	FM	0.4	0.000	N	-	[100]
Zr_2OSTe	4.49	NM	0.0	0.000	N	-	-
Zr_2OSeO	4.21	NM	0.0	0.264	N	-	-
Zr_2OSeS	4.36	AFM Zigzag-Y	0.1	0.000	N	-	[100]
Zr_2OSeSe	4.36	FM	0.4	0.000	N	-	[100]
Zr_2OSeTe	4.32	NM	0.0	0.000	N	-	-
Zr_2OTeO	4.24	NM	0.0	0.341	N	-	-
Zr_2OTeS	4.41	AFM Zigzag-Y	0.1	0.000	N	-	[100]
Zr_2OTeSe	4.94	NM	0.0	0.151	N	-	-
Zr_2OTeTe	4.55	NM	0.0	0.000	N	-	-
Zr_2SSeO	4.38	NM	0.0	0.000	N	-	-
Zr_2SSeS	4.66	NM	0.0	0.090	N	-	-
Zr_2SSeSe	4.72	NM	0.0	0.000	N	-	-
Zr_2SSeTe	4.79	NM	0.0	0.000	N	-	-
Zr_2STeO	4.41	NM	0.0	0.000	N	-	-
Zr_2STeS	4.72	NM	0.0	0.282	N	-	-
Zr_2STeSe	4.75	AFM Zigzag-Y	0.1	0.000	N	-	[001]
Zr_2STeTe	4.88	NM	0.0	0.113	N	-	-
Zr_2SeTeO	4.43	NM	0.0	0.000	N	-	-
Zr_2SeTeS	4.76	NM	0.0	0.000	N	-	-
$Zr_2SeTeSe$	4.82	NM	0.0	0.000	N	-	-
$Zr_2SeTeTe$	4.94	NM	0.0	0.000	N	-	-
Zr_2NPO	4.38	NM	0.0	0.000	N	-	-
Zr_2NPS	4.64	NM	0.0	0.000	N	-	-
Zr_2NPSe	4.56	NM	0.0	0.187	N	-	-
Zr_2NPTe	4.53	FM	0.4	0.000	N	-	[001]
Zr_2NAsO	4.34	NM	0.0	0.000	N	-	-
Zr_2NAsS	4.65	NM	0.0	0.000	N	-	-
Zr_2NAsSe	4.70	NM	0.0	0.000	N	-	-

Zr_2NAsTe	4.76	NM	0.0	0.000	N	-	-
Zr_2PAsO	4.45	NM	0.0	0.000	N	-	-
Zr_2PAsS	4.92	NM	0.0	0.000	N	-	-
Zr_2PAsSe	4.98	NM	0.0	0.303	N	-	-
Zr_2PAsTe	5.05	NM	0.0	0.235	N	-	-
Nb_2FClO	4.22	AM	2.0	1.047	N	-	[001]
Nb_2FClS	4.16	AM	1.1	0.000	N	-	[100]
Nb_2FClSe	4.42	AM	1.5	0.000	Y	Γ-X, Γ-Y	[001]
Nb_2FClTe	4.47	AM	1.4	0.000	N	-	[001]
Nb_2FBrO	4.23	AM	2.0	0.937	N	-	[001]
Nb_2FBrS	4.65	AM	2.0	0.384	N	-	[001]
Nb_2FBrSe	4.73	AM	2.0	0.000	N	-	[001]
Nb_2FBrTe	4.82	AM	2.0	0.000	N	-	[110]
Nb_2FIO	4.23	AM	1.9	0.950	N	-	[001]
Nb_2FIS	4.73	AM	2.0	0.804	N	-	[001]
Nb_2FISe	4.85	AM	2.1	0.481	N	-	[001]
Nb_2FITe	4.99	AM	2.1	0.000	N	-	[001]
Nb_2ClBrO	4.31	AM	2.0	1.120	N	-	[001]
Nb_2ClBrS	4.68	AM	1.9	0.745	N	-	[001]
$Nb_2ClBrSe$	4.78	AM	2.0	0.505	N	-	[001]
$Nb_2ClBrTe$	4.91	AM	2.0	0.197	N	-	[001]
Nb_2ClIO	4.31	AM	1.9	0.930	N	-	[001]
Nb_2ClIS	4.75	AM	2.0	1.053	N	-	[001]
Nb_2ClISe	4.88	AM	2.0	0.825	N	-	[001]
Nb_2ClITe	5.07	AM	2.0	0.361	N	-	[001]
Nb_2BrIO	4.17	AM	1.7	0.480	N	-	[001]
Nb_2BrIS	4.72	AM	1.9	0.734	N	-	[001]
Nb_2BrISe	5.15	AM	2.2	0.626	N	-	[001]
Nb_2BrITe	4.61	AM	1.2	0.000	Y	Γ-X, Γ-Y	[100]
Nb_2OSO	3.99	AFM Stripe	0.7	0.384	N	-	[001]
Nb_2OSS	4.03	NM	0.0	0.000	N	-	-
Nb_2OSSe	4.18	NM	0.0	0.000	N	-	-
Nb_2OSTe	4.14	AM	0.7	0.000	N	-	[001]
Nb_2OSeO	3.95	AFM Stripe	0.4	0.113	N	-	[001]
Nb_2OSeS	4.06	AFM Stripe	0.2	0.074	N	-	[110]
Nb_2OSeSe	4.23	FM	0.1	0.000	N	-	[001]
Nb_2OSeTe	4.22	AM	0.9	0.000	N	-	[100]
Nb_2OTeO	3.99	AFM Stripe	0.2	0.000	Y	Γ-X	[001]
Nb_2OTeS	4.11	AFM Stripe	0.2	0.061	N	-	[110]
Nb_2OTeSe	4.11	NM	0.0	0.000	N	-	-
Nb_2OTeTe	4.36	AM	1.2	0.000	N	-	[100]
Nb_2SSeO	4.20	AM	1.1	0.000	Y	M-X, M-Y	[100]
Nb_2SSeS	4.69	FM	1.2	0.000	N	-	[001]
Nb_2SSeSe	4.41	AFM Stripe	0.7	0.170	N	-	[001]
Nb_2SSeTe	4.50	AM	1.0	0.000	N	-	[001]

Nb_2STeO	4.23	AM	1.0	0.000	Y	M-X, M-Y	[100]
Nb_2STeS	4.39	AFM Stripe	0.5	0.294	N	-	[001]
Nb_2STeSe	4.44	AFM Stripe	0.6	0.478	N	-	[001]
Nb_2STeTe	5.30	FM	1.6	0.000	N	-	[001]
Nb_2SeTeO	4.24	AM	1.1	0.000	Y	M-X, M-Y	[100]
Nb_2SeTeS	4.50	AFM Zigzag-X	0.7	0.462	N	-	[001]
$Nb_2SeTeSe$	4.56	AFM Zigzag-X	0.7	0.458	N	-	[001]
$Nb_2SeTeTe$	4.63	AM	1.0	0.000	N	-	[001]
Nb_2NPO	4.04	NM	0.0	0.000	N	-	-
Nb_2NPS	4.28	AFM Stripe	0.7	0.817	N	-	[110]
Nb_2NPSe	4.26	AFM Zigzag-Y	0.7	0.662	N	-	[110]
Nb_2NPTe	4.21	NM	0.0	0.000	N	-	-
Nb_2NAsO	4.05	NM	0.0	0.000	N	-	-
Nb_2NAsS	4.35	AM	0.7	0.000	N	-	[001]
Nb_2NAsSe	4.20	AFM Zigzag-Y	0.6	0.362	N	-	[001]
Nb_2NAsTe	4.07	NM	0.0	0.000	N	-	-
Nb_2PAsO	4.17	NM	0.0	0.000	N	-	-
Nb_2PAsS	4.67	AFM Zigzag-Y	0.9	0.405	N	-	[001]
Nb_2PAsSe	4.65	AFM Zigzag-Y	0.8	0.376	N	-	[001]
Nb_2PAsTe	4.74	AM	0.9	0.149	N	-	[001]
Mo_2FClO	4.22	AM	3.1	0.770	N	-	[001]
Mo_2FClS	4.02	FM	0.2	0.000	N	-	[100]
Mo_2FClSe	4.04	NM	0.0	0.000	N	-	-
Mo_2FClTe	4.08	NM	0.0	0.000	N	-	-
Mo_2FBrO	4.21	AM	3.0	0.751	N	-	[001]
Mo_2FBrS	4.07	FM	0.3	0.000	N	-	[100]
Mo_2FBrSe	4.07	NM	0.0	0.000	N	-	-
Mo_2FBrTe	4.10	AM	0.1	0.000	N	-	[001]
Mo_2FIO	4.20	AM	3.0	0.602	N	-	[001]
Mo_2FIS	4.12	FM	0.4	0.000	N	-	[100]
Mo_2FISe	4.11	AM	0.2	0.000	N	-	[001]
Mo_2FITe	4.13	AM	0.3	0.000	N	-	[001]
Mo_2ClBrO	4.25	AM	3.0	0.710	N	-	[001]
Mo_2ClBrS	4.23	NM	0.0	0.000	N	-	-
$Mo_2ClBrSe$	4.32	NM	0.0	0.000	N	-	-
$Mo_2ClBrTe$	4.47	AM	2.4	0.000	N	-	[001]
Mo_2ClIO	4.11	AM	2.8	0.470	N	-	[110]
Mo_2ClIS	4.27	NM	0.0	0.000	N	-	-
Mo_2ClISe	4.36	NM	0.0	0.000	N	-	-
Mo_2ClITe	4.47	AM	2.5	0.000	N	-	[100]
Mo_2BrIO	4.14	AM	2.8	0.476	N	-	[110]
Mo_2BrIS	4.31	NM	0.0	0.000	N	-	-
Mo_2BrISe	4.84	AM	3.1	0.038	N	-	[001]
Mo_2BrITe	4.55	AFM Zigzag-X	0.9	0.000	N	-	[001]
Mo_2OSO	3.96	AM	1.9	0.638	N	-	[001]

Mo_2OSS	4.06	AM	1.2	0.000	N	-	[001]
Mo_2OSSe	3.91	NM	0.0	0.217	N	-	-
Mo_2OSTe	4.90	AM	2.8	0.000	Y	Γ-X, Γ-Y	[001]
Mo_2OSeO	3.98	AM	1.9	0.646	N	-	[001]
Mo_2OSeS	4.12	AM	1.3	0.000	N	-	[001]
Mo_2OSeSe	4.18	AM	1.3	0.000	N	-	[001]
Mo_2OSeTe	3.97	NM	0.0	0.260	N	-	-
Mo_2OTeO	4.01	AM	1.9	0.281	N	-	[001]
Mo_2OTeS	4.20	AM	1.4	0.000	N	-	[100]
Mo_2OTeSe	4.28	AM	1.4	0.000	N	-	[100]
Mo_2OTeTe	3.62	NM	0.0	0.000	N	-	-
Mo_2SSeO	4.16	AM	2.1	0.533	N	-	[001]
Mo_2SSeS	4.30	AM	1.6	0.000	Y	Γ-X, Γ-Y	[001]
Mo_2SSeSe	4.31	AM	1.4	0.000	N	-	[001]
Mo_2SSeTe	4.30	AM	0.5	0.000	N	-	[001]
Mo_2STeO	4.14	AM	2.0	0.708	N	-	[001]
Mo_2STeS	4.39	AM	1.7	0.000	Y	Γ-X, Γ-Y	[001]
Mo_2STeSe	4.48	AM	1.7	0.000	Y	Γ-X, Γ-Y	[001]
Mo_2STeTe	4.45	AM	1.3	0.000	N	-	[001]
Mo_2SeTeO	4.17	AM	2.0	0.573	N	-	[001]
Mo_2SeTeS	4.43	AM	1.8	0.000	Y	Γ-X, Γ-Y	[001]
$Mo_2SeTeSe$	4.54	AM	1.9	0.000	Y	Γ-X, Γ-Y	[001]
$Mo_2SeTeTe$	4.48	FM	0.9	0.000	N	-	[100]
Mo_2NPO	3.84	FM	0.2	0.000	N	-	[001]
Mo_2NPS	4.26	AM	1.3	0.000	N	-	[001]
Mo_2NPSe	4.31	AM	1.4	0.000	N	-	[001]
Mo_2NPTe	4.09	NM	0.0	0.000	N	-	-
Mo_2NAsO	3.86	FM	0.1	0.000	N	-	[001]
Mo_2NAsS	4.07	NM	0.0	0.000	N	-	-
Mo_2NAsSe	4.09	NM	0.0	0.000	N	-	-
Mo_2NAsTe	4.13	AFM Zigzag-Y	0.2	0.000	N	-	[001]
Mo_2PAsO	4.03	AM	0.9	0.000	N	-	[100]
Mo_2PAsS	4.37	AM	1.3	0.000	N	-	[001]
Mo_2PAsSe	4.46	AM	1.3	0.000	N	-	[001]
Mo_2PAsTe	4.53	AM	1.3	0.000	N	-	[001]
Tc_2FClO	4.29	AM	4.0	0.357	N	-	[001]
Tc_2FClS	4.43	AM	3.8	0.776	N	-	[001]
Tc_2FClSe	4.97	AFM Stripe	4.0	0.494	N	-	[001]
Tc_2FClTe	4.49	AM	3.7	0.000	N	-	[001]
Tc_2FBrO	4.29	AM	4.0	0.384	N	-	[001]
Tc_2FBrS	4.45	AM	3.8	0.782	N	-	[001]
Tc_2FBrSe	4.49	AM	3.8	0.556	N	-	[001]
Tc_2FBrTe	4.83	AM	3.9	0.355	N	-	[001]
Tc_2FIO	4.29	AM	3.9	0.428	N	-	[001]
Tc_2FIS	4.14	FM	0.7	0.000	N	-	[001]

Tc_2FISe	4.51	AM	3.8	0.509	N	-	[001]
Tc_2FITe	4.23	AFM Zigzag-Y	1.4	0.000	N	-	[001]
Tc_2ClBrO	4.07	AFM Zigzag-Y	2.7	0.000	Y	M-X	[001]
Tc_2ClBrS	4.21	FM	0.6	0.000	N	-	[001]
$Tc_2ClBrSe$	4.78	AM	3.9	0.705	N	-	[001]
$Tc_2ClBrTe$	4.86	AM	3.8	0.316	N	-	[001]
Tc_2ClIO	4.08	AFM Zigzag-Y	2.6	0.000	N	-	[001]
Tc_2ClIS	4.26	FM	0.6	0.000	N	-	[001]
Tc_2ClISe	4.34	FM	0.7	0.000	N	-	[001]
Tc_2ClITe	4.45	AFM Zigzag-X	1.3	0.000	N	-	[001]
Tc_2BrIO	4.25	AM	3.5	0.000	Y	Γ-X, Γ-Y	[001]
Tc_2BrIS	4.28	FM	0.6	0.000	N	-	[001]
Tc_2BrISe	4.83	AM	3.9	0.565	N	-	[001]
Tc_2BrITe	4.50	AFM Zigzag-X	1.3	0.000	N	-	[110]
Tc_2OSO	3.74	AFM Zigzag-X	0.4	0.000	N	-	[100]
Tc_2OSS	3.87	NM	0.0	0.000	N	-	-
Tc_2OSSe	3.91	NM	0.0	0.000	N	-	-
Tc_2OSTe	4.44	AM	1.6	0.000	Y	M-X, M-Y	[001]
Tc_2OSeO	3.77	AFM Zigzag-X	0.5	0.000	N	-	[100]
Tc_2OSeS	3.91	NM	0.0	0.000	N	-	-
Tc_2OSeSe	3.95	NM	0.0	0.000	N	-	-
Tc_2OSeTe	3.99	FM	0.8	0.000	N	-	[001]
Tc_2OTeO	3.81	AFM Zigzag-X	0.4	0.000	N	-	[001]
Tc_2OTeS	3.96	NM	0.0	0.000	N	-	-
Tc_2OTeSe	3.99	NM	0.0	0.000	N	-	-
Tc_2OTeTe	4.03	FM	1.1	0.000	N	-	[100]
Tc_2SSeO	4.02	FM	1.1	0.000	N	-	[100]
Tc_2SSeS	4.07	NM	0.0	0.000	N	-	-
Tc_2SSeSe	4.15	NM	0.0	0.000	N	-	-
Tc_2SSeTe	4.25	NM	0.0	0.000	N	-	-
Tc_2STeO	4.03	FM	1.1	0.000	N	-	[100]
Tc_2STeS	4.11	NM	0.0	0.000	N	-	-
Tc_2STeSe	4.17	NM	0.0	0.000	N	-	-
Tc_2STeTe	4.25	NM	0.0	0.000	N	-	-
Tc_2SeTeO	4.06	FM	1.2	0.000	N	-	[100]
Tc_2SeTeS	4.16	NM	0.0	0.000	N	-	-
$Tc_2SeTeSe$	4.24	NM	0.0	0.000	N	-	-
$Tc_2SeTeTe$	4.34	NM	0.0	0.000	N	-	-
Tc_2NPO	3.74	FM	0.1	0.000	N	-	[001]
Tc_2NPS	4.00	AFM Zigzag-X	0.1	0.000	N	-	[001]
Tc_2NPSe	4.03	NM	0.0	0.000	N	-	-
Tc_2NPTe	4.16	NM	0.0	0.240	N	-	-
Tc_2NAsO	3.76	FM	0.2	0.000	N	-	[001]
Tc_2NAsS	3.98	NM	0.0	0.000	N	-	-
Tc_2NAsSe	4.03	FM	0.7	0.000	N	-	[100]

Tc_2NAsTe	4.16	NM	0.0	0.000	N	-	-
Tc_2PAsO	4.00	AM	1.5	0.000	N	-	[001]
Tc_2PAsS	4.13	FM	0.1	0.000	N	-	[001]
Tc_2PAsSe	4.22	FM	0.1	0.000	N	-	[001]
Tc_2PAsTe	4.32	FM	0.1	0.000	N	-	[100]
Ru_2FClO	4.18	FM	2.8	0.000	N	-	[110]
Ru_2FClS	4.04	AFM Stripe	1.8	0.000	N	-	[001]
Ru_2FClSe	4.10	AFM Stripe	2.1	0.000	N	-	[001]
Ru_2FClTe	4.13	AM	1.8	0.000	Y	M-X, M-Y	[100]
Ru_2FBrO	4.18	FM	2.7	0.000	N	-	[110]
Ru_2FBrS	4.06	AFM Stripe	1.7	0.000	N	-	[001]
Ru_2FBrSe	4.12	AFM Stripe	2.0	0.000	N	-	[001]
Ru_2FBrTe	4.17	AFM Stripe	1.9	0.000	N	-	[001]
Ru_2FIO	4.18	FM	2.6	0.000	N	-	[110]
Ru_2FIS	4.13	AFM Stripe	0.6	0.000	N	-	[100]
Ru_2FISe	4.19	AFM Stripe	1.7	0.000	N	-	[110]
Ru_2FITe	4.23	AM	1.6	0.000	Y	M-X, M-Y	[100]
Ru_2ClBrO	4.10	FM	2.5	0.000	N	-	[100]
Ru_2ClBrS	4.53	AFM Stripe	2.1	0.000	Y	M-X	[100]
$Ru_2ClBrSe$	4.78	AFM Zigzag-Y	2.3	0.000	N	-	[001]
$Ru_2ClBrTe$	3.93	FM	1.8	0.000	N	-	[001]
Ru_2ClIO	4.19	FM	2.6	0.000	N	-	[110]
Ru_2ClIS	4.54	AFM Zigzag-Y	2.1	0.000	N	-	[001]
Ru_2ClISe	4.77	AFM Stripe	2.5	0.000	Y	Γ-X	[001]
Ru_2ClITe	4.15	NM	0.0	0.000	N	-	-
Ru_2BrIO	4.13	FM	2.4	0.000	N	-	[100]
Ru_2BrIS	4.55	AFM Stripe	2.0	0.000	Y	M-X	[100]
Ru_2BrISe	4.84	AFM Zigzag-Y	2.3	0.000	Y	Γ-X	[110]
Ru_2BrITe	4.12	NM	0.0	0.000	N	-	-
Ru_2OSO	3.82	NM	0.0	0.000	N	-	-
Ru_2OSS	3.88	FM	0.2	0.000	N	-	[001]
Ru_2OSSe	3.91	FM	0.1	0.000	N	-	[001]
Ru_2OSTe	4.25	NM	0.0	0.108	N	-	-
Ru_2OSeO	3.85	NM	0.0	0.000	N	-	-
Ru_2OSeS	3.93	FM	0.2	0.000	N	-	[001]
Ru_2OSeSe	3.96	FM	0.2	0.000	N	-	[001]
Ru_2OSeTe	4.00	NM	0.0	0.000	N	-	-
Ru_2OTeO	3.88	NM	0.0	0.000	N	-	-
Ru_2OTeS	3.99	FM	0.2	0.000	N	-	[001]
Ru_2OTeSe	4.01	FM	0.3	0.000	N	-	[001]
Ru_2OTeTe	4.05	NM	0.0	0.000	N	-	-
Ru_2SSeO	4.02	FM	1.1	0.000	N	-	[001]
Ru_2SSeS	4.06	FM	0.4	0.000	N	-	[001]
Ru_2SSeSe	4.13	FM	0.4	0.000	N	-	[100]
Ru_2SSeTe	4.19	FM	0.4	0.000	N	-	[001]

Ru_2STeO	4.04	FM	1.1	0.000	N	-	[110]
Ru_2STeS	4.10	FM	0.5	0.000	N	-	[001]
Ru_2STeSe	4.17	FM	0.5	0.000	N	-	[100]
Ru_2STeTe	4.24	FM	0.4	0.000	N	-	[100]
Ru_2SeTeO	4.06	AFM Zigzag-Y	0.3	0.000	N	-	[100]
Ru_2SeTeS	4.14	FM	0.5	0.000	N	-	[100]
$Ru_2SeTeSe$	4.23	FM	0.5	0.000	N	-	[100]
$Ru_2SeTeTe$	4.32	FM	0.4	0.000	N	-	[100]
Ru_2NPO	4.08	FM	1.0	0.000	N	-	[001]
Ru_2NPS	3.81	NM	0.0	0.000	N	-	-
Ru_2NPSe	4.07	NM	0.0	0.000	N	-	-
Ru_2NPTe	4.10	NM	0.0	0.000	N	-	-
Ru_2NAsO	3.81	NM	0.0	0.000	N	-	-
Ru_2NAsS	4.14	FM	0.6	0.000	N	-	[001]
Ru_2NAsSe	4.20	FM	0.6	0.000	N	-	[100]
Ru_2NAsTe	4.06	NM	0.0	0.000	N	-	-
Ru_2PAsO	4.02	AM	0.4	0.000	N	-	[001]
Ru_2PAsS	4.01	NM	0.0	0.000	N	-	-
Ru_2PAsSe	4.11	NM	0.0	0.000	N	-	-
Ru_2PAsTe	4.22	NM	0.0	0.000	N	-	-
Rh_2FClO	4.04	AFM Stripe	1.1	0.000	N	-	[110]
Rh_2FClS	4.15	AM	0.6	0.000	N	-	[100]
Rh_2FClSe	4.14	AM	0.4	0.000	N	-	[100]
Rh_2FClTe	3.86	NM	0.0	0.000	N	-	-
Rh_2FBrO	4.03	AFM Stripe	1.1	0.000	N	-	[110]
Rh_2FBrS	4.19	AM	0.5	0.000	N	-	[001]
Rh_2FBrSe	4.18	AFM Zigzag-X	0.2	0.000	N	-	[100]
Rh_2FBrTe	3.92	NM	0.0	0.000	N	-	-
Rh_2FIO	4.05	AFM Stripe	1.0	0.000	N	-	[100]
Rh_2FIS	4.26	NM	0.0	0.000	N	-	-
Rh_2FISe	4.26	NM	0.0	0.000	N	-	-
Rh_2FITe	4.08	NM	0.0	0.000	N	-	-
Rh_2ClBrO	4.11	AM	1.1	0.000	N	-	[110]
Rh_2ClBrS	4.37	AFM Stripe	0.9	0.000	N	-	[110]
$Rh_2ClBrSe$	4.44	AM	0.7	0.000	N	-	[100]
$Rh_2ClBrTe$	3.91	NM	0.0	0.000	N	-	-
Rh_2ClIO	4.12	AM	0.8	0.000	N	-	[110]
Rh_2ClIS	4.39	NM	0.0	0.000	N	-	-
Rh_2ClISe	4.43	NM	0.0	0.000	N	-	-
Rh_2ClITe	3.97	NM	0.0	0.000	N	-	-
Rh_2BrIO	4.17	NM	0.0	0.000	N	-	-
Rh_2BrIS	4.42	NM	0.0	0.000	N	-	-
Rh_2BrISe	4.48	FM	0.1	0.000	N	-	[001]
Rh_2BrITe	3.98	NM	0.0	0.000	N	-	-
Rh_2OSO	3.93	FM	0.4	0.000	N	-	[001]

Rh_2OSS	4.43	NM	0.0	0.000	N	-	-
Rh_2OSSe	4.44	NM	0.0	0.000	N	-	-
Rh_2OSTe	3.93	NM	0.0	0.000	N	-	-
Rh_2OSeO	3.96	FM	0.3	0.000	N	-	[001]
Rh_2OSeS	4.05	NM	0.0	0.000	N	-	-
Rh_2OSeSe	4.03	NM	0.0	0.000	N	-	-
Rh_2OSeTe	4.03	NM	0.0	0.000	N	-	-
Rh_2OTeO	4.00	FM	0.3	0.000	N	-	[001]
Rh_2OTeS	4.13	NM	0.0	0.000	N	-	-
Rh_2OTeSe	4.12	NM	0.0	0.000	N	-	-
Rh_2OTeTe	4.11	NM	0.0	0.000	N	-	-
Rh_2SSeO	4.07	FM	0.3	0.000	N	-	[001]
Rh_2SSeS	4.43	NM	0.0	0.000	N	-	-
Rh_2SSeSe	4.28	NM	0.0	0.000	N	-	-
Rh_2SSeTe	4.29	NM	0.0	0.000	N	-	-
Rh_2STeO	4.11	FM	0.4	0.000	N	-	[001]
Rh_2STeS	4.27	NM	0.0	0.000	N	-	-
Rh_2STeSe	4.34	NM	0.0	0.000	N	-	-
Rh_2STeTe	4.36	NM	0.0	0.000	N	-	-
Rh_2SeTeO	4.14	FM	0.4	0.000	N	-	[001]
Rh_2SeTeS	4.50	NM	0.0	0.000	N	-	-
$Rh_2SeTeSe$	4.39	NM	0.0	0.000	N	-	-
$Rh_2SeTeTe$	4.44	NM	0.0	0.000	N	-	-
Rh_2NPO	4.16	NM	0.0	0.000	N	-	-
Rh_2NPS	4.23	NM	0.0	0.000	N	-	-
Rh_2NPSe	4.23	NM	0.0	0.000	N	-	-
Rh_2NPTe	4.26	NM	0.0	0.000	N	-	-
Rh_2NAsO	4.13	NM	0.0	0.000	N	-	-
Rh_2NAsS	4.22	NM	0.0	0.000	N	-	-
Rh_2NAsSe	3.98	FM	0.5	0.000	N	-	[100]
Rh_2NAsTe	4.25	NM	0.0	0.000	N	-	-
Rh_2PAsO	4.19	NM	0.0	0.000	N	-	-
Rh_2PAsS	4.32	NM	0.0	0.000	N	-	-
Rh_2PAsSe	4.35	NM	0.0	0.000	N	-	-
Rh_2PAsTe	4.16	NM	0.0	0.000	N	-	-
Pd_2FClO	4.08	AFM Zigzag-Y	0.7	0.000	N	-	[100]
Pd_2FClS	4.25	NM	0.0	0.000	N	-	-
Pd_2FClSe	4.32	NM	0.0	0.000	N	-	-
Pd_2FClTe	3.78	NM	0.0	0.000	N	-	-
Pd_2FBrO	4.10	AM	0.7	0.000	Y	M-X, M-Y	[001]
Pd_2FBrS	4.27	NM	0.0	0.000	N	-	-
Pd_2FBrSe	4.37	NM	0.0	0.000	N	-	-
Pd_2FBrTe	3.80	NM	0.0	0.000	N	-	-
Pd_2FIO	4.12	AM	0.6	0.000	Y	M-X, M-Y	[001]
Pd_2FIS	4.32	NM	0.0	0.000	N	-	-

Pd_2FISe	4.44	NM	0.0	0.000	N	-	-
Pd_2FITe	3.78	NM	0.0	0.000	N	-	-
Pd_2ClBrO	4.12	AFM Zigzag-Y	0.7	0.000	N	-	[001]
Pd_2ClBrS	4.36	NM	0.0	0.000	N	-	-
$Pd_2ClBrSe$	4.48	NM	0.0	0.000	N	-	-
$Pd_2ClBrTe$	3.87	NM	0.0	0.000	N	-	-
Pd_2ClIO	4.13	FM	0.3	0.000	N	-	[001]
Pd_2ClIS	4.42	NM	0.0	0.000	N	-	-
Pd_2ClISe	4.52	NM	0.0	0.000	N	-	-
Pd_2ClITe	3.92	NM	0.0	0.000	N	-	-
Pd_2BrIO	4.12	FM	0.2	0.000	N	-	[001]
Pd_2BrIS	4.45	FM	0.1	0.000	N	-	[001]
Pd_2BrISe	4.55	NM	0.0	0.000	N	-	-
Pd_2BrITe	3.95	NM	0.0	0.000	N	-	-
Pd_2OSO	4.00	NM	0.0	0.000	N	-	-
Pd_2OSS	4.65	NM	0.0	0.284	N	-	-
Pd_2OSSe	4.64	NM	0.0	0.000	N	-	-
Pd_2OSTe	4.73	NM	0.0	0.000	N	-	-
Pd_2OSeO	4.03	NM	0.0	0.000	N	-	-
Pd_2OSeS	4.67	NM	0.0	0.244	N	-	-
Pd_2OSeSe	4.80	NM	0.0	0.481	N	-	-
Pd_2OSeTe	4.88	NM	0.0	0.000	N	-	-
Pd_2OTeO	4.08	NM	0.0	0.000	N	-	-
Pd_2OTeS	4.72	NM	0.0	0.228	N	-	-
Pd_2OTeSe	4.85	NM	0.0	0.506	N	-	-
Pd_2OTeTe	5.02	NM	0.0	0.552	N	-	-
Pd_2SSeO	4.11	NM	0.0	0.000	N	-	-
Pd_2SSeS	4.76	NM	0.0	0.000	N	-	-
Pd_2SSeSe	4.90	NM	0.0	0.000	N	-	-
Pd_2SSeTe	4.58	NM	0.0	0.000	N	-	-
Pd_2STeO	4.15	NM	0.0	0.000	N	-	-
Pd_2STeS	4.69	NM	0.0	0.000	N	-	-
Pd_2STeSe	4.86	NM	0.0	0.000	N	-	-
Pd_2STeTe	5.06	NM	0.0	0.314	N	-	-
Pd_2SeTeO	4.19	NM	0.0	0.000	N	-	-
Pd_2SeTeS	4.45	NM	0.0	0.000	N	-	-
$Pd_2SeTeSe$	4.85	NM	0.0	0.000	N	-	-
$Pd_2SeTeTe$	5.04	NM	0.0	0.000	N	-	-
Pd_2NPO	4.30	AFM Stripe	0.5	0.000	N	-	[001]
Pd_2NPS	4.64	FM	0.4	0.000	N	-	[001]
Pd_2NPSe	4.76	NM	0.0	0.000	N	-	-
Pd_2NPTe	4.14	NM	0.0	0.000	N	-	-
Pd_2NAsO	4.27	AFM Zigzag-X	0.4	0.000	N	-	[001]
Pd_2NAsS	4.61	NM	0.0	0.000	N	-	-
Pd_2NAsSe	4.75	NM	0.0	0.000	N	-	-

Pd_2NAsTe	4.12	NM	0.0	0.000	N	-	-
Pd_2PAsO	4.36	NM	0.0	0.000	N	-	-
Pd_2PAsS	4.73	NM	0.0	0.000	N	-	-
Pd_2PAsSe	4.46	NM	0.0	0.000	N	-	-
Pd_2PAsTe	4.46	NM	0.0	0.000	N	-	-
Ag_2FClO	4.22	NM	0.0	0.000	N	-	-
Ag_2FClS	4.58	NM	0.0	0.000	N	-	-
Ag_2FClSe	4.61	FM	0.8	0.000	N	-	[001]
Ag_2FClTe	3.97	NM	0.0	0.000	N	-	-
Ag_2FBrO	4.24	NM	0.0	0.000	N	-	-
Ag_2FBrS	4.61	NM	0.0	0.000	N	-	-
Ag_2FBrSe	4.70	NM	0.0	0.000	N	-	-
Ag_2FBrTe	3.99	NM	0.0	0.000	N	-	-
Ag_2FIO	4.28	NM	0.0	0.000	N	-	-
Ag_2FIS	4.67	NM	0.0	0.000	N	-	-
Ag_2FISe	4.75	NM	0.0	0.000	N	-	-
Ag_2FITe	4.05	NM	0.0	0.000	N	-	-
Ag_2ClBrO	4.28	NM	0.0	0.000	N	-	-
Ag_2ClBrS	4.72	NM	0.0	0.000	N	-	-
$Ag_2ClBrSe$	4.83	NM	0.0	0.000	N	-	-
$Ag_2ClBrTe$	4.32	NM	0.0	0.000	N	-	-
Ag_2ClIO	4.32	NM	0.0	0.000	N	-	-
Ag_2ClIS	4.76	NM	0.0	0.000	N	-	-
Ag_2ClISe	4.88	NM	0.0	0.000	N	-	-
Ag_2ClITe	4.36	NM	0.0	0.000	N	-	-
Ag_2BrIO	4.33	NM	0.0	0.000	N	-	-
Ag_2BrIS	4.78	NM	0.0	0.000	N	-	-
Ag_2BrISe	4.88	NM	0.0	0.000	N	-	-
Ag_2BrITe	4.47	NM	0.0	0.000	N	-	-
Ag_2OSO	4.61	NM	0.0	0.000	N	-	-
Ag_2OSS	4.97	NM	0.0	0.000	N	-	-
Ag_2OSSe	5.07	NM	0.0	0.000	N	-	-
Ag_2OSTe	5.27	NM	0.0	0.000	N	-	-
Ag_2OSeO	4.62	NM	0.0	0.000	N	-	-
Ag_2OSeS	4.98	FM	0.1	0.000	N	-	[100]
Ag_2OSeSe	5.08	FM	0.1	0.000	N	-	[100]
Ag_2OSeTe	5.30	NM	0.0	0.000	N	-	-
Ag_2OTeO	4.64	NM	0.0	0.000	N	-	-
Ag_2OTeS	5.00	NM	0.0	0.000	N	-	-
Ag_2OTeSe	5.06	NM	0.0	0.000	N	-	-
Ag_2OTeTe	5.20	NM	0.0	0.000	N	-	-
Ag_2SSeO	4.69	NM	0.0	0.000	N	-	-
Ag_2SSeS	5.07	NM	0.0	0.000	N	-	-
Ag_2SSeSe	5.21	NM	0.0	0.000	N	-	-
Ag_2SSeTe	5.22	NM	0.0	0.000	N	-	-

Ag_2STeO	4.71	NM	0.0	0.000	N	-	-
Ag_2STeS	5.11	NM	0.0	0.000	N	-	-
Ag_2STeSe	5.23	NM	0.0	0.000	N	-	-
Ag_2STeTe	5.43	NM	0.0	0.000	N	-	-
Ag_2SeTeO	4.72	NM	0.0	0.000	N	-	-
Ag_2SeTeS	5.15	NM	0.0	0.000	N	-	-
$Ag_2SeTeSe$	5.27	NM	0.0	0.000	N	-	-
$Ag_2SeTeTe$	4.16	NM	0.0	0.000	N	-	-
Ag_2NPO	4.20	NM	0.0	0.841	N	-	-
Ag_2NPS	5.02	NM	0.0	0.666	N	-	-
Ag_2NPSe	4.63	NM	0.0	1.159	N	-	-
Ag_2NPTe	4.79	NM	0.0	1.057	N	-	-
Ag_2NAsO	4.22	NM	0.0	0.267	N	-	-
Ag_2NAsS	4.98	NM	0.0	0.567	N	-	-
Ag_2NAsSe	5.01	NM	0.0	0.580	N	-	-
Ag_2NAsTe	5.07	NM	0.0	0.488	N	-	-
Ag_2PAsO	4.70	NM	0.0	0.000	N	-	-
Ag_2PAsS	5.12	NM	0.0	0.645	N	-	-
Ag_2PAsSe	4.67	NM	0.0	0.612	N	-	-
Ag_2PAsTe	4.60	NM	0.0	0.156	N	-	-
Cd_2FClO	4.38	NM	0.0	1.915	N	-	-
Cd_2FClS	4.63	NM	0.0	2.827	N	-	-
Cd_2FClSe	4.83	NM	0.0	1.468	N	-	-
Cd_2FClTe	4.88	NM	0.0	0.911	N	-	-
Cd_2FBrO	4.40	NM	0.0	1.899	N	-	-
Cd_2FBrS	4.65	NM	0.0	2.767	N	-	-
Cd_2FBrSe	4.89	NM	0.0	1.125	N	-	-
Cd_2FBrTe	4.89	NM	0.0	0.618	N	-	-
Cd_2FIO	4.42	NM	0.0	1.841	N	-	-
Cd_2FIS	4.68	NM	0.0	2.419	N	-	-
Cd_2FISe	4.96	NM	0.0	0.643	N	-	-
Cd_2FITe	4.85	NM	0.0	0.298	N	-	-
Cd_2ClBrO	4.47	NM	0.0	2.165	N	-	-
Cd_2ClBrS	4.88	NM	0.0	1.445	N	-	-
$Cd_2ClBrSe$	5.00	NM	0.0	0.777	N	-	-
$Cd_2ClBrTe$	5.12	NM	0.0	0.134	N	-	-
Cd_2ClIO	4.50	NM	0.0	2.254	N	-	-
Cd_2ClIS	4.90	NM	0.0	1.308	N	-	-
Cd_2ClISe	5.08	NM	0.0	0.417	N	-	-
Cd_2ClITe	5.19	NM	0.0	0.000	N	-	-
Cd_2BrIO	4.52	NM	0.0	2.276	N	-	-
Cd_2BrIS	4.96	NM	0.0	0.973	N	-	-
Cd_2BrISe	5.10	NM	0.0	0.330	N	-	-
Cd_2BrITe	5.23	NM	0.0	0.000	N	-	-
Cd_2OSO	4.60	NM	0.0	0.000	N	-	-

Cd_2OSS	4.83	NM	0.0	0.839	N	-	-
Cd_2OSSe	4.89	NM	0.0	0.720	N	-	-
Cd_2OSTe	5.05	NM	0.0	0.000	N	-	-
Cd_2OSeO	4.61	NM	0.0	0.000	N	-	-
Cd_2OSeS	4.83	NM	0.0	0.676	N	-	-
Cd_2OSeSe	4.90	NM	0.0	0.754	N	-	-
Cd_2OSeTe	4.96	NM	0.0	0.489	N	-	-
Cd_2OTeO	4.61	NM	0.0	0.000	N	-	-
Cd_2OTeS	4.85	NM	0.0	0.116	N	-	-
Cd_2OTeSe	4.88	NM	0.0	0.290	N	-	-
Cd_2OTeTe	4.97	NM	0.0	0.169	N	-	-
Cd_2SSeO	4.80	NM	0.0	0.578	N	-	-
Cd_2SSeS	5.17	NM	0.0	0.000	N	-	-
Cd_2SSeSe	5.34	NM	0.0	0.000	N	-	-
Cd_2SSeTe	5.42	NM	0.0	0.000	N	-	-
Cd_2STeO	4.60	NM	0.0	0.000	N	-	-
Cd_2STeS	5.21	NM	0.0	0.000	N	-	-
Cd_2STeSe	5.31	NM	0.0	0.000	N	-	-
Cd_2STeTe	5.44	NM	0.0	0.000	N	-	-
Cd_2SeTeO	4.57	NM	0.0	0.000	N	-	-
Cd_2SeTeS	5.28	NM	0.0	0.000	N	-	-
$Cd_2SeTeSe$	5.37	NM	0.0	0.000	N	-	-
$Cd_2SeTeTe$	5.51	NM	0.0	0.000	N	-	-
Cd_2NPO	4.63	NM	0.0	0.000	N	-	-
Cd_2NPS	5.01	NM	0.0	0.000	N	-	-
Cd_2NPSe	5.07	NM	0.0	0.000	N	-	-
Cd_2NPTe	5.31	NM	0.0	0.000	N	-	-
Cd_2NAsO	4.33	NM	0.0	0.000	N	-	-
Cd_2NAsS	4.27	NM	0.0	0.000	N	-	-
Cd_2NAsSe	5.03	NM	0.0	0.000	N	-	-
Cd_2NAsTe	4.33	NM	0.0	0.000	N	-	-
Cd_2PAsO	4.78	NM	0.0	0.000	N	-	-
Cd_2PAsS	5.22	NM	0.0	0.000	N	-	-
Cd_2PAsSe	5.35	NM	0.0	0.000	N	-	-
Cd_2PAsTe	5.54	NM	0.0	0.000	N	-	-

TABLE S4: Summary of key properties for all 300 candidates from the structural framework Janus M_2AA'.

Material	Lattice constant (Å)	Magnetic order	Moment (μ_B)	Band gap (eV)	Dirac-cone	K-location	MA
Sc_2FCl	4.17	FM	0.6	0.000	N	-	[001]
Sc_2FBr	4.21	FM	0.7	0.000	N	-	[100]
Sc_2FI	4.26	FM	0.7	0.000	N	-	[100]
Sc_2ClBr	4.36	FM	0.7	0.000	N	-	[100]
Sc_2ClI	4.41	FM	0.7	0.000	N	-	[100]
Sc_2BrI	4.45	FM	0.7	0.000	N	-	[100]
Sc_2OS	4.03	NM	0.0	0.000	N	-	-

Sc_2OSe	4.07	NM	0.0	0.000	N	-	-
Sc_2OTe	4.13	NM	0.0	0.000	N	-	-
Sc_2SSe	4.32	NM	0.0	0.000	N	-	-
Sc_2STe	4.40	NM	0.0	0.000	N	-	-
Sc_2SeTe	4.45	FM	0.2	0.000	N	-	[001]
Sc_2NP	4.36	NM	0.0	0.000	N	-	-
Sc_2NAs	4.37	NM	0.0	0.000	N	-	-
Sc_2PAs	4.77	NM	0.0	0.000	N	-	-
Ti_2FCl	4.13	AFM Zigzag-Y	1.6	0.259	N	-	[001]
Ti_2FBr	4.17	AM	1.6	0.314	N	-	[001]
Ti_2FI	4.21	AFM Zigzag-Y	1.6	0.557	N	-	[001]
Ti_2ClBr	4.25	AM	1.5	0.535	N	-	[001]
Ti_2ClI	4.29	AM	1.5	0.224	N	-	[001]
Ti_2BrI	4.33	AM	1.5	0.536	N	-	[001]
Ti_2OS	3.77	AM	0.6	0.000	N	-	[001]
Ti_2OSe	3.80	AM	0.6	0.000	N	-	[001]
Ti_2OTe	3.86	AM	0.7	0.000	N	-	[100]
Ti_2SSe	3.99	AFM Zigzag-Y	1.0	0.000	N	-	[110]
Ti_2STe	4.12	AFM Stripe	1.1	0.000	N	-	[110]
Ti_2SeTe	4.19	AFM Stripe	1.2	0.000	Y	Γ-Y	[110]
Ti_2NP	4.08	AM	0.5	0.000	N	-	[001]
Ti_2NAs	4.04	FM	0.9	0.000	N	-	[100]
Ti_2PAs	4.54	AFM Stripe	1.2	0.000	Y	Γ-Y	[100]
V_2FCl	3.89	AM	2.6	0.000	Y	M-X, M-Y	[001]
V_2FBr	3.93	AM	2.6	0.000	Y	M-X, M-Y	[001]
V_2FI	3.99	AM	2.6	0.156	N	-	[001]
V_2ClBr	4.00	AM	2.6	0.217	N	-	[001]
V_2ClI	4.05	AM	2.6	0.309	N	-	[001]
V_2BrI	4.09	AM	2.7	0.329	N	-	[001]
V_2OS	3.63	AM	1.9	0.000	Y	M-X, M-Y	[001]
V_2OSe	3.67	AM	2.0	0.000	Y	M-X, M-Y	[001]
V_2OTe	3.73	AM	2.2	0.000	Y	M-X, M-Y	[100]
V_2SSe	3.89	AM	2.3	0.000	N	-	[100]
V_2STe	3.97	AM	2.3	0.000	N	-	[100]
V_2SeTe	4.04	AM	2.4	0.000	N	-	[100]
V_2NP	3.97	AFM Stripe	2.1	0.000	N	-	[100]
V_2NAs	3.94	AM	1.8	0.000	Y	M-X, M-Y	[100]
V_2PAs	3.71	AM	1.6	0.000	N	-	[100]
Cr_2FCl	3.94	AM	4.0	0.000	N	-	[001]
Cr_2FBr	3.98	AM	4.0	0.000	N	-	[001]
Cr_2FI	4.05	AM	4.0	0.000	N	-	[110]
Cr_2ClBr	4.07	AM	4.1	0.180	N	-	[001]
Cr_2ClI	4.15	AM	4.1	0.149	N	-	[001]
Cr_2BrI	4.21	AM	4.1	0.159	N	-	[110]
Cr_2OS	3.61	AM	3.3	0.331	N	-	[100]

Cr_2OSe	3.67	AM	3.3	0.144	N	-	[100]
Cr_2OTe	3.74	AM	3.4	0.000	N	-	[001]
Cr_2SSe	3.87	AM	3.5	0.651	N	-	[100]
Cr_2STe	3.97	AM	3.6	0.192	N	-	[001]
Cr_2SeTe	4.04	AM	3.6	0.232	N	-	[001]
Cr_2NP	3.95	AM	3.3	0.000	Y	M-X, M-Y	[100]
Cr_2NAs	3.95	AM	3.4	0.000	Y	M-X, M-Y	[001]
Cr_2PAs	4.30	AFM Zigzag-X	3.7	0.000	N	-	[100]
Mn_2FCl	4.04	AM	4.6	0.694	N	-	[001]
Mn_2FBr	4.07	AM	4.6	0.741	N	-	[001]
Mn_2FI	4.11	AM	4.6	0.768	N	-	[001]
Mn_2ClBr	4.14	AM	4.6	0.531	N	-	[001]
Mn_2ClI	4.18	AM	4.6	0.545	N	-	[100]
Mn_2BrI	4.20	AM	4.6	0.489	N	-	[100]
Mn_2OS	3.73	AM	4.3	0.000	N	-	[100]
Mn_2OSe	3.78	AM	4.3	0.000	N	-	[001]
Mn_2OTe	3.82	AM	4.4	0.000	N	-	[001]
Mn_2SSe	3.99	AM	4.4	0.000	N	-	[001]
Mn_2STe	4.05	AM	4.4	0.000	N	-	[001]
Mn_2SeTe	4.13	AM	4.4	0.000	N	-	[100]
Mn_2NP	4.05	AM	4.2	0.000	N	-	[110]
Mn_2NAs	4.05	FM	4.2	0.000	N	-	[100]
Mn_2PAs	4.40	FM	4.3	0.000	N	-	[100]
Fe_2FCl	3.77	AFM Stripe	3.6	0.000	N	-	[001]
Fe_2FBr	3.80	AFM Stripe	3.6	0.000	N	-	[001]
Fe_2FI	3.86	AFM Zigzag-Y	3.6	0.911	N	-	[001]
Fe_2ClBr	3.81	AFM Zigzag-X	3.2	0.000	N	-	[001]
Fe_2ClI	3.90	AFM Zigzag-X	3.1	0.000	N	-	[001]
Fe_2BrI	3.94	AFM Zigzag-X	3.1	0.000	N	-	[001]
Fe_2OS	3.64	AM	3.6	0.000	N	-	[001]
Fe_2OSe	3.69	AM	3.6	0.000	N	-	[001]
Fe_2OTe	3.76	AM	3.5	0.000	N	-	[001]
Fe_2SSe	3.86	AM	3.5	0.000	N	-	[001]
Fe_2STe	3.94	AM	3.5	0.000	N	-	[001]
Fe_2SeTe	4.01	AM	3.5	0.000	N	-	[001]
Fe_2NP	3.86	AFM Stripe	3.2	0.000	N	-	[001]
Fe_2NAs	3.85	AFM Stripe	3.2	0.000	N	-	[110]
Fe_2PAs	4.08	AFM Stripe	3.2	0.000	N	-	[001]
Co_2FCl	3.69	AFM Zigzag-Y	2.1	0.773	N	-	[100]
Co_2FBr	3.72	AFM Zigzag-Y	2.1	0.819	N	-	[100]
Co_2FI	3.82	AFM Zigzag-Y	2.0	0.794	N	-	[001]
Co_2ClBr	3.79	AFM Zigzag-Y	2.0	0.687	N	-	[100]
Co_2ClI	3.89	AFM Zigzag-Y	2.0	0.533	N	-	[100]
Co_2BrI	3.95	AFM Zigzag-X	2.0	0.632	N	-	[100]
Co_2OS	3.56	AFM Zigzag-Y	2.5	0.103	N	-	[001]

Co_2OSe	3.61	FM	2.4	0.000	N	-	[001]
Co_2OTe	3.63	FM	2.4	0.000	N	-	[100]
Co_2SSe	3.75	FM	2.1	0.000	N	-	[100]
Co_2STe	3.82	FM	2.1	0.000	N	-	[100]
Co_2SeTe	3.90	FM	2.2	0.000	N	-	[001]
Co_2NP	3.65	AFM Zigzag-Y	1.9	0.000	N	-	[100]
Co_2NAs	3.75	AFM Zigzag-X	1.9	0.000	Y	Γ-X	[100]
Co_2PAs	4.07	AFM Zigzag-Y	2.1	0.000	N	-	[001]
Ni_2FCl	3.56	AM	1.0	1.789	N	-	[100]
Ni_2FBr	3.63	AM	1.0	0.000	N	-	[100]
Ni_2FI	3.75	AFM Stripe	1.0	1.633	N	-	[100]
Ni_2ClBr	3.70	AM	1.0	1.659	N	-	[100]
Ni_2ClI	3.82	AM	1.0	1.289	N	-	[001]
Ni_2BrI	3.88	AM	1.0	1.380	N	-	[001]
Ni_2OS	3.36	FM	0.9	0.000	N	-	[001]
Ni_2OSe	3.43	FM	1.1	0.000	N	-	[100]
Ni_2OTe	3.45	FM	1.2	0.000	N	-	[001]
Ni_2SSe	3.57	FM	0.6	0.000	N	-	[001]
Ni_2STe	3.59	FM	0.5	0.000	N	-	[100]
Ni_2SeTe	3.66	FM	0.5	0.000	N	-	[100]
Ni_2NP	3.63	NM	0.0	0.538	N	-	-
Ni_2NAs	3.61	NM	0.0	0.199	N	-	-
Ni_2PAs	3.37	NM	0.0	0.000	N	-	-
Cu_2FCl	3.62	NM	0.0	1.773	N	-	-
Cu_2FBr	3.69	NM	0.0	1.493	N	-	-
Cu_2FI	3.84	NM	0.0	1.041	N	-	-
Cu_2ClBr	3.77	NM	0.0	1.396	N	-	-
Cu_2ClI	3.90	NM	0.0	0.993	N	-	-
Cu_2BrI	3.96	NM	0.0	1.057	N	-	-
Cu_2OS	3.47	NM	0.0	0.000	N	-	-
Cu_2OSe	3.50	NM	0.0	0.000	N	-	-
Cu_2OTe	3.54	NM	0.0	0.000	N	-	-
Cu_2SSe	3.66	NM	0.0	0.000	N	-	-
Cu_2STe	3.72	NM	0.0	0.000	N	-	-
Cu_2SeTe	3.80	NM	0.0	0.000	N	-	-
Cu_2NP	3.74	NM	0.0	0.000	N	-	-
Cu_2NAs	3.70	NM	0.0	0.000	N	-	-
Cu_2PAs	3.98	NM	0.0	0.000	N	-	-
Zn_2FCl	3.69	NM	0.0	1.208	N	-	-
Zn_2FBr	3.73	NM	0.0	1.165	N	-	-
Zn_2FI	3.81	NM	0.0	1.097	N	-	-
Zn_2ClBr	3.76	NM	0.0	1.132	N	-	-
Zn_2ClI	3.84	NM	0.0	0.909	N	-	-
Zn_2BrI	3.86	NM	0.0	0.639	N	-	-
Zn_2OS	3.60	NM	0.0	0.000	N	-	-

Zn_2OSe	3.63	NM	0.0	0.000	N	-	-
Zn_2OTe	3.65	NM	0.0	0.000	N	-	-
Zn_2SSe	3.79	NM	0.0	0.000	N	-	-
Zn_2STe	3.87	NM	0.0	0.000	N	-	-
Zn_2SeTe	3.94	NM	0.0	0.000	N	-	-
Zn_2NP	3.86	NM	0.0	0.000	N	-	-
Zn_2NAs	3.59	NM	0.0	0.000	N	-	-
Zn_2PAs	3.82	NM	0.0	0.000	N	-	-
Y_2FCl	4.68	FM	0.6	0.000	N	-	[100]
Y_2FBr	4.72	FM	0.6	0.000	N	-	[100]
Y_2FI	4.69	FM	0.6	0.000	N	-	[100]
Y_2ClBr	4.88	FM	0.6	0.000	N	-	[001]
Y_2ClI	4.93	FM	0.6	0.000	N	-	[100]
Y_2BrI	4.97	FM	0.6	0.000	N	-	[100]
Y_2OS	4.49	NM	0.0	0.000	N	-	-
Y_2OSe	4.53	NM	0.0	0.000	N	-	-
Y_2OTe	4.58	NM	0.0	0.000	N	-	-
Y_2SSe	4.80	NM	0.0	0.000	N	-	-
Y_2STe	4.88	FM	0.1	0.000	N	-	[001]
Y_2SeTe	4.93	FM	0.1	0.000	N	-	[001]
Y_2NP	4.80	NM	0.0	0.000	N	-	-
Y_2NAs	4.80	NM	0.0	0.000	N	-	-
Y_2PAs	5.23	NM	0.0	0.000	N	-	-
Zr_2FCl	4.22	FM	0.3	0.000	N	-	[001]
Zr_2FBr	4.24	FM	0.3	0.000	N	-	[001]
Zr_2FI	4.24	FM	0.2	0.000	N	-	[001]
Zr_2ClBr	4.23	NM	0.0	0.000	N	-	-
Zr_2ClI	4.24	NM	0.0	0.000	N	-	-
Zr_2BrI	4.24	NM	0.0	0.000	N	-	-
Zr_2OS	4.09	NM	0.0	0.000	N	-	-
Zr_2OSe	4.13	NM	0.0	0.000	N	-	-
Zr_2OTe	4.18	NM	0.0	0.000	N	-	-
Zr_2SSe	4.29	NM	0.0	0.000	N	-	-
Zr_2STe	4.35	NM	0.0	0.000	N	-	-
Zr_2SeTe	4.38	NM	0.0	0.000	N	-	-
Zr_2NP	4.43	NM	0.0	0.000	N	-	-
Zr_2NAs	4.39	FM	0.5	0.000	N	-	[001]
Zr_2PAs	4.47	FM	0.4	0.000	N	-	[001]
Nb_2FCl	4.07	AM	1.9	0.000	Y	M-X, M-Y	[001]
Nb_2FBr	4.08	AM	1.9	0.000	Y	M-X, M-Y	[001]
Nb_2FI	4.10	AM	1.8	0.000	Y	M-X, M-Y	[001]
Nb_2ClBr	4.09	AM	1.8	0.000	Y	M-X, M-Y	[001]
Nb_2ClI	4.11	AM	1.7	0.000	Y	M-X, M-Y	[001]
Nb_2BrI	4.11	AM	1.6	0.000	Y	M-X, M-Y	[001]
Nb_2OS	3.84	NM	0.0	0.000	N	-	-

Nb_2OSe	3.88	NM	0.0	0.000	N	-	-
Nb_2OTe	3.93	NM	0.0	0.000	N	-	-
Nb_2SSe	3.95	FM	0.2	0.000	N	-	[001]
Nb_2STe	3.98	FM	0.2	0.000	N	-	[100]
Nb_2SeTe	3.99	AM	0.1	0.000	N	-	[100]
Nb_2NP	3.88	NM	0.0	0.000	N	-	-
Nb_2NAs	3.87	NM	0.0	0.000	N	-	-
Nb_2PAs	3.97	NM	0.0	0.000	N	-	-
Mo_2FCl	3.98	AM	3.0	0.000	N	-	[001]
Mo_2FBr	3.81	AM	2.7	0.000	Y	M-X, M-Y	[110]
Mo_2FI	3.95	AM	2.9	0.000	N	-	[110]
Mo_2ClBr	3.86	AM	1.1	0.000	N	-	[001]
Mo_2ClI	3.90	AM	1.2	0.000	N	-	[001]
Mo_2BrI	3.93	AM	1.2	0.000	N	-	[001]
Mo_2OS	3.75	AM	1.6	0.000	Y	M-X, M-Y	[001]
Mo_2OSe	3.79	AM	1.6	0.000	Y	M-X, M-Y	[001]
Mo_2OTe	3.83	AM	1.6	0.000	Y	M-X, M-Y	[001]
Mo_2SSe	3.87	AM	1.7	0.000	Y	M-X, M-Y	[001]
Mo_2STe	3.91	AM	1.7	0.000	Y	M-X, M-Y	[001]
Mo_2SeTe	3.93	AM	1.8	0.000	Y	M-X, M-Y	[001]
Mo_2NP	3.71	NM	0.0	0.000	N	-	-
Mo_2NAs	3.73	AM	0.3	0.000	N	-	[100]
Mo_2PAs	3.69	NM	0.0	0.000	N	-	-
Tc_2FCl	3.84	AM	3.0	0.000	N	-	[001]
Tc_2FBr	3.85	AM	3.0	0.000	N	-	[001]
Tc_2FI	3.90	AM	3.0	0.000	N	-	[001]
Tc_2ClBr	3.82	AM	2.7	0.000	N	-	[001]
Tc_2ClI	3.74	AM	2.5	0.000	N	-	[001]
Tc_2BrI	3.76	AM	2.5	0.000	N	-	[001]
Tc_2OS	3.66	AM	1.4	0.000	N	-	[001]
Tc_2OSe	3.70	AM	1.5	0.000	N	-	[001]
Tc_2OTe	3.74	AM	1.6	0.000	N	-	[001]
Tc_2SSe	3.78	AM	1.5	0.000	N	-	[001]
Tc_2STe	3.82	AM	1.5	0.000	N	-	[001]
Tc_2SeTe	3.85	AM	1.5	0.000	N	-	[001]
Tc_2NP	3.65	AM	1.0	0.000	N	-	[001]
Tc_2NAs	3.72	AM	1.1	0.000	N	-	[001]
Tc_2PAs	3.72	AM	1.3	0.000	N	-	[100]
Ru_2FCl	3.85	FM	2.7	0.000	N	-	[001]
Ru_2FBr	3.66	FM	2.6	0.000	N	-	[001]
Ru_2FI	3.74	FM	2.6	0.000	N	-	[100]
Ru_2ClBr	3.71	FM	2.6	0.000	N	-	[001]
Ru_2ClI	3.89	FM	2.5	0.000	N	-	[100]
Ru_2BrI	4.08	FM	2.5	0.000	N	-	[001]
Ru_2OS	3.60	AFM Zigzag-Y	0.6	0.000	N	-	[001]

Ru_2OSe	3.63	AFM Zigzag-Y	0.7	0.000	N	-	[001]
Ru_2OTe	3.64	FM	1.1	0.000	N	-	[001]
Ru_2SSe	3.82	FM	1.1	0.000	N	-	[001]
Ru_2STe	3.85	FM	1.1	0.000	N	-	[001]
Ru_2SeTe	3.90	FM	1.3	0.000	N	-	[001]
Ru_2NP	3.68	AFM Zigzag-Y	0.5	0.000	N	-	[100]
Ru_2NAs	3.71	AFM Zigzag-Y	0.5	0.000	N	-	[100]
Ru_2PAs	3.88	AFM Stripe	1.2	0.000	N	-	[100]
Rh_2FCl	3.75	FM	1.4	0.000	N	-	[001]
Rh_2FBr	3.78	FM	1.4	0.000	N	-	[100]
Rh_2FI	3.96	NM	0.0	0.000	N	-	-
Rh_2ClBr	3.86	AM	0.7	0.000	N	-	[001]
Rh_2ClI	3.97	AM	0.4	0.000	N	-	[100]
Rh_2BrI	3.99	AM	0.3	0.000	N	-	[100]
Rh_2OS	3.66	FM	0.1	0.000	N	-	[001]
Rh_2OSe	3.69	FM	0.1	0.000	N	-	[001]
Rh_2OTe	3.72	NM	0.0	0.000	N	-	-
Rh_2SSe	3.79	NM	0.0	0.000	N	-	-
Rh_2STe	3.82	NM	0.0	0.000	N	-	-
Rh_2SeTe	3.87	NM	0.0	0.000	N	-	-
Rh_2NP	3.89	NM	0.0	0.000	N	-	-
Rh_2NAs	3.81	NM	0.0	0.000	N	-	-
Rh_2PAs	3.97	NM	0.0	0.000	N	-	-
Pd_2FCl	3.95	AFM Stripe	0.9	0.000	N	-	[100]
Pd_2FBr	3.99	AFM Stripe	0.9	0.000	N	-	[110]
Pd_2FI	4.05	AFM Stripe	0.8	1.593	N	-	[110]
Pd_2ClBr	4.03	AM	0.8	1.856	N	-	[001]
Pd_2ClI	4.09	AM	0.7	1.162	N	-	[001]
Pd_2BrI	4.13	AM	0.7	1.036	N	-	[001]
Pd_2OS	3.76	NM	0.0	0.000	N	-	-
Pd_2OSe	3.81	NM	0.0	0.000	N	-	-
Pd_2OTe	3.89	NM	0.0	0.000	N	-	-
Pd_2SSe	3.92	NM	0.0	0.000	N	-	-
Pd_2STe	4.00	FM	0.2	0.000	N	-	[001]
Pd_2SeTe	4.06	FM	0.2	0.000	N	-	[100]
Pd_2NP	4.11	NM	0.0	1.091	N	-	-
Pd_2NAs	4.10	NM	0.0	0.931	N	-	-
Pd_2PAs	3.85	NM	0.0	0.000	N	-	-
Ag_2FCl	4.09	NM	0.0	1.711	N	-	-
Ag_2FBr	4.13	NM	0.0	1.556	N	-	-
Ag_2FI	4.21	NM	0.0	1.274	N	-	-
Ag_2ClBr	4.20	NM	0.0	1.932	N	-	-
Ag_2ClI	4.28	NM	0.0	1.788	N	-	-
Ag_2BrI	4.32	NM	0.0	1.699	N	-	-
Ag_2OS	3.94	NM	0.0	0.000	N	-	-

Ag_2OSe	3.97	NM	0.0	0.000	N	-	-
Ag_2OTe	4.01	NM	0.0	0.000	N	-	-
Ag_2SSe	4.08	NM	0.0	0.000	N	-	-
Ag_2STe	4.13	NM	0.0	0.000	N	-	-
Ag_2SeTe	4.19	NM	0.0	0.000	N	-	-
Ag_2NP	4.44	NM	0.0	0.000	N	-	-
Ag_2NAs	3.86	NM	0.0	0.000	N	-	-
Ag_2PAs	4.44	NM	0.0	0.000	N	-	-
Cd_2FCl	4.17	NM	0.0	0.642	N	-	-
Cd_2FBr	4.18	NM	0.0	0.679	N	-	-
Cd_2FI	4.21	NM	0.0	0.745	N	-	-
Cd_2ClBr	4.21	NM	0.0	0.814	N	-	-
Cd_2ClI	4.24	NM	0.0	0.777	N	-	-
Cd_2BrI	4.25	NM	0.0	0.716	N	-	-
Cd_2OS	4.07	NM	0.0	0.000	N	-	-
Cd_2OSe	4.10	NM	0.0	0.000	N	-	-
Cd_2OTe	4.13	NM	0.0	0.000	N	-	-
Cd_2SSe	4.24	NM	0.0	0.000	N	-	-
Cd_2STe	4.28	NM	0.0	0.000	N	-	-
Cd_2SeTe	4.34	NM	0.0	0.000	N	-	-
Cd_2NP	4.60	NM	0.0	0.000	N	-	-
Cd_2NAs	4.56	NM	0.0	0.000	N	-	-
Cd_2PAs	4.31	NM	0.0	0.000	N		-

5. Band structures from high-throughput calculations

On the basis of ground-state magnetic orderings, we calculate the spin-polarized band structures of all 2600 candidates (from all four design frameworks, M_2A_2B, M_2A_2, $M_2AA'B$, and M_2AA') and classify them into four types: semiconducting, metallic, half-metallic, and Dirac-cone semimetallic. In total, we identify 612 out of 2600 altermagnetic candidates from our high-throughput calculation results, and we selectively demonstrate their band structures in the following figures (Fig. S7-S21).

About 79 out of 612 altermagnets host simultaneously Dirac-cone linear dispersions within the spin-splitting paths, giving rise to CSML combining with ultra-fast transport of massless Dirac Fermions. The Dirac-cone band structures are determined following such criteria: linear dispersions forming Dirac-cone shapes with crossing point within about ± 0.5 eV from Fermi level (accessible by doping), situating between clear conduction and valence bands, and better not intersected by other bands from the same spin channel. The near-Fermi-level crossing points of Dirac cones are marked by black circles to give clear demonstration.

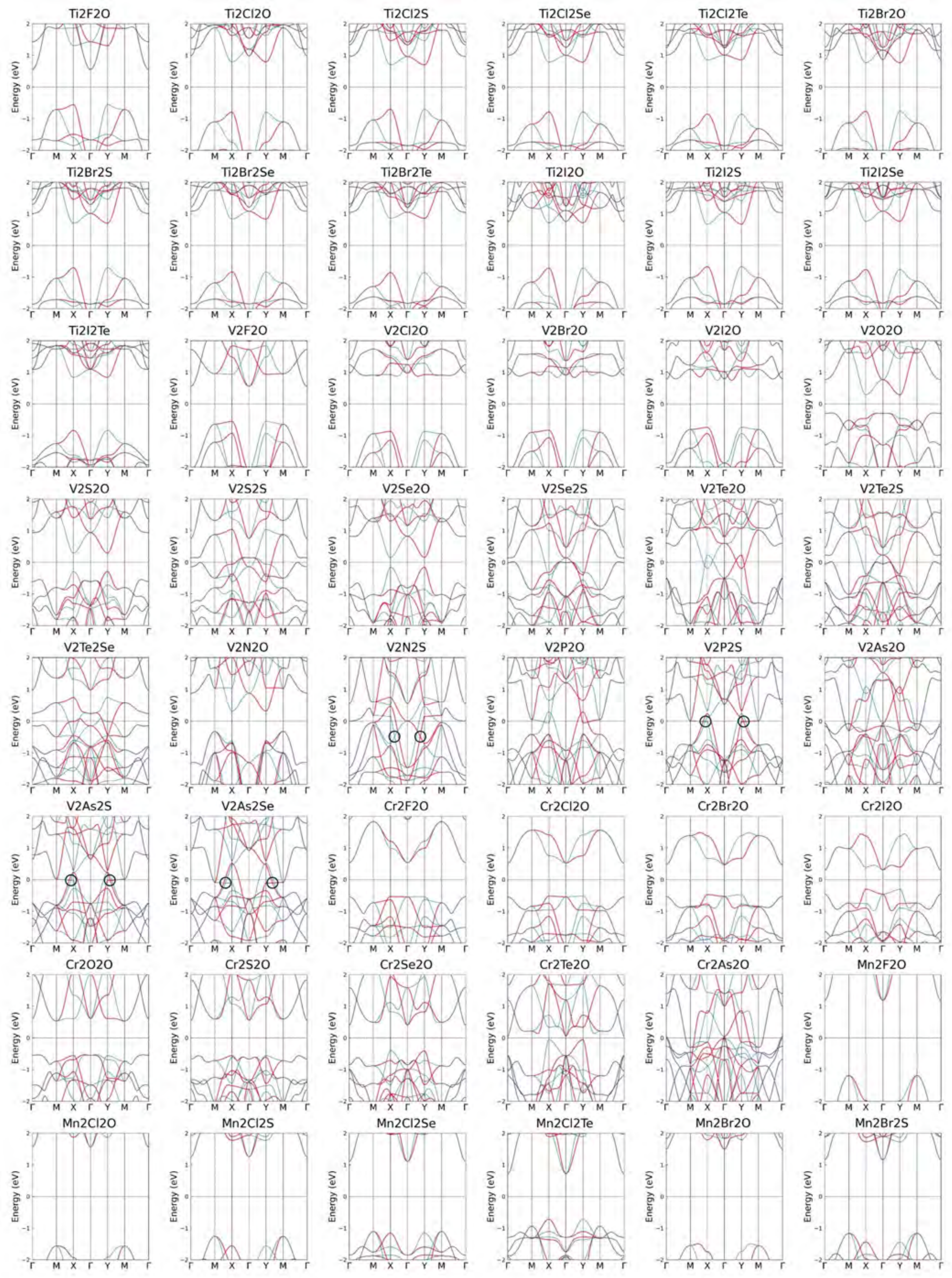

FIG. S7. Band structures of altermagnetic candidates of M_2A_2B framework from Ti_2F_2O to Mn_2Br_2S.

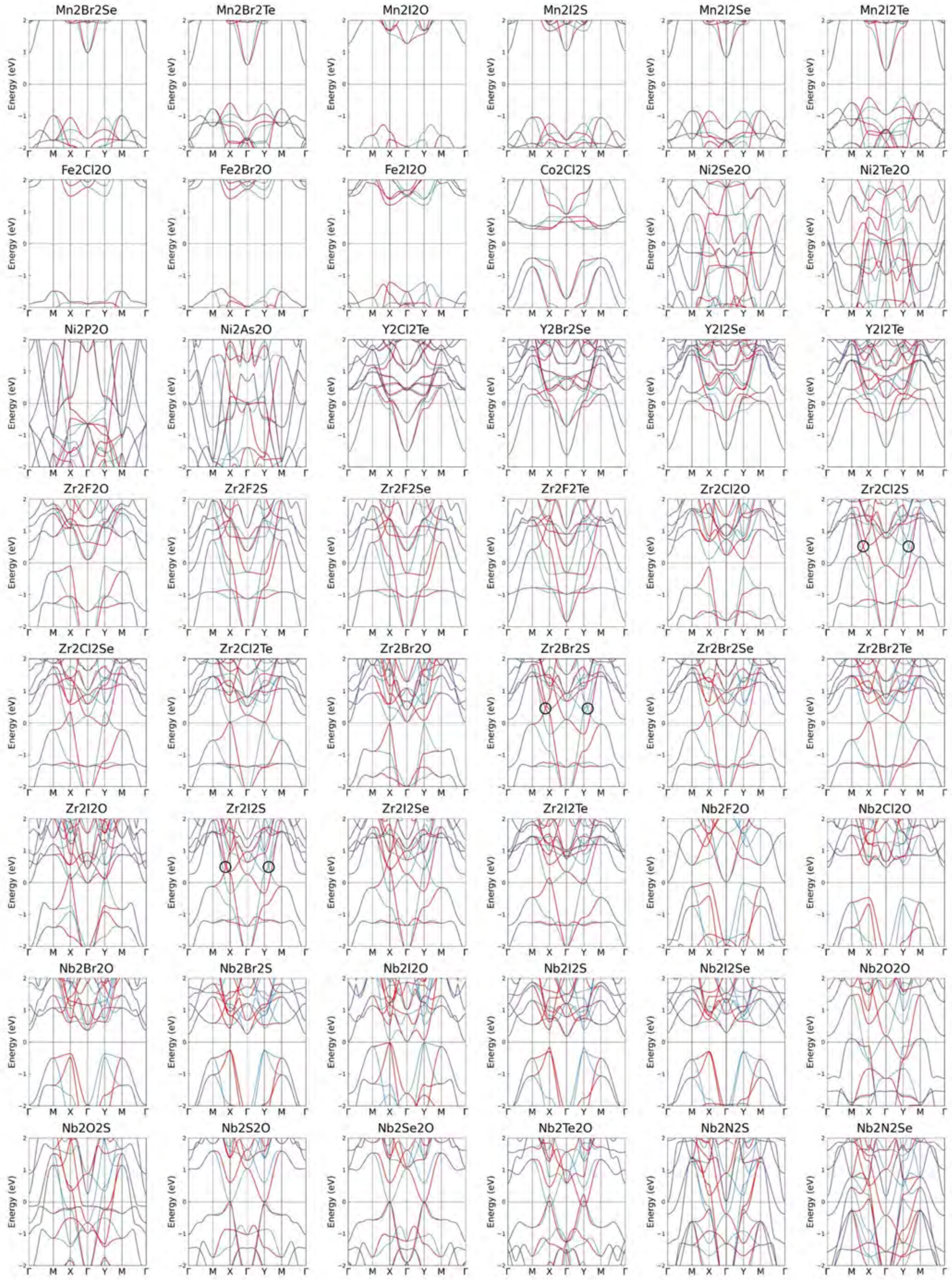

FIG. S8. Band structures of altermagnetic candidates of M_2A_2B framework from Mn_2Br_2Se to Nb_2N_2Se.

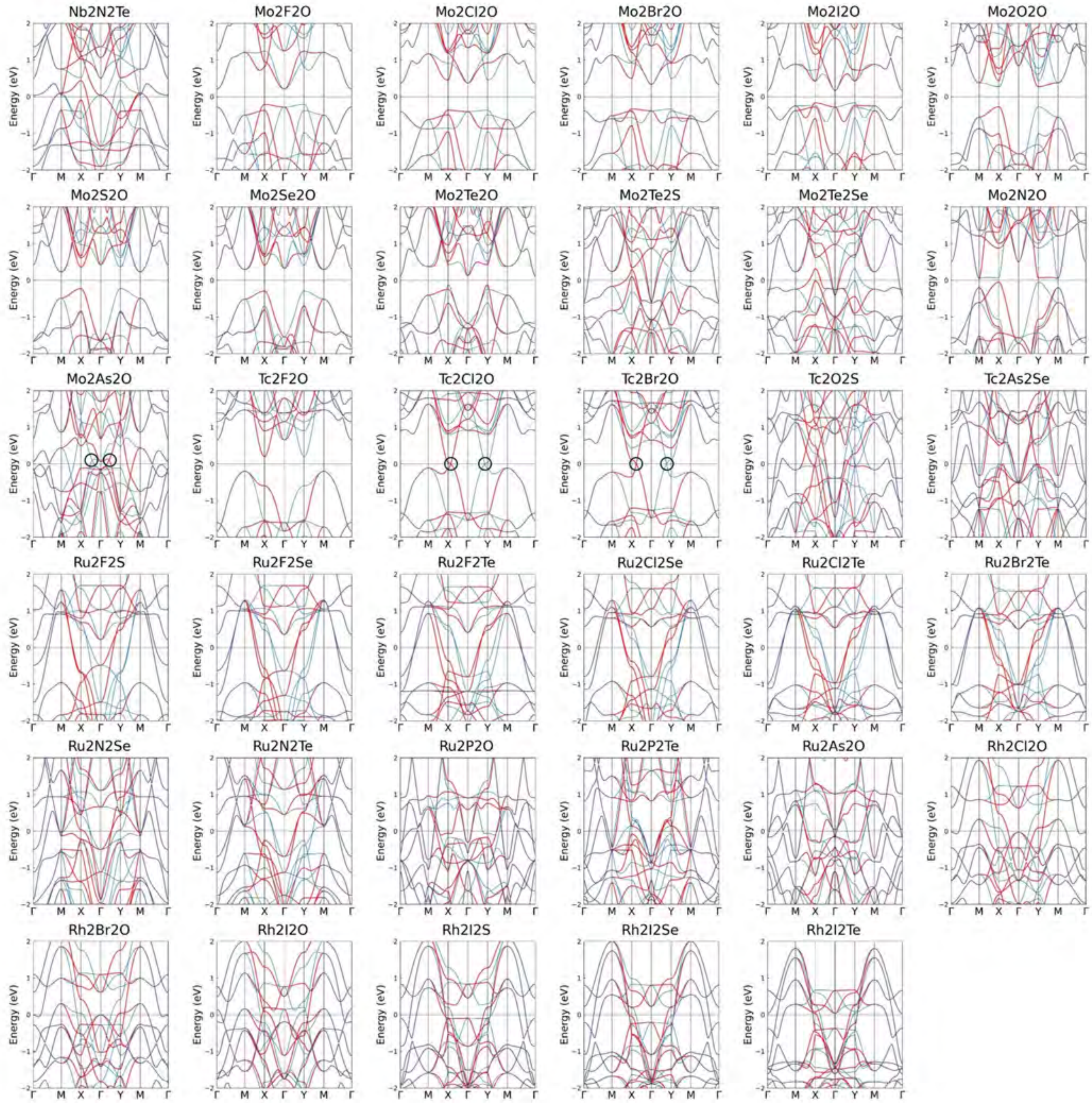

FIG. S9. Band structures of altermagnetic candidates of M_2A_2B framework from Nb_2N_2Te to Rh_2I_2Te.

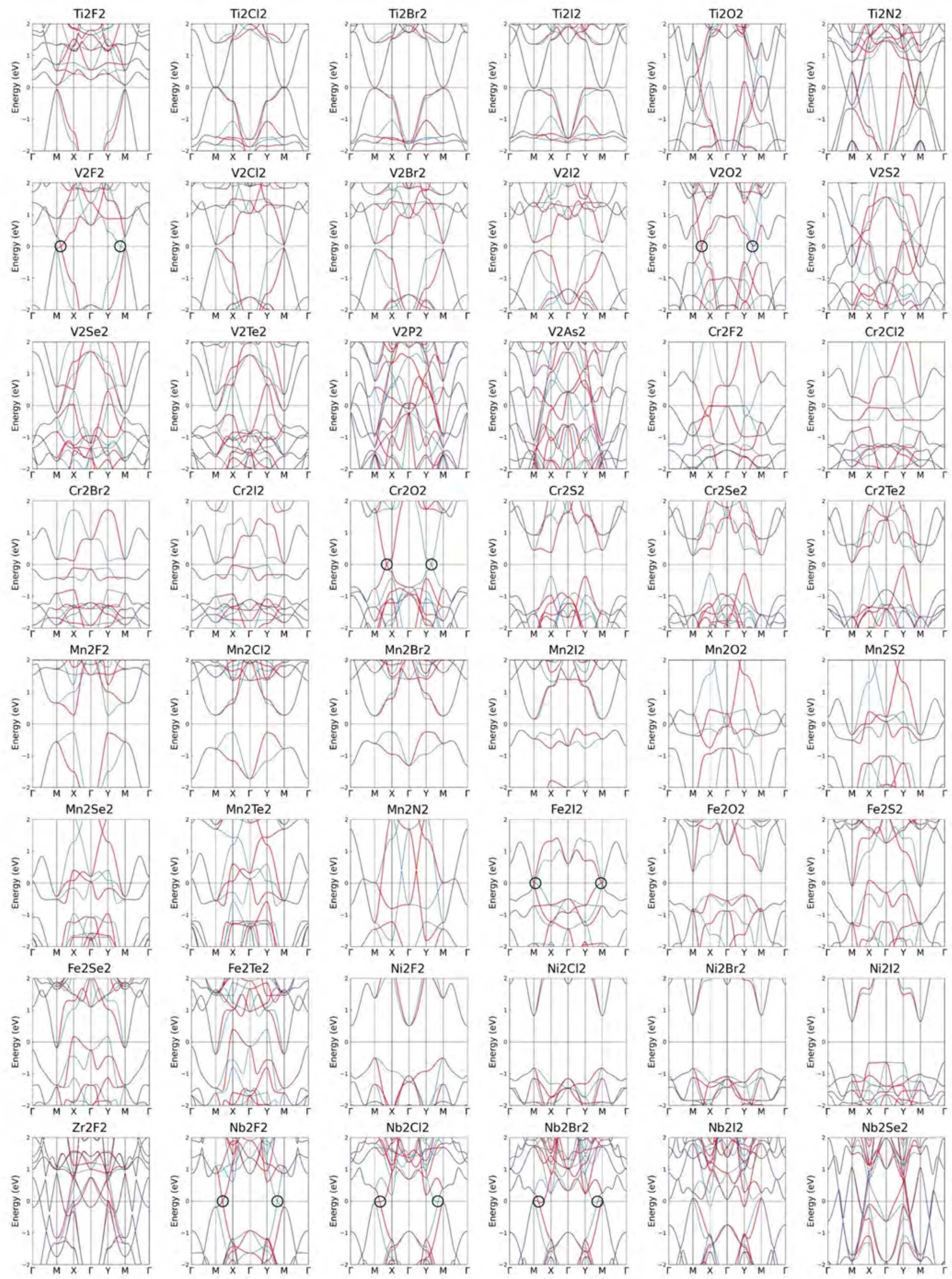

FIG. S10. Band structures of altermagnetic candidates of M_2A_2 framework from Ti_2F_2 to Nb_2Se_2.

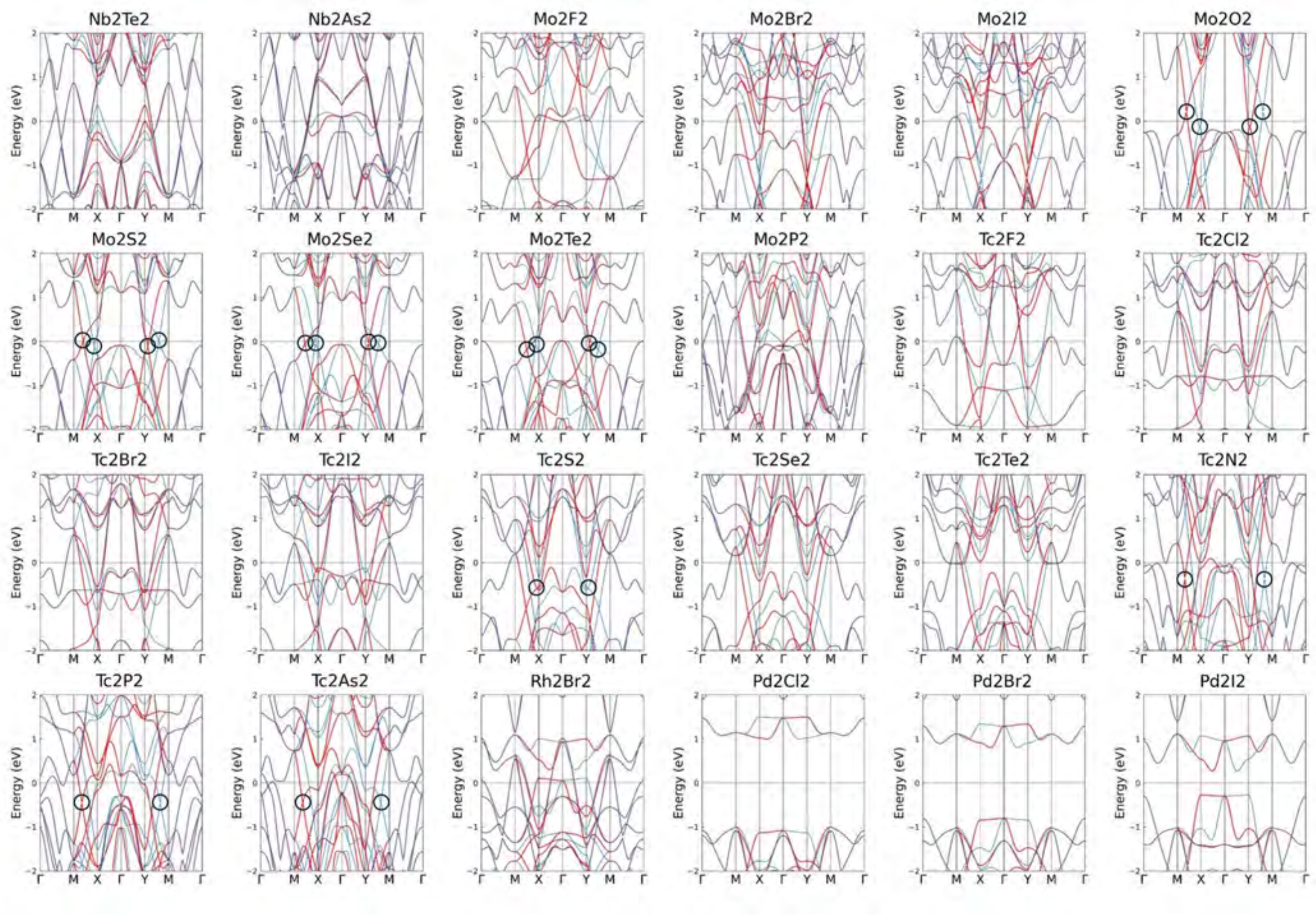

FIG. S11. Band structures of altermagnetic candidates of M_2A_2 framework from Nb_2Te_2 to Pd_2I_2.

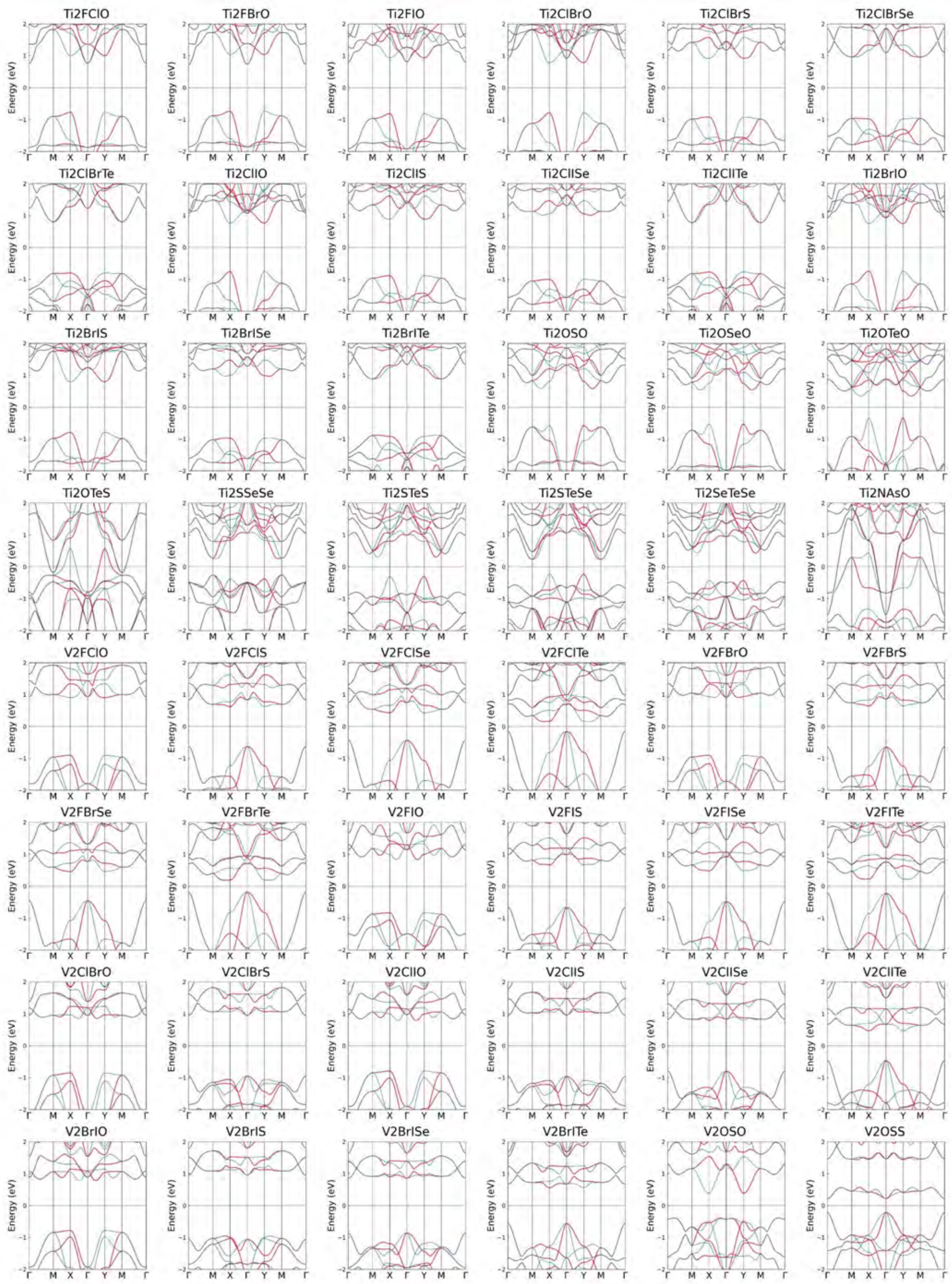

FIG. S12. Band structures of altermagnetic candidates of Janus $M_2AA'B$ framework from Ti_2FClO to V_2OSS.

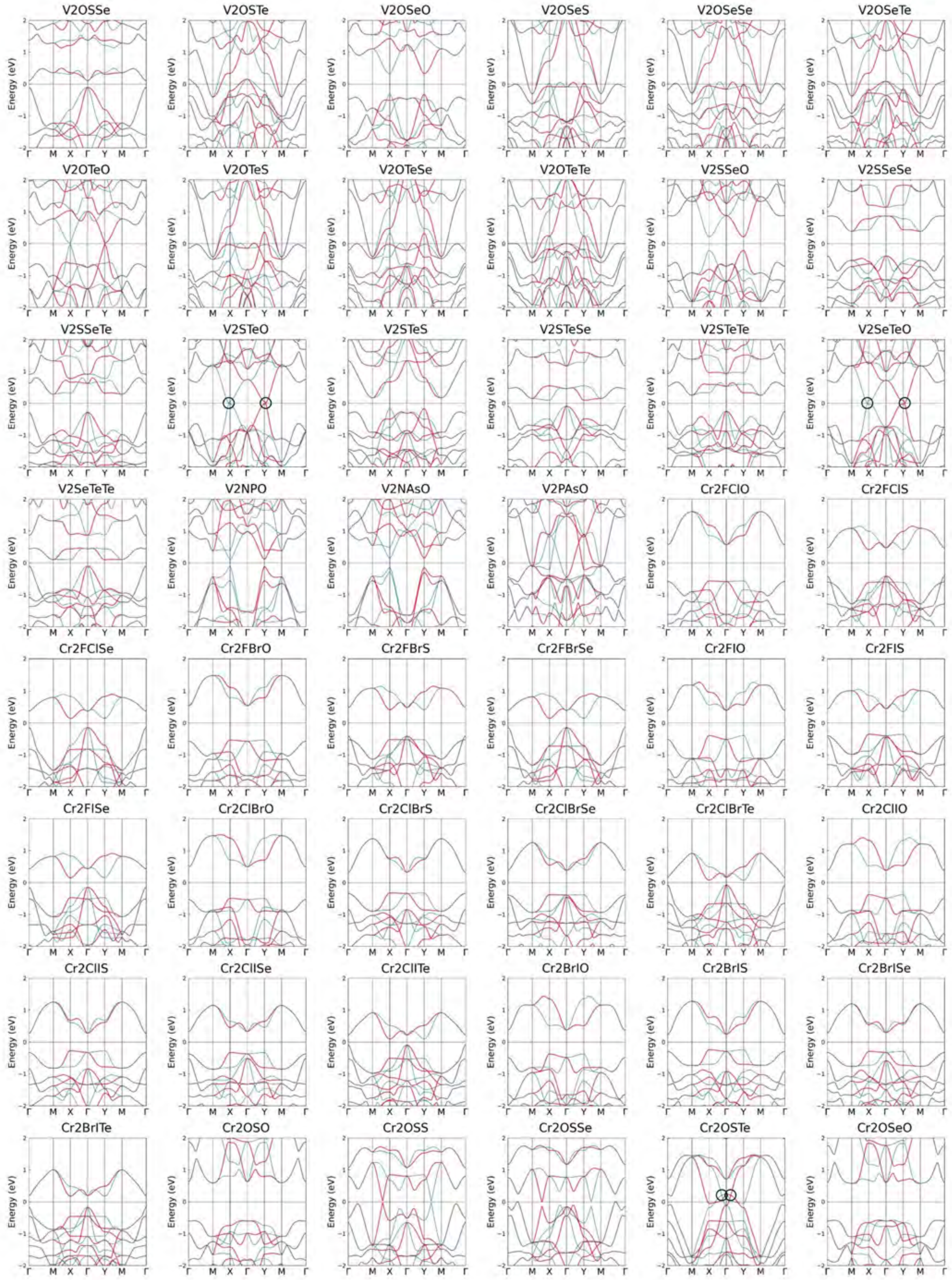

FIG. S13. Band structures of altermagnetic candidates of Janus $M_2AA'B$ framework from V_2OSSe to Cr_2OSeO.

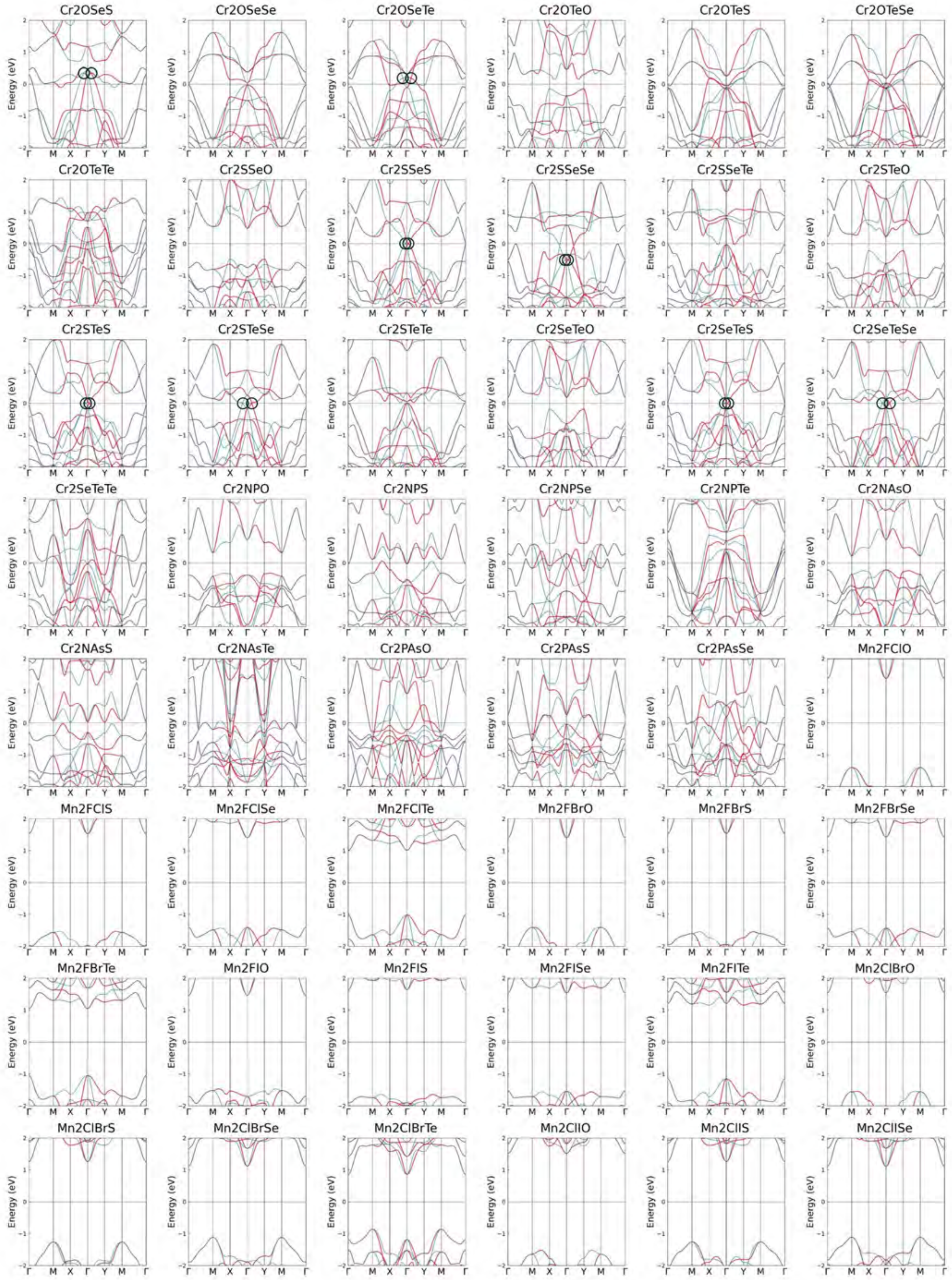

FIG. S14. Band structures of altermagnetic candidates of Janus $M_2AA'B$ framework from Cr_2OSeS to Mn_2ClISe.

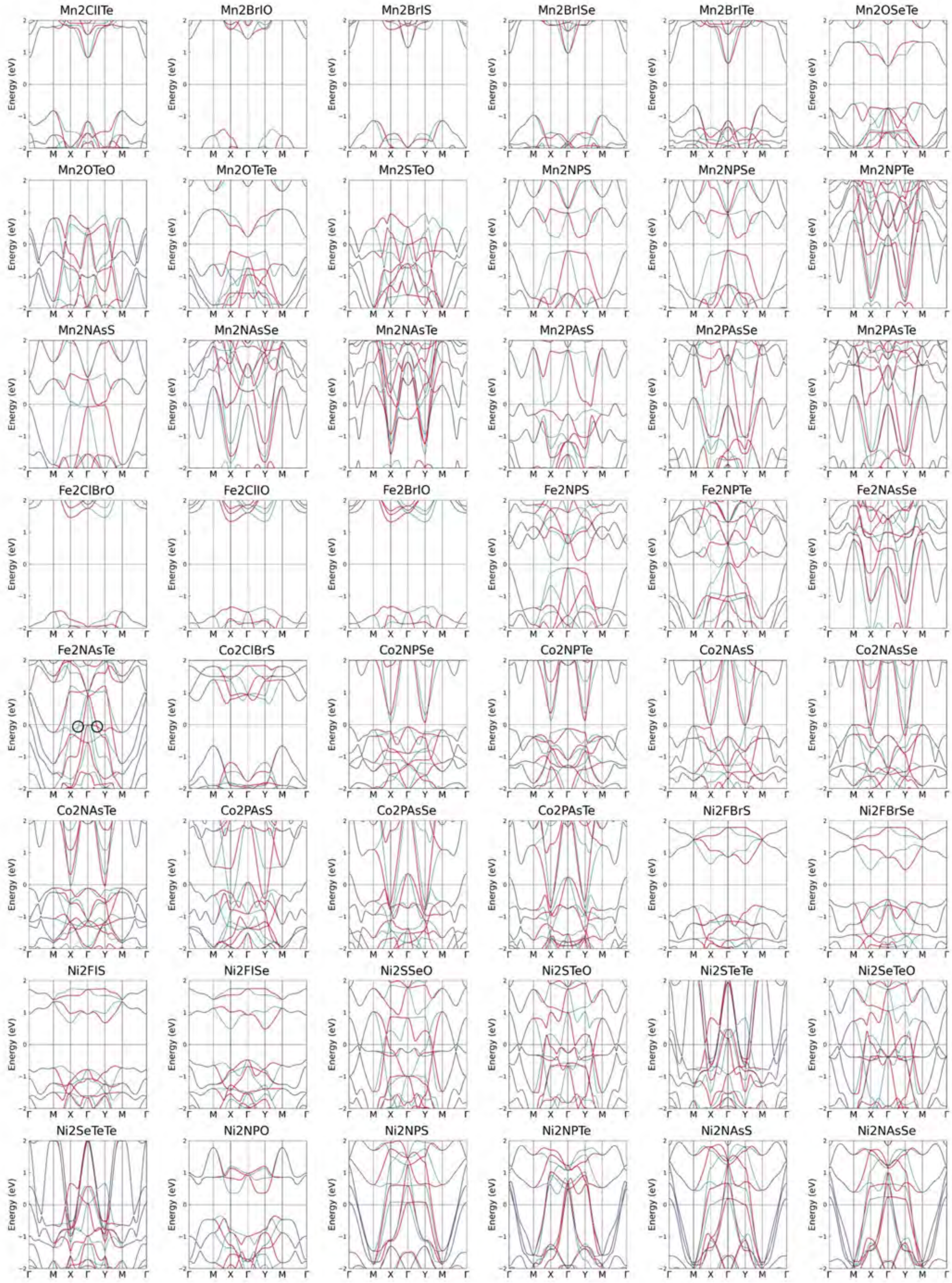

FIG. S15. Band structures of altermagnetic candidates of Janus $M_2AA'B$ framework from Mn_2ClITe to Ni_2NAsSe.

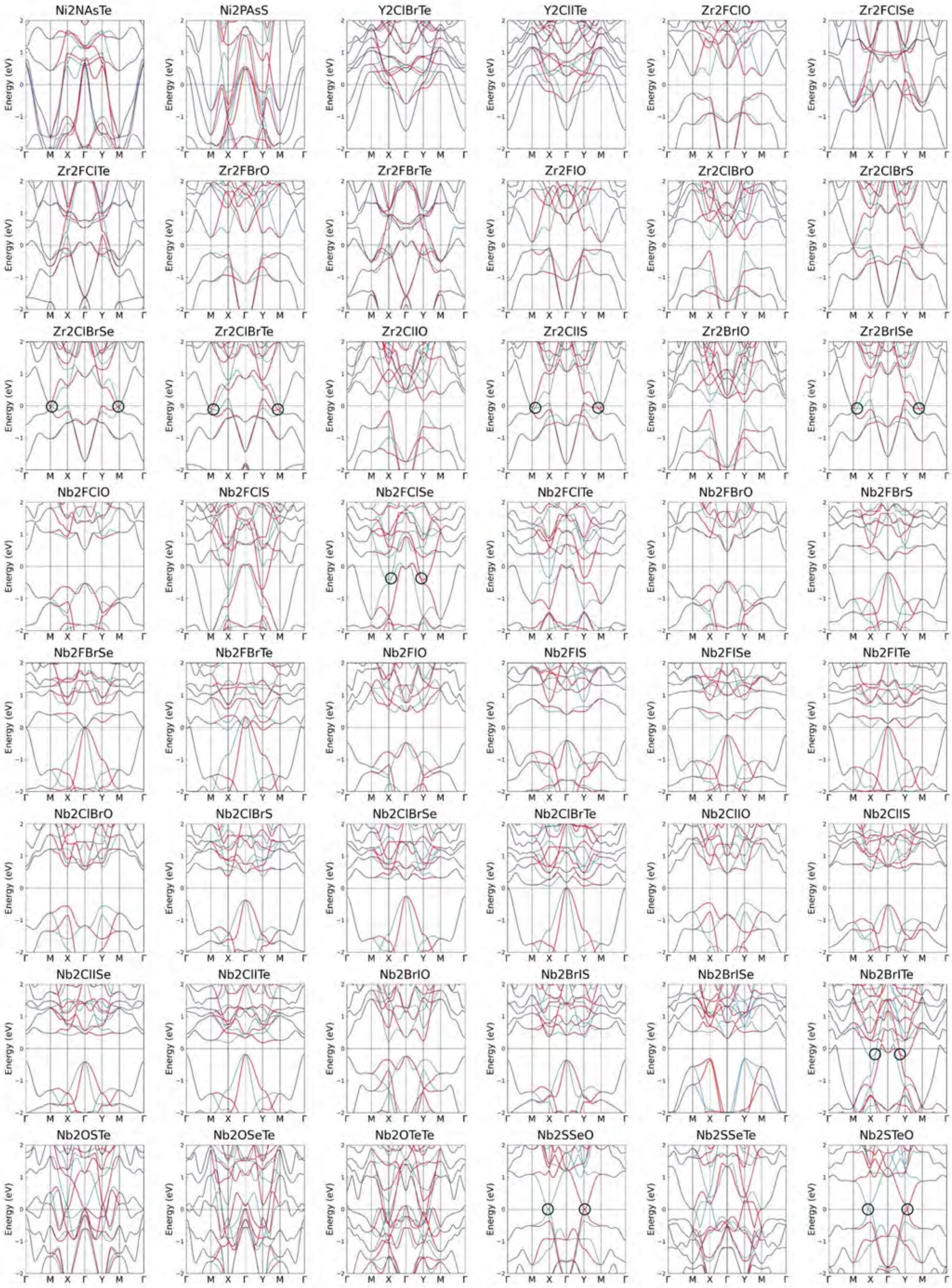

FIG. S16. Band structures of altermagnetic candidates of Janus $M_2AA'B$ framework from Ni_2NAsTe to Nb_2STeO.

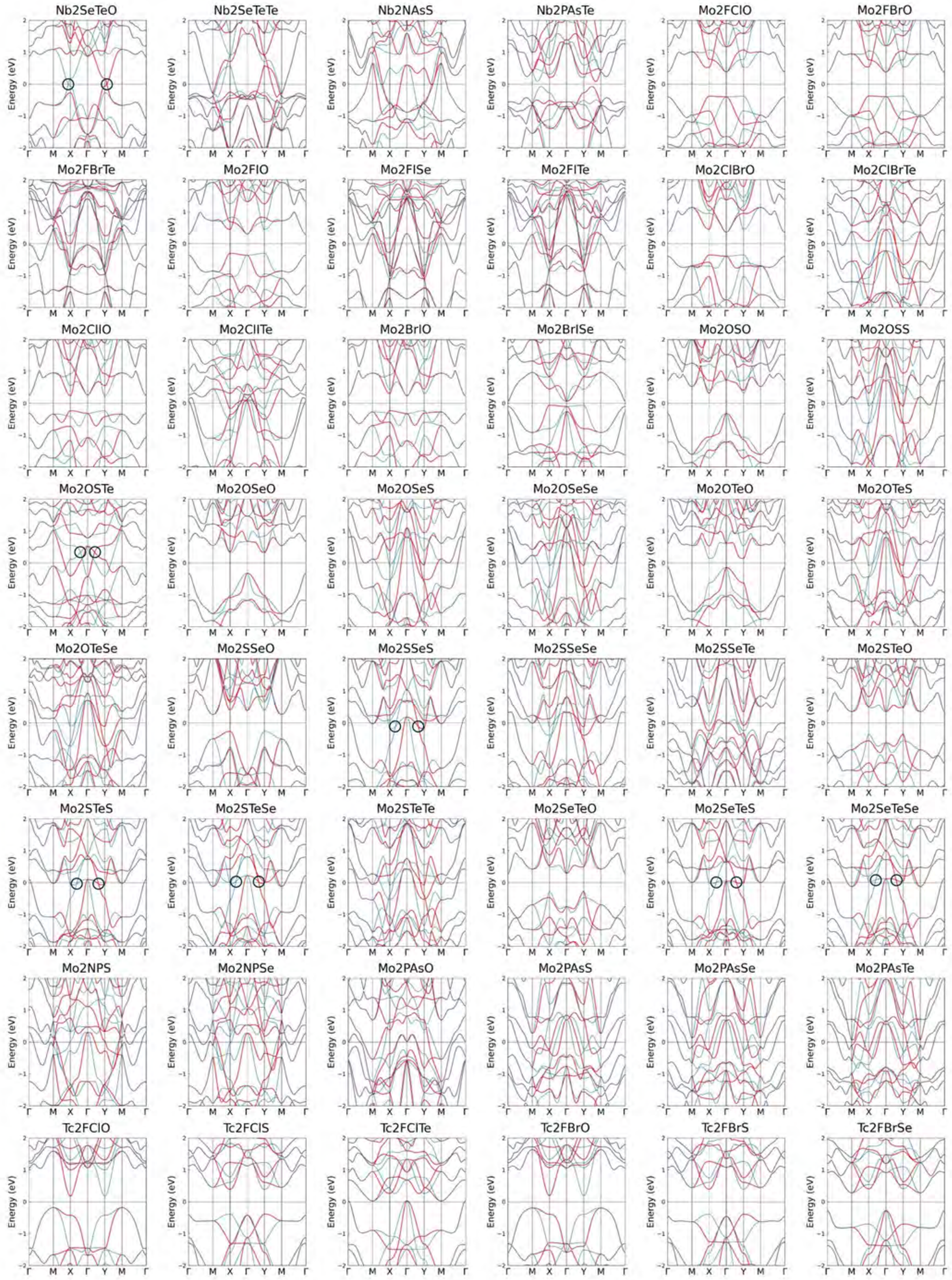

FIG. S17. Band structures of altermagnetic candidates of Janus $M_2AA'B$ framework from Nb_2SeTeO to Tc_2FBrSe.

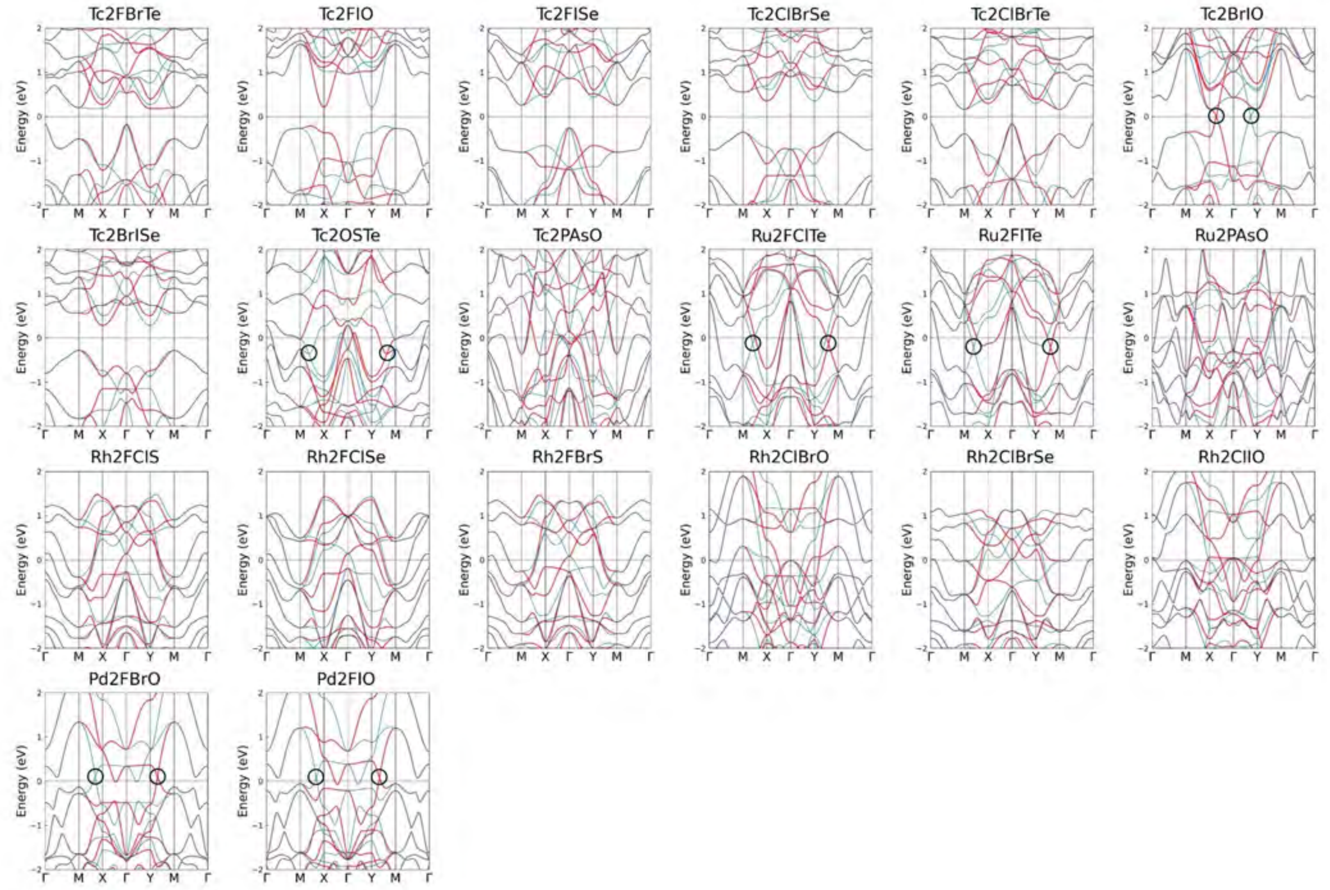

FIG. S18. Band structures of altermagnetic candidates of Janus $M_2AA'B$ framework from Tc_2FBrTe to Pd_2FIO.

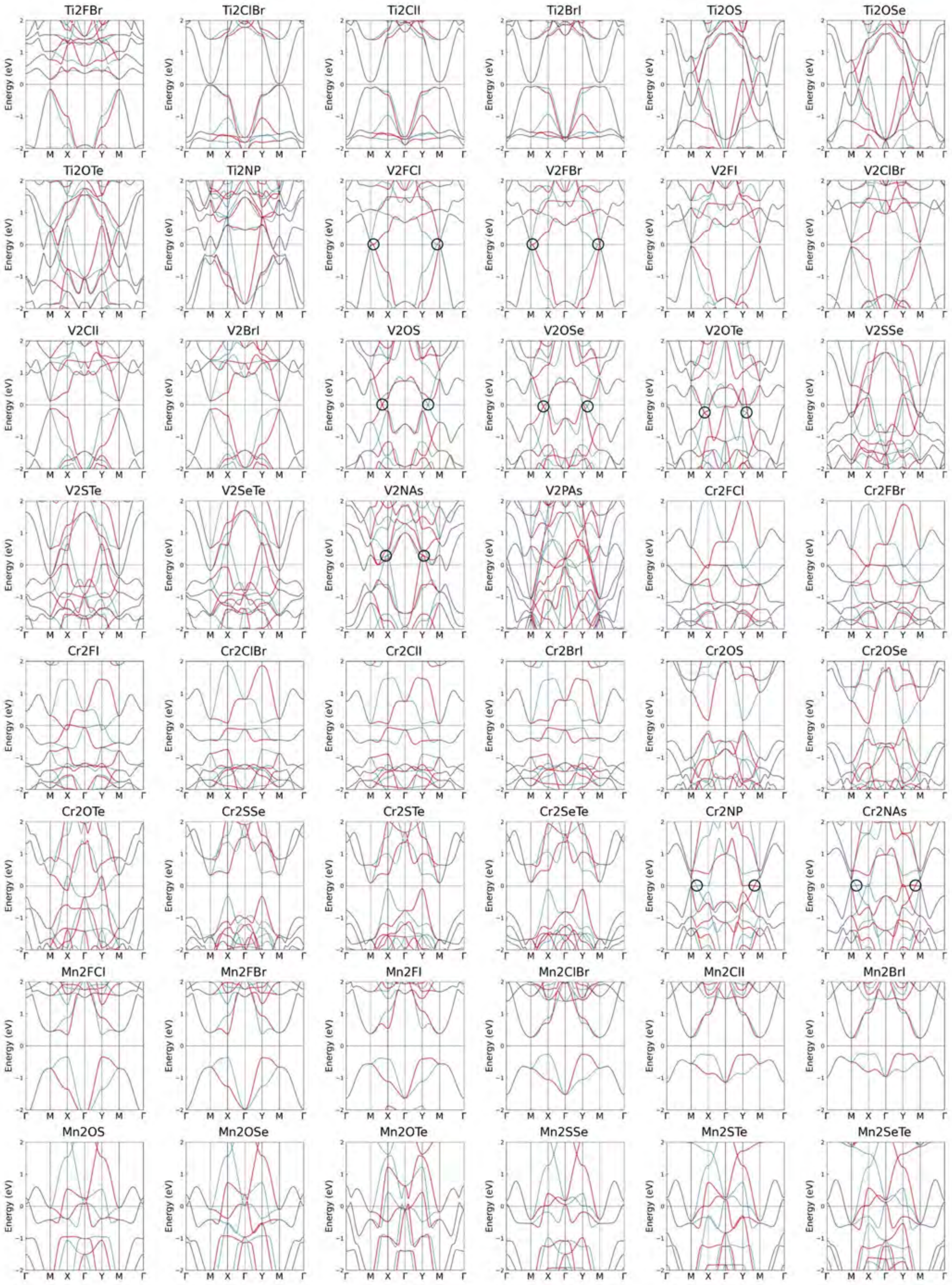

FIG. S19. Band structures of altermagnetic candidates of Janus M_2AA' framework from Ti_2FBr to Mn_2SeTe.

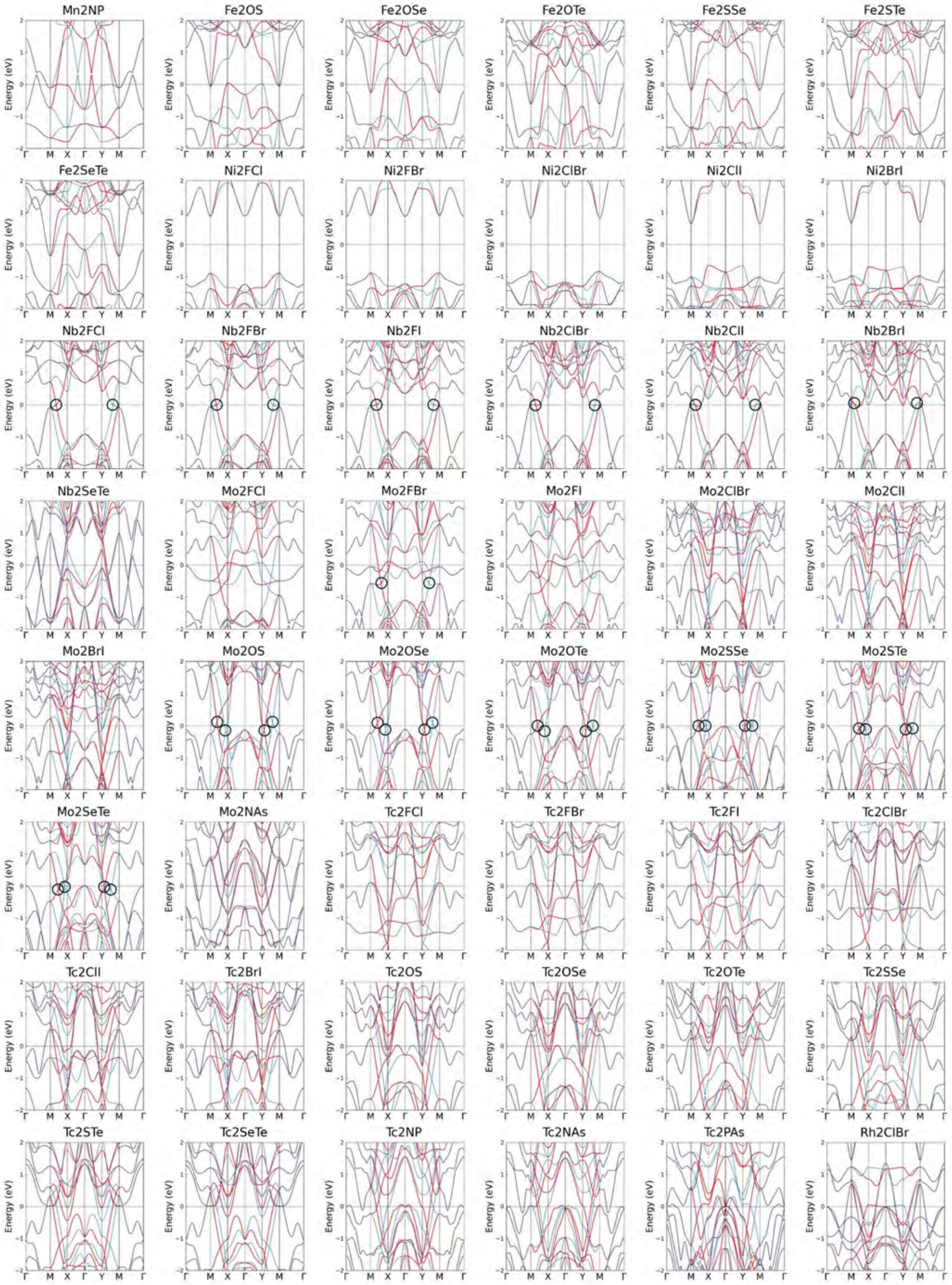

FIG. S20. Band structures of altermagnetic candidates of Janus M_2AA' framework from Mn_2NP to Rh_2ClBr.

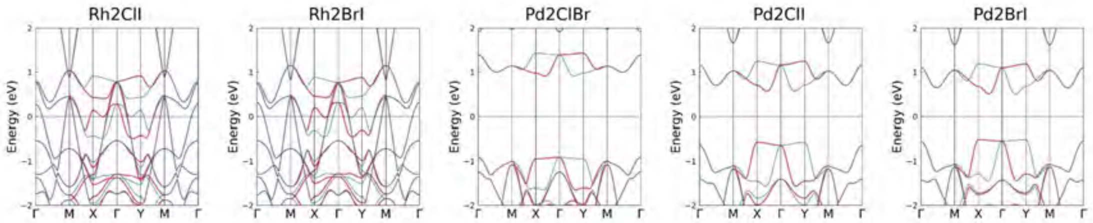

FIG. S21. Band structures of altermagnetic candidates of Janus M_2AA' framework from Rh_2ClI to Pd_2BrI.
